# High-performance topochemical polymerization-based photo-carving with sub-50 nm resolution utilizing visible light

**DOI:** 10.1038/s41467-026-73463-9

**Published:** 2026-06-25

**Authors:** Yangyang Ren, Zhenglian Qin, Yuchao Li, Yanan Zhao, Yanjun Gong, Huixian Liu, Jie Zhang, Yanke Che, Jincai Zhao, Yuchen Wu, Yifan Zhang

**Affiliations:** 1 Key Laboratory of Photochemistry, CAS Research/Education Center for Excellence in Molecular Sciences, Institute of Chemistry, Chinese Academy of Sciences, Beijing, China; 2 University of Chinese Academy of Sciences, Beijing, China; 3 Laboratory of Bio-inspired Materials and Interfacial Science, Technical Institute of Physics and Chemistry, Chinese Academy of Sciences, Beijing, China; 4 Guangdong Provincial Key Laboratory of Nanophotonic Manipulation, Institute of Nanophotonics, Jinan University, Guangzhou, China; 5 Department of Polymer Science and Engineering, University of Science and Technology of China, Hefei, China; 6 Tianjin Key Laboratory of Life and Health Detection, Life and Health Intelligent Research Institute, Tianjin University of Technology, Tianjin, China; 7 College of Chemistry, Jilin University, Changchun, China; 8 Key Laboratory of Green Catalysis of Higher Education Institutes of Sichuan, School of Chemistry and Environmental Engineering, Sichuan University of Science and Engineering, Zigong, China

**Keywords:** Polymers, Design, synthesis and processing, Surface assembly

## Abstract

Photosensitive topochemical polymerization, characterized by lattice-controlled reaction sites and rates, enables the production of highly crystalline and isotactic polymers, providing a powerful synthesis route for advancing high-performance lithographic technologies. However, the substantial challenge of achieving precise molecular packing and fabricating large-area single-crystal thin films required by topochemical reactions remains a major obstacle to realizing sub-diffraction, low-power lithographic technologies. In this study, we report a lattice-induced resolution enhancement method that exceeds the diffraction limit, in synergy with a dual solid-liquid interface confinement strategy, enabling efficient topochemical polymerization in both 2D and 3D lithographic applications. The directed fluid flow within the confined space effectively suppresses uncontrolled nucleation, facilitating the fabrication of large-area single-crystal thin-film photoresists with tunable thickness and aspect ratios. We demonstrate dual-mode photocarving on large-area photoresists at sub-50 nm resolution (*λ*/10) using a low-power continuous-wave visible laser (4-20 μW), which operates at power levels several orders of magnitude lower than those required for two-photon lithography. The entire process is compatible with standard commercial optical microscopes using standard optical components. Our research introduces a versatile platform for achieving subdiffractional lithographic resolution with low-power light, demonstrating a fivefold enhancement in resolution under identical illumination conditions compared to reported photoresists.

## Abstract (web summary)

Creating the large-area single-crystal thin films needed for photoinduced topochemical polymerisation-based photolithography is a challenge. Here, the authors use benzimidazole-containing monomers to create such films, and apply them as photoresists for high resolution photocarving using low power lasers to achieve sub-50 nm resolutions.

## Introduction

Topochemical polymerization, characterized by the lattice-controlled reaction sites and rates from identical monomer conformation and optimal intermolecular arrangement, facilitates powerful synthetic tools for linear polymers^1–6^, 2D porous framework^7–12^, and 3D polymeric single crystals^13–17^. Light-triggered topochemical polymerizations could allow for the precise spatial regulation of the polymerization process to yield highly crystalline and isotactic product. This technique has the potential to revolutionize nanofabrication processes, allowing for the creation of intricate structures in fields such as integrated circuits, MEMS, and biomedical engineering. Nevertheless, the achievement of topochemical polymerization-based photocarving has remained out of reach. The significant challenge is the difficulty in creating large-area single-crystalline film photoresists composed of photoinduced topochemical polymerizable monomers.

Monomers capable of photoinduced topochemical polymerization within a lattice are significantly rarer than those used in conventional amorphous-state polymerizations, due to the stringent requirements for precise intermolecular distances and orientations between reactive sites^16,18–20^. Successful topochemical addition reactions have been reported in crystal lattices involving diverse reactive groups, including alkynes/azide^4,21,22^, alkene/azides^23^, olefins^14,24,25^, diynes^19,26,27^, triynes^28–30^, dienes^31–33^, trienes^31^, para-quinodimethanes^34–38^, furan/maleimide^39^, alkyne/nitrile oxide^40^, and bis(indanone)s^16,41,42^. However, the morphologies and dimensions of these self-assembled single crystals in solution are challenging to precisely regulate. Common crystal forms such as plate-like, rod-like, or sheet-like structures are prone to stress accumulation during conformational changes in topochemical reactions, often resulting in fracture or cracking^43^. Although advanced assembly techniques, including blade-casting technique^44–47^, interface synthesis^48–51^, epitaxial growth^52–55^ effectively regulate organic crystal shapes through confined crystallization at solid-liquid interfaces, fabricating large-area, high-quality single-crystal films with controllable dimensions remains difficult, particularly for monomers with low solubility. The fundamental obstacle lies in the inability of a single solid-liquid interface to pin the three-phase contact line (TCL), resulting in uncontrolled mass transport^56–59^ during molecular assembly, driven by rapid TCL contraction. Uncontrolled dewetting initiates explosive nucleation, leading to disordered crystal orientation and poor crystallinity in the resulting films. Therefore, developing improved methods for fabricating large-area crystalline thin films is essential for achieving high-precision, low-energy laser engraving.

**Fig. 1:**
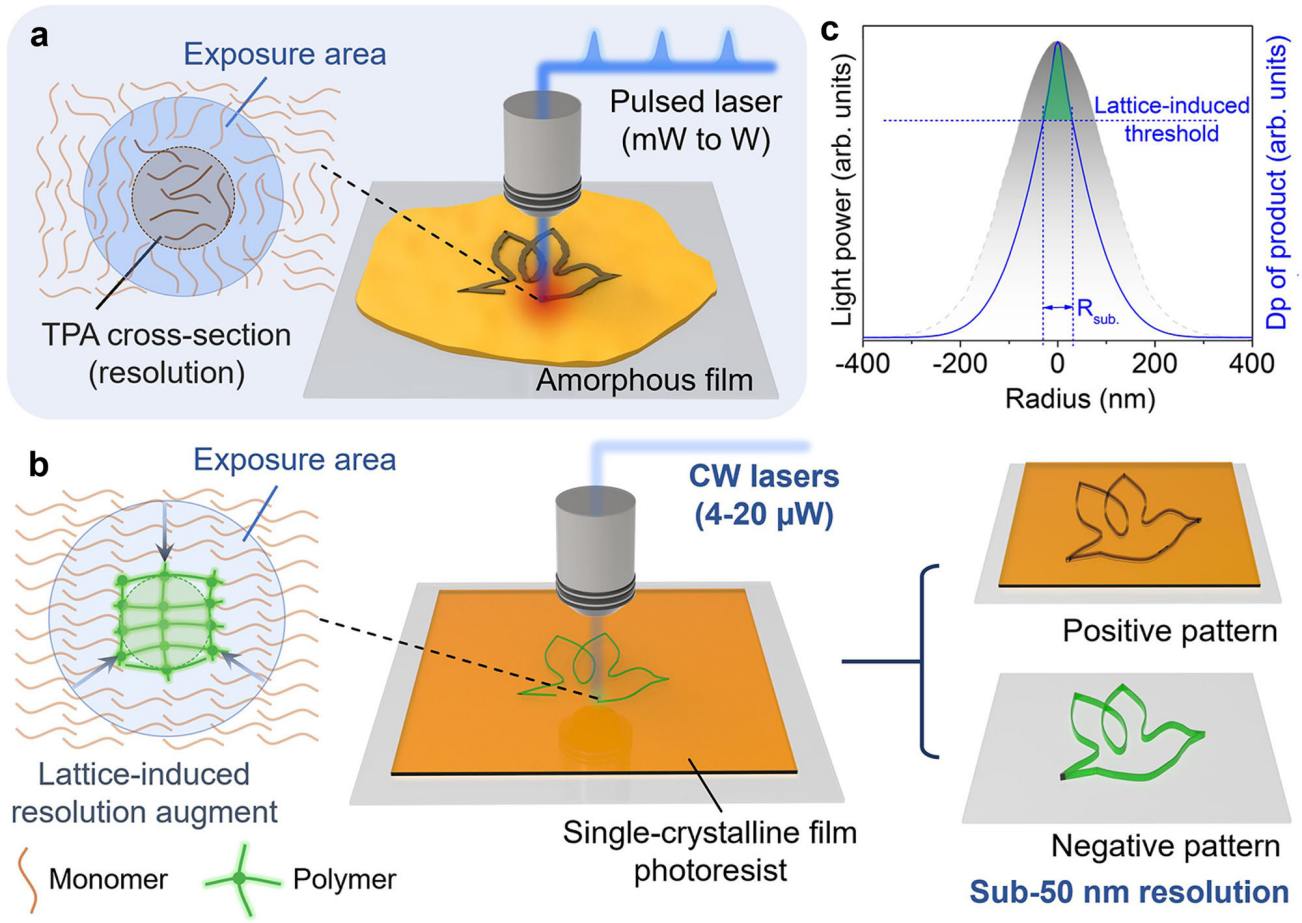
Illustration of topochemical polymerization-based photocarving using non-burning CW visible lasers. Comparison of **a**, Two-photons photolithography on amorphous film photoresist using high-power pulsed lasers. TPA two-photons absorption. **b** Topochemical polymerization-based photocarving using low power CW laser beams. **c** Lattice-induced resolution enhancement in topochemical polymerization based photocarving.

In this study, we developed topochemical polymerization-based high-performance 2D or 3D photocarving with subdiffractional resolution on a series of single-crystalline film photoresists. Photosensitive monomers comprising imidazole moieties were designed for photoinduced topochemical polymerization and preferential pre-arrangement in the form of crystalline film^60,61^. A dual solid-liquid interface-confined assembly method was developed to enable the directional mass transport of monomers, allowing for the construction of large-area single-crystalline film photoresists with independently adjustable length, width, and thickness. Sub-50 nm resolution can be achieved on 2D photocarving using low power CW visible lasers (4–20 μW, Fig. 1), which is three to five orders of magnitude lower than that used in two-photon^62–77^ or resolution augmentation through photo-induced deactivation (RAPID) lithography^78,79^. By precisely adjusting the single-crystalline film photoresists to micrometer scale, free-standing 3D nanostructures from topochemical polymerization-based 3D photocarving were successfully realized. Experimental and simulation analyses show that the lattice-induced threshold effect in topochemical polymerization confines polymerization to the light spot’s peak region, enabling sub-50 nm photocarving resolution through a nonlinear relationship between polymerization probability and polymeric product formation with sufficient degree of polymerization (Fig. 1c). The discovery of topochemical polymerization-based 2D or 3D photocarving, which breaks paradigms of subdiffractional photocarving resolution in two-photon lithography using a power threshold effect with pulsed lasers (Fig. 1a), holds significant potential in the field of non-burning, sub-50 nm nanostructures manufacturing using low power lasers and simple optical setups.

## Results and discussion

### Preparation of large-area single-crystalline film photoresists

**Fig. 2:**
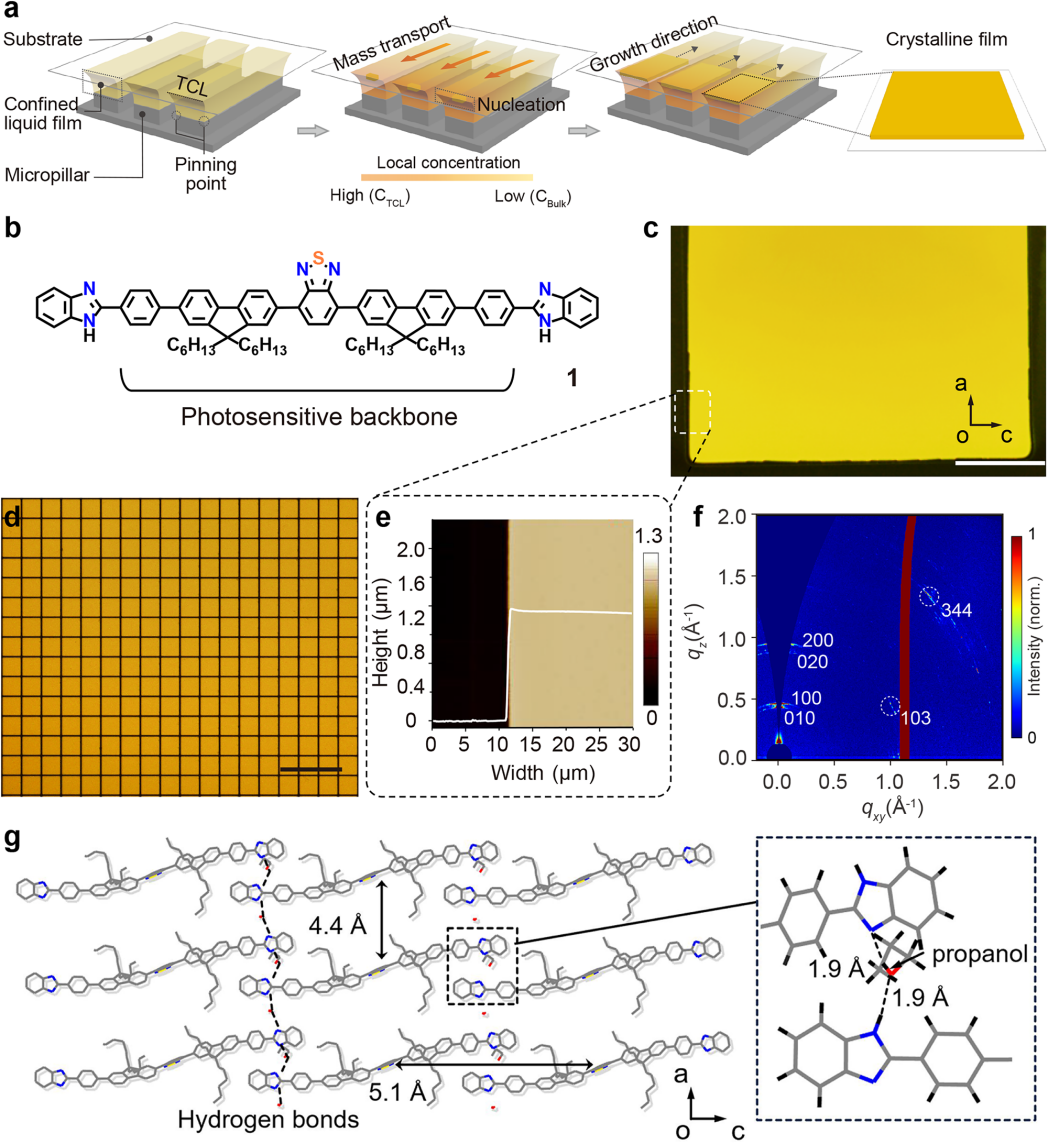
Preparation of large-area single-crystalline film photoresists. **a** Cartoon illustration of the preparation of large area single crystalline film through dual solid-liquid interface-confined assembly method. **b** Molecular structure of monomer **1**. **c** Fluorescence microscopic image of the large-area mono-patchy single-crystalline film from **1**. **d** Fluorescence microscopic images of the large-area single-crystalline film arrays with gap distance of 15 μm. **e** AFM height image of the corresponding marked region in (**c**). **f** GIWAXS image of the mono-patchy single-crystalline film from **1**. **g** Detailed packing methods in single-crystalline film photoresist from **1**. Scale bars: **c** 100 μm, **d** 500 μm.

To create single-crystalline film photoresists capable of topochemical polymerization, we designed molecular monomers composes photosensitive donor-acceptor (D-A) backbone and benzimidazole moieties on two termini. (molecule **1**, preparation details see Supplementary Information, and Supplementary Fig. 1). The photosensitive backbone combining with the benzimidazole moiety is expected to generate benzimidazole radicals upon excitation, facilitating the intermolecular coupling reactive sites within lattice. Also, the electrostatic interaction between intermolecular backbones and hydrogen bonding between diazoles applied in two distinct directions enable the monomers prearranged in the form of crystalline film. The slower kinetics of nucleation composed of two molecules compared to seeded growth^60^ allow for the well-controlled formation of crystalline film from single nucleus upon slow addition of monomers. Furthermore, to achieve large-area single-crystalline film photoresists from polymerizable monomers with low solubility, we developed a dual solid-liquid interface-confined strategy. This preparation method strictly confines the space for the self-assembly process to avoid uncontrollable multi-point nucleation of low-soluble monomers within the interface. As shown in Fig. 2a, the confined liquid film is sandwiched between the asymmetric-wettability micropillar template and the target flat substrate, forming dual solid-liquid confined interfaces and asymmetrically pinning TCL. After constituting capillary bridges pinning above the micropillars, further evaporation of organic solvents induces the rising saturated vapor pressure in the confined space, which suppress the receding of TCL and circumvent multi-point nucleation. Meanwhile, the directional capillary flows compensating for the surface liquid loss generate local high concentration at TCL through mass transport, ensuring the continuous growth of single-crystalline films with highly ordered molecular packing and uniform crystallization orientation. The high concentration zone at TCL, as demonstrated by in-situ fluorescence microscopy (Supplementary Figs. 5 and 6), highlights the directional fluid mass transfer process that governs the crystallization and crystal growth. Patchy crystalline films from **1** (Fig. 2b) arrays with millimeter-scale of each patchy were prepared on silicon substrate. Inch-scale, single-crystalline patchy film arrays can be successfully created on silicon substrate by using patterned micropillars template (Fig. 2c, d and Supplementary Fig. 7), which align with the ideal dimensions standard for the potential used in industrial photolithography. Each patchy in the single-crystalline film array exhibits a uniform thickness, with height variations within just 2 nanometers (Fig. 2e). The shape and size of the single-crystalline film patches are determined by the corresponding interfacial micropillar template (Supplementary Fig. 8). Furthermore, by adjusting the applied pressure in the confined space, the thickness of the single-crystalline film can be precisely adjusted from 100 to 2449 nm (Supplementary Fig. 9). The crystalline film from **1** exhibits a high degree of crystallographic order, as indicated by discrete grazing-incidence wide-angle X-ray scattering (GIWAXS) features (Fig. 2f) and consistent lattice parameters. The absence of Debye-Scherrer rings and unidirectional polarized fluorescence emission (Supplementary Fig. 10) suggests the lack of randomly oriented crystallites within the probed area, consistent with a single-domain or highly oriented crystalline film. Within the spatial resolution and sampling statistics of combined selected-area electron diffraction (SAED) and atomic force microscopy (AFM) measurements, no grain boundaries, orientational disorder, or subdomain formation were detected (Supplementary Figs. 11 and 12), confirming the local single-crystal characteristics. To further elucidate the detailed packing, we conducted the single-crystal diffraction analysis of the crystalline film photoresists (Supplementary Fig. 3). The structure belongs to the *P*-1 space group, with cubic cell dimensions of 14.3, 16.5, and 18.4 Å (Fig. 2g). The intraplanar packing of **1** along the *a* and *c* axes reveals intermolecular distances of 5.1 and 4.4 Å, driven by the electrostatic interactions between the photosensitive backbones and hydrogen bonding between benzimidazole moieties. The two preferential packing orientations in the film photoresists are supported by the diffraction patterns showing corresponding interplanar d-spacings (Supplementary Fig. 11). The reactive benzimidazole groups are connected by propanol through hydrogen bonds at a distance of 1.9 Å.

### Photoinduced topochemical polymerization on single-crystalline film

**Fig. 3:**
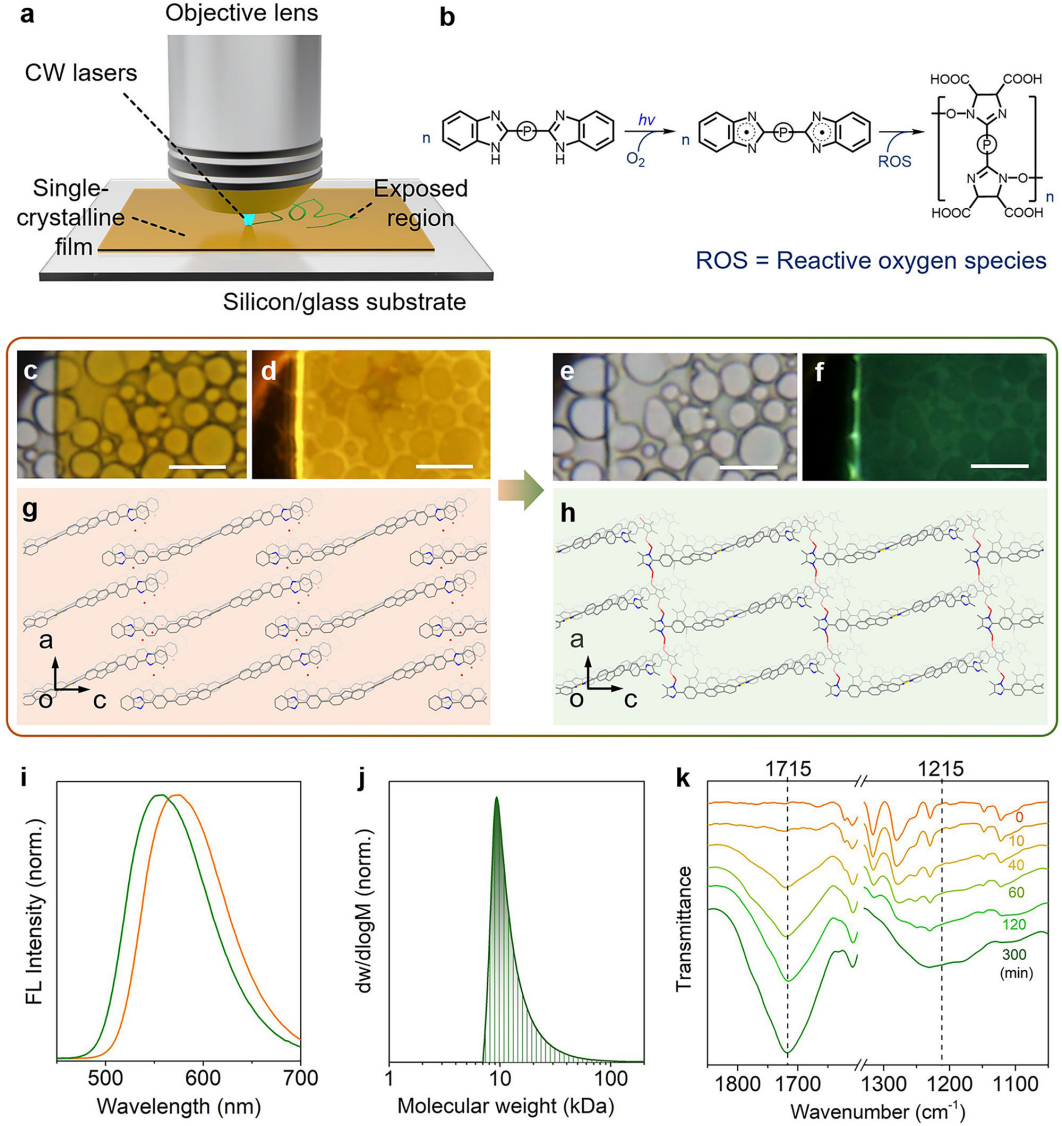
Photoinduced topochemical polymerization on large-area single-crystalline film from **1**. **a** Cartoon illustration of spatial selective photoinduced topochemical polymerization on the surface of large-area single-crystalline film using low-power CW visible lasers. **b** Photoinduced topochemical polymerization occurred in the single-crystalline film from **1**. Optical (**c**) and fluorescent microscopic images (**d**) of single-crystalline film from **1** before light exposure. The circular patches beneath the film originate from the supporting carbon film on the TEM copper grid. Scale bar = 20 μm. Optical (**e**) and fluorescent microscopic images (**f**) of single-crystalline film from **1** after light exposure. simulated molecular packing of the cross-linked polymeric film. Scale bar = 20 μm. **g,** **h** Molecular packing method of the single-crystalline film from **1** (**g**) before and (**h**) after light exposure. **i** Fluorescent spectrum of the exposed (green) and unexposed (orange) single-crystalline film from **1**. **j** Gel permeation chromatography (GPC) trance of the polymerized products in the light-exposed region (tetrahydrofuran containing [*n*-Bu_4_N]Br as the eluent). **k** In-situ infrared spectrum of the single-crystalline film from **1** under varied irradiation durations (470 nm, 50 μW).

The photoinduced reaction was investigated on the surface of the prepared large-area single-crystalline films of **1** (Fig. 3a). To match the penetration depth of the excitation light beams (visible lasers), the thickness of the single-crystalline film photoresists was set to 237 nm (Supplementary Fig. 9a). Based on the absorption spectrum of molecule **1** (Supplementary Fig. 13), a 488 nm laser was irradiated across the entire surface of the single-crystalline film (Fig. 3c) at varying power levels (1–100 µW). The optical properties of the film changed from a yellowish to a greenish fluorescent emission (Fig. 3d–f), accompanied by a significant decrease in the molar extinction coefficient. This resulted in an optical transition from light yellow to transparent (Fig. 3c–e). Spectrometric analysis revealed a blue shift in fluorescence emission from 560 to 537 nm (Fig. 3i), indicating a change in molecular planarity that increases the bandgap of the single-crystalline film. Given the possibility of photoinduced coupling reactions between imidazole moieties^80^, gel permeation chromatography (GPC) analysis was performed to identify the product after irradiation. The result showed the formation of polymeric products with a number-average molecular weight (M_n_) of 12,173 and a dispersity index (PDI) of 1.99 (Fig. 3j), corresponding to an averaged calculated polymerization degree of 10. Notably, the photoinduced polymerization of prearranged neighboring monomers **1** did not alter the surface morphology of the film (Supplementary Fig. 14). The photoinduced polymerization induces structural changes of molecule **1** during the reaction, leading to the disruption of the bridging hydrogen bonds between propanol and **1**. As a result, the hydrogen-bonded bridging propanol molecules are released into the environment as free, individual propanol molecules. The resulting morphology (Fig. 3c–e) suggests minimal changes in the cubic cell parameters after photoinduced topochemical polymerization, likely due to the close proximity (1.9 Å) of neighboring imidazole moieties pre-linked by propanol through hydrogen bonding (Fig. 3g, h). Additionally, in-situ infrared spectral analysis of the irradiated film showed enhanced vibrational peaks at 1250 cm^−1^ and 1750 cm^−1^ (Fig. 3k and Supplementary Figs. 15–16), indicating the formation of carboxylic acid moieties during the topochemical polymerization process. This was further supported by an increase in the oxygen content from 0.1 to 11 wt% after polymerization (Supplementary Fig. 17). The proposed photoinduced topochemical polymerization process in the single-crystalline film from **1** is illustrated in Fig. 3b. The formation of an acidic polymeric products and cross-linked bridging -O-O- bonds introduce sufficient polarity differences compared to the unpolymerized monomers, facilitating selective removal of the polymeric products upon irradiation. Based on this, we developed solvent developers with tailored polarities to either selectively dissolve or retain the polymeric products. Specifically, a developer solution of acetonitrile and DMSO (volume ratio = 3:1) selectively dissolves the polymeric products, whereas a 1:3:20 volume ratio of chloroform, methanol, and n-hexane preserves them. The combination of spatially selective photoinduced topochemical polymerization and developers enables the creation of large-area single-crystalline films that function as dual-mode photoresists, using continuous-wave visible lasers as photocarving beams.

**Fig. 4:**
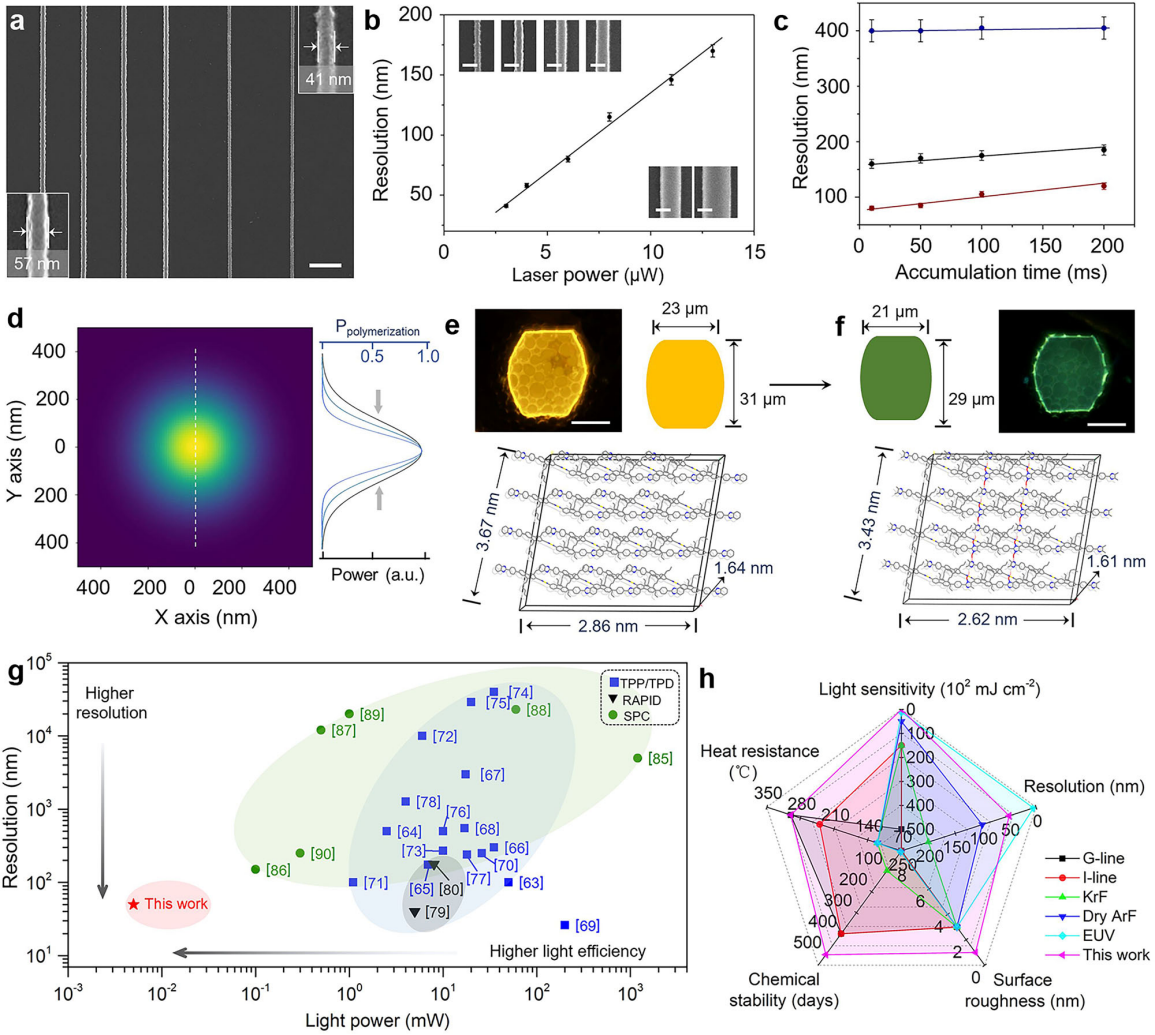
Topochemical polymerization-based photocarving performances. **a** SEM image of the negative mode photocarving linear array on silicon substrate after development. The inset is the representative nanowires with the feature line width of 57 and 41 nm. **b** The correlation between the photocarving feature width as a function of applied CW laser power. The error bars represent the standard deviation (SD). **c** The correlation between the photocarving feature width as a function of irradiation durations. The error bars represent SD. **d** The theoretical calculated effective topochemical polymerization domain to achieve polymers surpass the threshold *D*_p_ (*k* = 5, 10, and 20) within a laser spot possessing a Gaussian power distribution. **e**,** f** The microscopic and macroscopic dimensional variations of single-crystalline film from **1** before and after light exposure. **g** Comparison of the state-of-the-art photocarving performance among organic photoresists^62–79,84–89^. **h** Comparison of the photocarving performances among the existing photolithographic modalities. Scale bars, **a** 400 nm, **e**, **f** 20 μm.

To further investigate the performances of the topochemical polymerization-based photocarving, we precisely adjusted the power of the CW laser (spot diameter: ca. 400 nm), ranging from 4 to 20 μW, to perform photo-carving on the single-crystalline film photoresist from **1** with linear arrays (Supplementary Fig. 18). After selective retained the polymeric products, the developed nanostructures demonstrate optimal feature size reached as small as 41 nm with line-edge roughness (LER) of 2.9 nm (Fig. 4a and Supplementary Figs. 19 and 20). The influence of crystal face orientation along different directions of the single-crystalline film on LER is minimal (Supplementary Fig. 21). With the increasing of irradiation laser power and duration, the featuring line width showed a linear widen trend up to the laser spot size of 400 nm (Fig. 4b, c). In contrast, the spin-cast film derived from **1** failed to achieve photocarving resolution smaller than the size of the light spot (Supplementary Fig. 22). We attribute the optimal subdiffractional optimal *λ*/10 resolution arise from the threshold effect analogue to that in two-photon photolithography^81,82^, which constrain the photoreactions to the center of the light spot. The rigidity of lattice creates a laser power threshold for the occurrence of the topochemical polymerization^3,16,83^. In addition, the extensive repetition of the lattice amplifies the influence of light power on the probability of generating polymeric products with degree of polymerization (*D*_p_) surpass the *D*_p_ threshold, further boost spatial confining the effective polymerization domain. To better illustrate the lattice-induced resolution augment effect, a theoretical model was established to clarify the relationship between the *D*_p_ threshold and resolution enhancement. Specifically, we model the crystalline film photoresist as a repeating point array *P*, and represent the intermolecular cross-linking reaction by lines connecting adjacent points. When the number of points on a connected line exceeds threshold length, it is considered that polymeric products with sufficient *D*_p_ have successfully formed. For a given matrix *P*, the probability of traversing from node *i* to node *j* through *k* nodes can be expressed as *P(i, j, k)*, where *k* represents the *D*_p_ threshold. Then, a recursive relationship was used to calculate this probability (simulation details, see Supplementary Information, and Supplementary Fig. 4). Let *A* be the adjacency matrix, where *A(i, j)* indicates whether nodes *i* and *j* are adjacent. The recursive relationship can be expressed as the following Eq. 1:

1 $$P\left(i,j,k\right)={\sum }_{i,j}P\left(i,m,k-1\right)\times A(m,j)$$

Simulation result demonstrates the probability of polymeric product with sufficient *D*_p_ have non-linear relationship as a function of the intermolecular reaction probability (Supplementary Fig. 16). With the increased sufficient *D*_p_ values, the change in slope becomes more pronounced. For a Gaussian distribution of laser beam power, the area where polymerization products with a sufficient *D*_p_ form concentrates towards the center of the light spot (Fig. 4d), resulting in lattice repetition boost of photocarving resolution beyond diffraction limit without using pulsed lasers irradiation. Furthermore, the photoinduced topochemical polymerization within the lattice of single-crystalline film photoresist exhibit slight dimensional changes within 10% in each direction. For a two-dimensional platelet measuring 23 by 31 μm^2^, the size variation along the *a*-axis is 6% and the *c*-axis is 7% after forming a fully crosslinked polymeric platelet (Fig. 4e, f). These results are consistent with the variations in crystalline molecular arrangement distances predicted by microscopic simulations varied from 4.4 to 4.1 Å in *a*-axis and 5.1 to 4.7 Å in *c*-axis, respectively. The attainable sub-50 nm resolution with microwatt laser power hold advantages in terms of light efficiency and photo-carving precision compared to state-of-the-art photo-carving techniques based on two-photon polymerization/dissociation^62–77^ (TPP/TPD), RAPID^78,79^ or other single-photon induced chemical reactions (SPC^84–89^, Fig. 4g). The topochemical polymerization-based photocarving shows advantages over chemical stability, light sensitivity, and surface roughness over the existing photolithographic modalities (Fig. 4h).

### Programmed 2D and 3D nanostructures prepared by topochemical polymerization-based photocarving

**Fig. 5:**
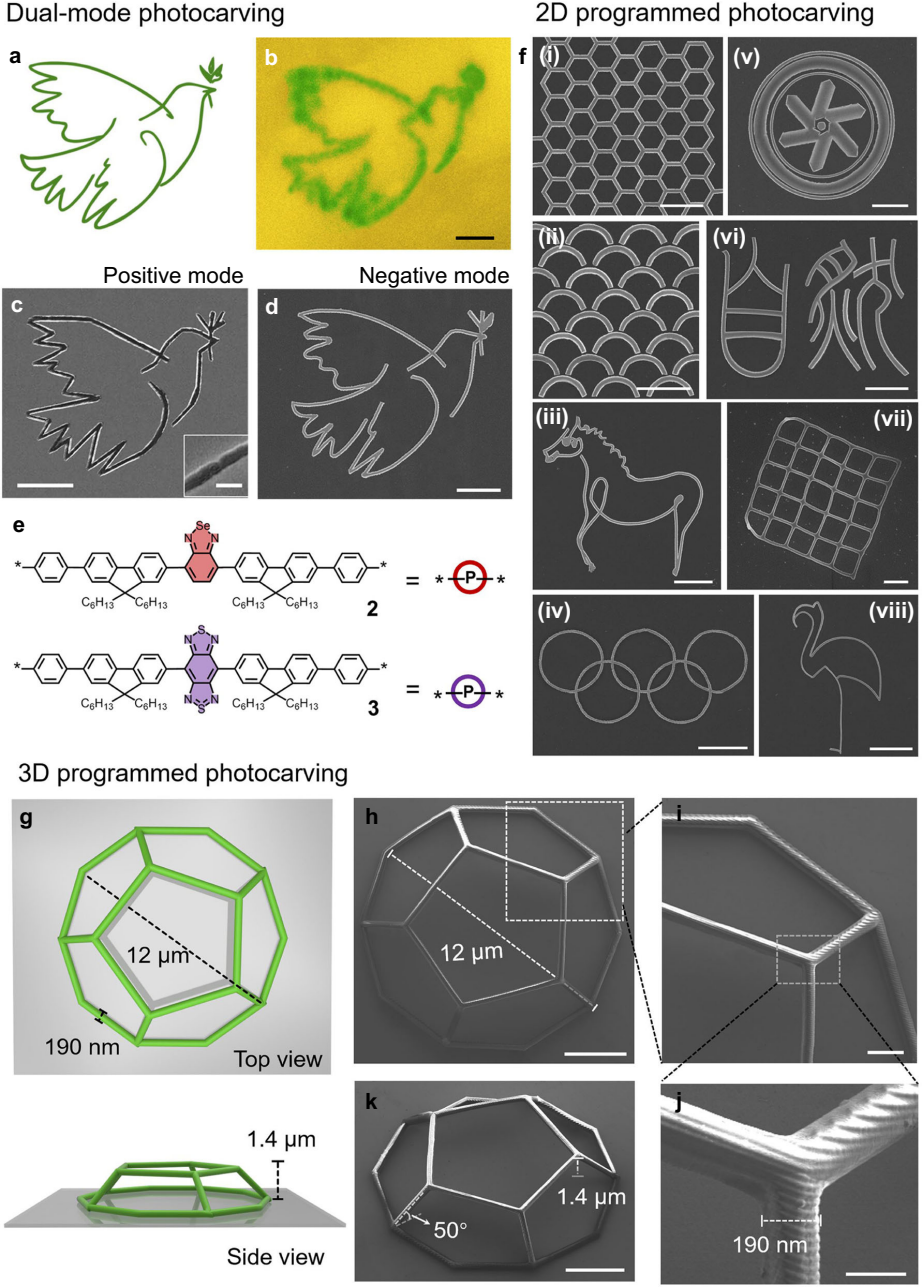
Topochemical polymerization-based dual mode programmed 2D and 3D photocarving on single-crystalline film photoresists. **a** Programmed light pattern of peace dove applied for the photocarving. **b** STED merged channel images of the photocarved single-crystalline film photoresist from **1** with peace dove pattern (470 nm, 8 μW). SEM images of attained positive mode (**c**) and negative mode (**d**) peace dove photocarved nanostructures. **e** Molecular structure of monomers **2** and **3**. **f** SEM images of attained various programmed 2D photocarved subdiffractional nanostructures, including (**i**) hexagonal pillar array, (**ii**) curved pillar array, (**iii**) horse sketch, (**iv**) five rings, (**v**) CAS logo, (**vi**) Chinese characters of nature, (**vii**) square pore grid, and (**viii**) flamingo sketch. **g** Cartoon illustration and **h**–**j** SEM images of attained 3D negative mode topochemical polymerization-based photocarved dodecahedra nanostructures imaging at various magnifications. **k** SEM images of attained 3D photocarved dodecahedra nanostructures imaging at viewing angle of 45°. Scale bars, **b** 1 μm, **c**, **d** 1 μm, 300 nm (**inlet**), **f** 1 μm, **h** 3 μm, **i** 400 nm, **j** 200 nm, **k** 3 μm.

The topochemical polymerization-based photocarving approach allows the fabrication of subdiffractional photolithographic nanostructures using low-power continuous-wave (CW) lasers with simple commercial optical setups capable of programmed light-spot positioning. As a proof of concept, leveraging a commercial confocal microscope, we achieved dual-mode 2D photocarving of subdiffractional nanostructures on large-area single-crystalline photoresist films. By steering the position of laser spot, we applied a programmed arbitrary 2D irradiation pattern (Fig. 5a) on the large-area single-crystalline film of **1** using a 488 nm laser (8 µW, spot diameter ≈ 400 nm, exposure time = 50 ms/point). As shown in Fig. 5b, the “peace dove” structure have been successfully engraved onto the single-crystalline film photoresist surface. After development, we successfully selectively prepared dual-mode 2D subdiffractional nanostructures including positive-mode nanostructures with a feature size of 90 ± 3 nm (Fig. 5c) or negative-mode patterns with a feature size of 70 ± 2 nm (Fig. 5d). To further validate the generality of topochemical polymerization-based photocarving, we expanded the monomer design to include monomers **2** and **3** (Fig. 5e) for the construction of large-area single-crystalline film photoresists. Similarly, by applying different wavelengths and power of excitation laser beams on 2D illumination patterns to single-crystalline photoresist films of monomers **1**–**3**, we successfully created dual-mode 2D photocarved nanostructures, including hexagonal/curved nanopillar arrays, horse sketch, five rings, logo of Chinese Academy of Sciences (CAS), Chinese characters, square pore grid, and flamingo sketch with optimal feature size up to 60 ± 3 nm (Fig. 5f). All the attained nanostructures exhibited uniform fluorescence emission distribution (Supplementary Fig. 23). Notably, differences in topochemical polymerization power thresholds and laser spot sizes led to minor variation on the feature sizes of attained nanostructures from photoresists composed of monomers **1**–**3**, partially arises from their distinct energy levels and molar coefficiency of corresponding applied photocarving beams.

Furthermore, since the isostatic polymeric products of topochemical polymerization exhibit enhanced mechanical strength compared to those from conventional polymerization, we extended this approach to 3D photocarving on single-crystalline photoresist films. To confine photoinduced polymerization to the focal plane, we employed pulsed infrared lasers to drive two-photon-induced polymerization. To accommodate the greater penetration depth required for 3D photocarving, we adjusted the film thickness to 2449 nm (Supplementary Fig. 9). As illustrated in Fig. 5g, a pulsed laser beam (1030 nm, pulse width: 400 fs, repetition rate: 0.1 MHz) was programmed to trace a dodecahedron pattern in 3D space, initiating two-photon polymerization. After development, a negative-mode 3D dodecahedron lattice nanostructure was obtained (Fig. 5h-j), with structural uniformity confirmed by its consistent lines and smooth turns. The fabricated dodecahedron structures exhibited a line resolution of 193 nm, and the angle between the self-supporting frame skeleton and the substrate reached 50° (Fig. 5k). In contrast, thick spin-cast films failed to produce free-standing 3D nanostructures under the same conditions (Supplementary Fig. 24).

### Mechanistic topochemical polymerization insights

To gain deeper insights on topochemical polymerization-based photocarving, we studied the polymerization mechanism of the single-crystalline film photoresists. Upon light excitation, the generation of benzimidazole radical intermediates initiates the cross-linking reaction, which can be confirmed by an emerged transient broad positive absorption peak spanning from 400 to 800 nm at 40 ps after illumination, with a prolonged lifetime to the millisecond scale^80,90^ (Supplementary Fig. 25). In control, single crystal from **4** (Supplementary Fig. 2), which lacks benzimidazole groups, showed that the photoreactions could not be triggered under the same irradiation conditions (Supplementary Figs. 26 and 27). The formation of carboxylic acid moieties during the polymerization arises from the oxidation of reactive oxygen spices (ROS) accompanied by the reaction between the benzimidazole radicals and environmental oxygen^91^. The formation of ROS, including singlet oxygen, superoxide radicals, and hydrogen peroxide, was confirmed through ESR analysis, the generation of peroxycarboxylic acids, and CeSO_4_ colorimetric assays, respectively (Supplementary Figs. 28–30). In contrast, no topochemical polymerization occurred on single-crystalline film photoresists in the absence of oxygen (Supplementary Fig. 31). The detailed topochemical polymerization process within the single-crystalline film photoresists can be conclusively depicted in Supplementary Figs. 32 and 33.

In conclusion, we have successfully created an emerging class of single-crystalline film photoresists, enabling photocarving through photosensitive topochemical polymerization to achieve sub-50 nm resolution using non-burning CW visible lasers. By leveraging the isostatic polymeric lattice formed by efficient topochemical photopolymerization, mechanically robust 3D photocarved objects with programmed nano-precision structures can be realized. The application of topological polymerization in high-precision photocarving represents a divergent strategy for developing next-generation photocarving techniques, advancing beyond subdiffractional visible light photocarving that rely on multi-photon effects. This approach holds immense potential in the realm of non-burning, high-resolution manufacturing using simple light beam setups.

## Methods

All materials were purchased from Sigma-Aldrich, Aladine, and Alfa Aesar used as received unless otherwise stated. All solvents used were of HPLC grade. Monomers **1-4** were prepared following the detailed procedures described in the Supplementary Information.

### Nuclear magnetic resonance (NMR)

^1^H NMR spectra were taken with a Bruker 300 or 500 MHz spectrometer using CDCl_3_ as solvent and chemical shifts were referenced to the residual solvent peak.

### Gel permeation chromatography (GPC)

GPC was conducted on Agilent PL-GPC50 equipped with a UV detector operating at 400 nm and a refractometer. Measurements were carried out at 1.0 mL min^−1^ with THF containing [*n*-Bu_4_N]Br (0.1% w/w) as the eluent at 35 °C, results were measured against polystyrene standards.

### Matrix-assisted laser desorption/ionization time-of-flight (MALDI-TOF)

(MALDI-TOF) mass spectrometry was performed on a Bruker UltrafleXtreme 4700 instrument. Using a small aliquot of the sample in THF (1 mg/mL) 10 μL with 100 μL of matrix solution trans-2-[3-(4-tert-butylphenyl)-2-methyl-2-propenylidene]malononitrile in THF (20 mg/mL), approximately 1 μL of this mixture was deposited on a polished steel plate and allowed to dry.

### Transmission electron microscopy (TEM)

TEM images were obtained using JEOL electron microscopes after solvent evaporation. Low-resolution images were obtained on a JEOL JEM 1400 TEM equipped with a Gatan SC200 camera, operative at 140 kV. Samples were analysed by drop-casting one drop (ca. 8 µL) of the colloidal micelle solution onto a carbon-coated copper grid placed upon a piece of filter paper to remove excess solvent. Copper grids (400 mesh) were purchased from Agar Scientific, and carbon films were prepared using an Agar TEM Turbo Carbon Coater. Carbon films were deposited onto the copper grids by floatation on water and allowed to air dry overnight.

### Powder X-ray diffraction (PXRD)

PXRD measurements were performed on a PANalytical X’Pert PRO instrument (40 kV, 200 mA).

### Single crystal X-ray diffraction (SXRD)

SXRD analysis was carried out on a Rigaku XtaLAB PRO 007HF diffractometer equipped with graphite monochromatized Mo-Kα (*λ* = 0.71073 Å) radiation in the ω scan mode. The structure was solved by intrinsic phasing (SHELXT) and refined by full-matrix least squares on F2 (SHELXL-2014).

### Scanning electron microscopy (SEM)

SEM images were recorded on a Hitachi S-8010 and TESCAN MIRA LMS field-emission microscope.

### Atomic force microscopy (AFM)

AFM images were obtained in ambient conditions in the dry state utilizing a Multimode VIII with a Nanoscope V controller with PeakForce feedback control. Single-crystalline films on mica were used for AFM measurements. All AFM images from which dimensions were determined were obtained with a fresh SCANASYST-HR (Bruker) cantilever of nominal tip radius 2 nm. Images were analyzed using Gwyddion, an open source software program for SPM images.

### Fluorescence-mode optical microscopy

Fluorescence-mode optical microscopic images were obtained from an inverted fluorescence microscope (Olympus X71).

### Absorption spectrometry measurements

UV-vis spectra of single-crystalline film photoresists from molecules **1** to **3** were obtained using a UV-Visible-NIR microscope (CRAIC Technologies, Inc.). The samples were drop-cast onto a glass substrate and dried in air.

### Selective area emission spectrometry measurements

Selective area fluorescence spectra of single-crystalline film photoresists from molecules **1** to **3** were obtained on an Olympus FV1000 confocal scanning laser microscopy. Fluorescence spectra are obtained through confocal fluorescence spectrum scanning and stitching spectral mode. The sample is excited with 405 nm light to scan fluorescence emission spectra from 500 to 700 nm. The spectral scanning has a single-step increment of 1 nm and a step size of 10 nm.

### In-situ infrared absorption spectrometry measurements

In-situ infrared (IR) absorption measurement were obtained on a Bruker Vertex 70 V spectrometer equipped with narrow-band HgCdTe detector cooled by liquid nitrogen. The samples were drop-casted on the surface of a KBr crystal plate (size 15 × 10 × 4 mm^3^) and dried over nitrogen before measurements. In-situ Fourier transform infrared (FTIR) spectra were recorded on a Bruker Vertex 70 V spectrometer equipped with narrow-band HgCdTe detector cooled by liquid nitrogen. The diffusion IR mode (DRIFTs) was employed and connected to an evacuation line (∼0 hPa). The light resource was a 150 mW continuous diode laser (375 nm). The in-situ IR spectrum with a spectralresolution of 4 cm^−1^ and scanning velocity of 160 KHz was collected in both of the dark and illumination period.

### Stimulated emission depletion (STED) microscopy

Time-gated STED imaging was carried out on a Leica TCS SP8 STED 3× microscope running LAS X acquisition software using a 100× HC PL APO CS2 oil immersion objective (numerical aperture, NA = 1.4). The photocarved on the surface of cover glass were imaged using the 470 nm line (3%) from a pulsed laser excitation (operating at 10% of its nominal power). Fluorescence depletion was accomplished using a home-built coupled continuous wave 592 nm STED laser (0.5 W nominal power, 20%). All images were acquired at a scan speed of 400 Hz in monodirectional scan mode with a pixel size of 30.4 nm^2^. The fluorescence signals were detected by a Hybrid Detector (HyD, Standard mode) after passing through an Acousto Optical Beam Splitter (AOBS). Time-gated detection (0.5–6.0 ns) was used to further improve the lateral resolution. The detector gain was adjusted to prevent saturation.

### Electron paramagnetic resonance (EPR)

EPR measurements were conducted on operating the Bruker Elexsys E500 spectrometer at room temperature. The samples for EPR characterizations were prepared by dissolving 1 mg of single-crystalline film photoresists in 50 μL of DMPO solution in toluene (0.9 mM). The samples were sealed in a capillary tube and irradiated for 5 min using 470 nm LED light before measurements. The EPR acquisition parameters were as follows: microwave frequency 9.42 GHz, microwave power 5 mW (16 dB), modulation amplitude 2 gauss, conversion time of 40.96 ms, and time constant of 40.96 ms.

### Cerium sulfate Ce(SO_4_)_2_ titration

Cerium sulfate Ce(SO_4_)_2_ titration measurements were performed to determine the amount of hydrogen peroxide generated under light. The experiments were performed by measuring the absorption spectrum of a Ce(SO_4_)_2_ solution mixed with single-crystalline film photoresists crystalline films on a quartz cuvette and irradiated by 470 nm LED light for varying durations (40, 80, and 120 min).

### Flash photolysis spectrometry measurements

Flash photolysis spectrometry measurements were conducted using the Edinburgh LP980-KS Transient Absorption Spectrometer. Samples were prepared by sealing the solution of molecule **1** in an NMR test tube under argon protection (THF, 0.025 mg/mL). The samples were then excited using a transient visible laser (488 nm), and the resulting flash photolysis spectrum and kinetics were captured using either a gated intensified CCD or PMT, respectively.

### High-resolution mass spectrometry analysis (HR-MS)

HR-MS was conducted on Bruker Solarix instrument using electrospray ionization source. Measurements were carried out at 120.0 μL h^−1^ ethanol solution, with 3200 V capillary voltage.

### High-performance liquid chromatography-mass spectrometry (HPLC-MS)

HPLC-MS was performed on a UPLC SQD2 PDA instrument equipped with a BEH Amide column (1.7 µm × 2.1 mm × 100 mm). The operating conditions of the HPLC-MS were carried out at 0.1 mL min^−1^ with methanol containing ammonium hydroxide (0.1% w/w) as the eluent at 35 °C.

## Supplementary information

SUPPLEMENTARY INFORMATION

Transparent Peer Review file

## Source data

Source Data Figure

Source Data file Supplementary information

## Data Availability

All data supporting the findings of this study are available from the corresponding author upon request. Source data are provided with this paper.

## References

[1] Ravi, A., Hassan, S. Z., Bhandary, S. & Sureshan, K. M. Topochemical postulates: are they relevant for topochemical reactions occurring at elevated temperatures? *Angew. Chem. Int. Ed.* **61**, e202200954 (2022). 10.1002/anie.202200954 35258143

[2] Liu, Y., Guan, X.-R., Wang, D.-C., Stoddart, J. F. & Guo, Q.-H. Soluble and processable single-crystalline cationic polymers. *J. Am. Chem. Soc.* **145**, 13223–13231 (2023). 37294599 10.1021/jacs.3c02266

[3] Wang, M., Jin, Y., Zhang, W. & Zhao, Y. Single-crystal polymers (SCPs): from 1D to 3D architectures. *Chem. Soc. Rev.* **52**, 8165–8193 (2023). 37929665 10.1039/d3cs00553d

[4] Mohanrao, R., Hema, K. & Sureshan, K. M. Topochemical synthesis of different polymorphs of polymers as a paradigm for tuning properties of polymers. *Nat. Commun.* **11**, 865 (2020). 32054844 10.1038/s41467-020-14733-y PMC7018732

[5] Anderson, C. L. et al. Solution-processable and functionalizable ultra-high molecular weight polymers via topochemical synthesis. *Nat. Commun.* **12**, 6818 (2021). 34819494 10.1038/s41467-021-27090-1 PMC8613210

[6] Hema, K. & Sureshan, K. M. Topochemical azide–alkyne cycloaddition reaction. *Acc. Chem. Res.* **52**, 3149–3163 (2019). 31600046 10.1021/acs.accounts.9b00398

[7] Zhang, W. et al. Reconstructed covalent organic frameworks. *Nature* **604**, 72–79 (2022). 35388196 10.1038/s41586-022-04443-4 PMC8986529

[8] Sun, C., Oppenheim, J. J., Skorupskii, G., Yang, L. & Dincă, M. Reversible topochemical polymerization and depolymerization of a crystalline 3D porous organic polymer with C–C bond linkages. *Chem* **8**, 3215–3224 (2022).

[9] Auras, F. et al. Dynamic two-dimensional covalent organic frameworks. *Nat. Chem*. 10.1038/s41557-024-01527-8 (2024). 10.1038/s41557-024-01527-8 38702406

[10] Kissel, P., Murray, D. J., Wulftange, W. J., Catalano, V. J. & King, B. T. A nanoporous two-dimensional polymer by single-crystal-to-single-crystal photopolymerization. *Nat. Chem.* **6**, 774–778 (2014). 25143211 10.1038/nchem.2008

[11] Balan, H. & Sureshan, K. M. Hierarchical single-crystal-to-single-crystal transformations of a monomer to a 1D-polymer and then to a 2D-polymer. *Nat. Commun.* **15**, 6638 (2024). 39103335 10.1038/s41467-024-51051-z PMC11300669

[12] Balan, H., Shahanas, S. & Sureshan, K. M. Single-crystal-to-single-crystal synthesis of an adaptive two-dimensional polymer with dynamic pores. *J. Am. Chem. Soc.* **147**, 37798–37807 (2025). 40999682 10.1021/jacs.5c13942

[13] Dotson, J. J. et al. Taming radical pairs in the crystalline solid state: discovery and total synthesis of psychotriadine. *J. Am. Chem. Soc.* **143**, 4043–4054 (2021). 33682403 10.1021/jacs.1c01100 PMC8292139

[14] Guo, Q.-H. et al. Single-crystal polycationic polymers obtained by single-crystal-to-single-crystal photopolymerization. *J. Am. Chem. Soc.* **142**, 6180–6187 (2020). 32017550 10.1021/jacs.9b13790

[15] Pramod, T., Khazeber, R., Athiyarath, V. & Sureshan, K. M. Topochemistry for difficult peptide–polymer synthesis: single-crystal-to-single-crystal synthesis of an isoleucine-based polymer, a hydrophobic coating material. *J. Am. Chem. Soc.* **146**, 7257–7265 (2024). 38253536 10.1021/jacs.3c10779

[16] Dou, L. et al. Single-crystal linear polymers through visible light–triggered topochemical quantitative polymerization. *Science* **343**, 272–277 (2014). 24436414 10.1126/science.1245875

[17] Beaudoin, D., Maris, T. & Wuest, J. D. Constructing monocrystalline covalent organic networks by polymerization. *Nat. Chem.* **5**, 830–834 (2013). 24056338 10.1038/nchem.1730

[18] Hema, K. et al. Topochemical polymerizations for the solid-state synthesis of organic polymers. *Chem. Soc. Rev.* **50**, 4062–4099 (2021). 33543741 10.1039/d0cs00840k

[19] Lauher, J. W., Fowler, F. W. & Goroff, N. S. Single-crystal-to-single-crystal topochemical polymerizations by design. *Acc. Chem. Res.* **41**, 1215–1229 (2008). 18712885 10.1021/ar8001427

[20] Hema, K., Ravi, A., Raju, C. & Sureshan, K. M. Polymers with advanced structural and supramolecular features synthesized through topochemical polymerization. *Chem. Sci.* **12**, 5361–5380 (2021). 34168781 10.1039/d0sc07066a PMC8179609

[21] Hema, K. & Sureshan, K. M. β-Sheet to helical-sheet evolution induced by topochemical polymerization: cross-α-amyloid-like packing in a pseudoprotein with Gly-Phe-Gly repeats. *Angew. Chem. Int. Ed.* **59**, 8854–8859 (2020). 10.1002/anie.201914975 32149438

[22] Krishnan, B. P., Rai, R., Asokan, A. & Sureshan, K. M. Crystal-to-crystal synthesis of triazole-linked pseudo-proteins via topochemical azide–alkyne cycloaddition reaction. *J. Am. Chem. Soc.* **138**, 14824–14827 (2016). 27791357 10.1021/jacs.6b07538

[23] Khazeber, R. & Sureshan, K. M. Topochemical ene–azide cycloaddition reaction. *Angew. Chem. Int. Ed.* **133**, 25079–25085 (2021). 10.1002/anie.202109344 34379367

[24] Johnston, P., Braybrook, C. & Saito, K. Topochemical photo-reversible polymerization of a bioinspired monomer and its recovery and repolymerization after photo-depolymerization. *Chem. Sci.* **3**, 2301–2306 (2012).

[25] Garai, M., Santra, R. & Biradha, K. Tunable plastic films of a crystalline polymer by single-crystal-to-single-crystal photopolymerization of a diene: self-templating and shock-absorbing two-dimensional hydrogen-bonding layers. *Angew. Chem. Int. Ed*. **52**, 5548–5551 (2013). 10.1002/anie.201300690 23592325

[26] Tian, S. et al. Polydiacetylene-based ultrastrong bioorthogonal Raman probes for targeted live-cell Raman imaging. *Nat. Commun.* **11**, 81 (2020). 31900403 10.1038/s41467-019-13784-0 PMC6941979

[27] Jordan, R. S. et al. Synthesis of graphene nanoribbons via the topochemical polymerization and subsequent aromatization of a diacetylene precursor. *Chem* **1**, 78–90 (2016).

[28] Freitag, M. et al. Polymerization studies of diiodohexatriyne and diiodooctatetrayne cocrystals. *Macromolecules* **52**, 8563–8568 (2019).

[29] Xu, R., Schweizer, W. B. & Frauenrath, H. Perfluorophenyl–phenyl interactions in the crystallization and topochemical polymerization of triacetylene monomers. *Chem. Eur. J.* **15**, 9105–9116 (2009). 19637260 10.1002/chem.200900985

[30] Xiao, J., Yang, M., Lauher, J. W. & Fowler, F. W. A supramolecular solution to a long-standing problem: The 1, 6-polymerization of a triacetylene. *Angew. Chem. Int. Ed.* **112**, 2216–2219 (2000). 10.1002/1521-3773(20000616)39:12<2132::aid-anie2132>3.0.co;2-8 10941039

[31] Hou, X. et al. Synthesis of polymeric ladders by topochemical polymerization. *Chem. Comm.* **50**, 1218–1220 (2014). 24336342 10.1039/c3cc47379a

[32] Tanaka, T. & Matsumoto, A. First disyndiotactic polymer from a 1, 4-disubstituted butadiene by alternate molecular stacking in the crystalline state. *J. Am. Chem. Soc.* **124**, 9676–9677 (2002). 12175204 10.1021/ja020615r

[33] Nagahama, S. & Matsumoto, A. Synchronized propagation mechanism for crystalline-state polymerization of p-xylylenediammonium disorbate. *J. Am. Chem. Soc.* **123**, 12176–12181 (2001). 11734016 10.1021/ja011575e

[34] Itoh, T. et al. Formation of bundle assemblies of stereoregular polymers in thermal solid-state polymerization of 7, 7, 8, 8-tetrakis (aryloxycarbonyl)-p-quinodimethanes. *Macromolecules* **49**, 4802–4816 (2016).

[35] Itoh, T. et al. Cis-specific topochemical polymerization: alternating copolymerization of 7, 7, 8, 8-tetrakis (methoxycarbonyl) quinodimethane with 7, 7, 8, 8-tetracyanoquinodimethane in the solid state. *Angew. Chem. Int. Ed.* **10**, 2301–2304 (2011). 10.1002/anie.201006928 21351330

[36] Nomura, S. et al. Crystal structures and topochemical polymerizations of 7, 7, 8, 8-tetrakis (alkoxycarbonyl) quinodimethanes. *J. Am. Chem. Soc.* **126**, 2035–2041 (2004). 14971937 10.1021/ja0386086

[37] Itoh, T. et al. Solid-state polymerizations of 7-alkoxycarbonyl-7-cyano-1, 4-benzoquinone methides. *Macromolecules* **37**, 7938–7944 (2004).

[38] Itoh, T. et al. Topochemical Polymerization of 7, 7, 8, 8-Tetrakis (methoxycarbonyl) quinodimethane. *Angew. Chem. Int. Ed.* **41**, 4306–4309 (2002). 10.1002/1521-3773(20021115)41:22<4306::AID-ANIE4306>3.0.CO;2-W 12434371

[39] Pathak, S. & Sureshan, K. M. A syndiotactic polymer via spontaneous exoselective single-crystal-to-single-crystal topochemical diels–alder cycloaddition reaction. *J. Am. Chem. Soc.* **146**, 30495–30501 (2024). 39450511 10.1021/jacs.4c11426

[40] Singh, R., Siddharthan, A. & Sureshan, K. M. Topochemical alkyne nitrile oxide cycloaddition for polymer synthesis. *Angew. Chem. Int. Ed.* **64**, e20947 (2025). 10.1002/anie.202520947 41178248

[41] Samanta, R. et al. Mechanical actuation and patterning of rewritable crystalline monomer− polymer heterostructures via topochemical polymerization in a dual-responsive photochromic organic material. *ACS Appl. Mater. Interfaces* **12**, 16856–16863 (2020). 32162514 10.1021/acsami.9b23189

[42] Samanta, R., Ghosh, S., Devarapalli, R. & Reddy, C. M. Visible light mediated photopolymerization in single crystals: photomechanical bending and thermomechanical unbending. *Chem. Mater.* **30**, 577–581 (2018).

[43] Pathan, J. R. et al. A self-healing crystal that repairs multiple cracks. *J. Am. Chem. Soc.* **146**, 27100–27108 (2024). 39292954 10.1021/jacs.4c09334 PMC11457417

[44] Xiong, Z., Liao, C., Han, W. & Wang, X. Mechanically tough large-area hierarchical porous graphene films for high-performance flexible supercapacitor applications. *Adv. Mater.* **27**, 4469–4475 (2015). 26135240 10.1002/adma.201501983

[45] Zhang, Z. et al. Improved osmotic energy conversion in heterogeneous membrane boosted by three-dimensional hydrogel interface. *Nat. Commun.* **11**, 875 (2020). 32054863 10.1038/s41467-020-14674-6 PMC7018769

[46] Alvarez, L. et al. Reconfigurable artificial microswimmers with internal feedback. *Nat. Commun.* **12**, 4762 (2021). 34362934 10.1038/s41467-021-25108-2 PMC8346629

[47] Zhang, L., Lin, B., Hu, B., Xu, X. & Ma, W. Blade-cast nonfullerene organic solar cells in air with excellent morphology, efficiency, and stability. *Adv. Mater.* **30**, 1800343 (2018). 10.1002/adma.201800343 29665119

[48] Gobbi, M. et al. Graphene transistors for real-time monitoring molecular self-assembly dynamics. *Nat. Commun.* **11**, 4731 (2020). 32948763 10.1038/s41467-020-18604-4 PMC7501237

[49] Jeong, G. H. et al. Nanoscale Assembly of 2D Materials for Energy and Environmental Applications. *Adv. Mater.* **32**, 1907006 (2020). 10.1002/adma.201907006 32243010

[50] Begley, M. R., Gianola, D. S. & Ray, T. R. Bridging functional nanocomposites to robust macroscale devices. *Science* **364**, eaav4299 (2019). 31249029 10.1126/science.aav4299

[51] Hou, J., Li, M. & Song, Y. Patterned colloidal photonic crystals. *Angew. Chem. Int. Ed.* **57**, 2544–2553 (2018). 10.1002/anie.201704752 28891204

[52] Lv, Q. et al. Lateral epitaxial growth of two-dimensional organic heterostructures. *Nat. Chem.* **16**, 201–209 (2024). 38036642 10.1038/s41557-023-01364-1

[53] Zhang, Y. et al. Controlled formation of three-dimensional cavities during lateral epitaxial growth. *Nat. Commun.* **15**, 2247 (2024). 38472172 10.1038/s41467-024-46222-x PMC10933262

[54] Zou, Y. et al. Epitaxial growth of metal-organic framework nanosheets into single-crystalline orthogonal arrays. *Nat. Commun.* **14**, 5780 (2023). 37723168 10.1038/s41467-023-41517-x PMC10507060

[55] Leonhardt, E. J. et al. A bottom-up approach to solution-processed, atomically precise graphitic cylinders on graphite. *Nano Lett.* **18**, 7991–7997 (2018). 30480454 10.1021/acs.nanolett.8b03979

[56] Chen, J., Qin, S., Wu, X., Chu & Paul, K. Morphology and pattern control of diphenylalanine self-assembly via evaporative dewetting. *ACS Nano* **10**, 832–838 (2016). 26654935 10.1021/acsnano.5b05936

[57] Nerger, B. A., Brun, P.-T. & Nelson, C. M. Marangoni flows drive the alignment of fibrillar cell-laden hydrogels. *Sci. Adv.* **6**, eaaz7748 (2020). 32582851 10.1126/sciadv.aaz7748 PMC7292634

[58] Yen, T. M. et al. Reversing coffee-ring effect by laser-induced differential evaporation. *Sci. Rep.* **8**, 3157 (2018). 29453347 10.1038/s41598-018-20581-0 PMC5816656

[59] Maheshwari, S., Zhang, L., Zhu, Y. & Chang, H.-C. Coupling between precipitation and contact-line dynamics: multiring stains and stick-slip motion. *Phys. Rev. Lett.* **100**, 044503 (2008). 18352284 10.1103/PhysRevLett.100.044503

[60] Gong, Y. et al. Unprecedented small molecule-based uniform two-dimensional platelets with tailorable shapes and sizes. *J. Am. Chem. Soc.* **144**, 15403–15410 (2022). 35952365 10.1021/jacs.2c07480

[61] Gong, Y. et al. Fabrication of two-dimensional platelets with heat-resistant luminescence and large two-photon absorption cross sections via cooperative solution/solid self-assembly. *J. Am. Chem. Soc.* **145**, 9771–9776 (2023). 37079712 10.1021/jacs.3c01517

[62] Yu, Y., Prudnikau, A., Lesnyak, V. & Kirchner, R. Quantum dots facilitate 3D two-photon laser lithography. *Adv. Mater.* **35**, 2211702 (2023). 10.1002/adma.202211702 37042293

[63] Tang, J. et al. Ketocoumarin-based photoinitiators for high-sensitivity two-photon lithography. *ACS Appl. Polym. Mater.* **5**, 2956–2963 (2023).

[64] Saha, S. K. et al. Scalable submicrometer additive manufacturing. *Science* **366**, 105–109 (2019). 31604310 10.1126/science.aax8760

[65] Luitz, M. et al. High resolution patterning of an organic–inorganic photoresin for the fabrication of platinum microstructures. *Adv. Mater.* **33**, 2101992 (2021). 34337801 10.1002/adma.202101992 PMC11469048

[66] Liu, K. et al. 3D printing colloidal crystal microstructures via sacrificial-scaffold-mediated two-photon lithography. *Nat. Commun.* **13**, 4563 (2022). 35931721 10.1038/s41467-022-32317-w PMC9355982

[67] Limberg, D. K., Kang, J.-H. & Hayward, R. C. J. J. O. T. A. C. S. Triplet–triplet annihilation photopolymerization for high-resolution 3D printing. *J. Am. Chem. Soc.* **144**, 5226–5232 (2022). 35285620 10.1021/jacs.1c11022

[68] Jin, F. et al. λ/30 inorganic features achieved by multi-photon 3D lithography. *Nat. Commun.* **13**, 1357 (2022). 35292637 10.1038/s41467-022-29036-7 PMC8924217

[69] Jaiswal, A. et al. Additive-free all-carbon composite: a two-photon material system for nanopatterning of fluorescent sub-wavelength structures. *ACS Nano* **15**, 14193–14206 (2021). 34435496 10.1021/acsnano.1c01083

[70] Guan, L. et al. Light and matter co-confined multi-photon lithography. *Nat. Commun.* **15**, 2387 (2024). 38493192 10.1038/s41467-024-46743-5 PMC10944545

[71] Gernhardt, M., Truong, V. X. & Barner-Kowollik, C. Visible-light-degradable 3D microstructures in aqueous environments. *Adv. Mater.* **34**, 2203474 (2022). 10.1002/adma.202203474 35918791

[72] Geng, Q., Wang, D., Chen, P. & Chen, S.-C. Ultrafast multi-focus 3-D nano-fabrication based on two-photon polymerization. *Nat. Commun.* **10**, 2179 (2019). 31097713 10.1038/s41467-019-10249-2 PMC6522551

[73] Dong, Z. et al. Material-independent 3D patterning via two-photon lithography and discontinuous wetting. *Adv. Mater. Technol.* **8**, 2201268 (2023).

[74] Chan, J. Y. E. et al. High-resolution light field prints by nanoscale 3D printing. *Nat. Commun.* **12**, 3728 (2021). 34140502 10.1038/s41467-021-23964-6 PMC8211842

[75] Batchelor, R. et al. Two in one: light as a tool for 3D printing and erasing at the microscale. *Adv. Mater.* **31**, 1904085 (2019). 10.1002/adma.201904085 31420930

[76] Abdallah, S. et al. Reversible optical data storage via two-photon micropatterning of o-carboranes-embedded switchable materials. *Chem. Mater.* **35**, 6979–6989 (2023).

[77] Li, Q. et al. Mechanical nanolattices printed using nanocluster-based photoresists. *Science* **378**, 768–773 (2022). 36395243 10.1126/science.abo6997

[78] Li, L., Gattass, R. R., Gershgoren, E., Hwang, H. & Fourkas, J. T. Achieving λ/20 resolution by one-color initiation and deactivation of polymerization. *Science* **324**, 910–913 (2009). 19359543 10.1126/science.1168996

[79] Stocker, M. P., Li, L., Gattass, R. R. & Fourkas, J. T. Multiphoton photoresists giving nanoscale resolution that is inversely dependent on exposure time. *Nat. Chem.* **3**, 223–227 (2011). 21336328 10.1038/nchem.965

[80] Hatano, S., Horino, T., Tokita, A., Oshima, T. & Abe, J. Unusual negative photochromism via a short-lived imidazolyl radical of 1,1′-binaphthyl-bridged imidazole dimer. *J. Am. Chem. Soc.* **135**, 3164–3172 (2013). 23402262 10.1021/ja311344u

[81] Kawata, S., Sun, H.-B., Tanaka, T. & Takada, K. Finer features for functional microdevices. *Nature* **412**, 697–698 (2001). 11507627 10.1038/35089130

[82] Liao, Y. et al. Femtosecond laser nanostructuring in porous glass with sub-50 nm feature sizes. *Opt. Lett.* **38**, 187–189 (2013). 23454957 10.1364/OL.38.000187

[83] Gong, Y. et al. Molecular interactions control quantum chain reactions toward distinct photoresponsive properties of molecular crystals. *J. Am. Chem. Soc.* **139**, 10649–10652 (2017). 28749145 10.1021/jacs.7b06261

[84] Shin, J. et al. Monolithic digital patterning of polydimethylsiloxane with successive laser pyrolysis. *Nat. Mater.* **20**, 100–107 (2021). 32807919 10.1038/s41563-020-0769-6

[85] Hahn, V. et al. Two-step absorption instead of two-photon absorption in 3D nanoprinting. *Nat. Photon.* **15**, 932–938 (2021).

[86] Borchers, T. et al. Cold photo-carving of halogen-bonded co-crystals of a dye and a volatile co-former using visible light. *Nat. Chem.* **14**, 574–581 (2022). 35361911 10.1038/s41557-022-00909-0

[87] Zhao, X. et al. Efficient 3D printing via photooxidation of ketocoumarin based photopolymerization. *Nat. Commun.* **12**, 2873 (2021). 34001898 10.1038/s41467-021-23170-4 PMC8129151

[88] Borchers, T. H. et al. Three-in-one: dye-volatile cocrystals exhibiting intensity-dependent photochromic, photomechanical, and photocarving response. *J. Am. Chem. Soc.* **145**, 24636–24647 (2023). 37924293 10.1021/jacs.3c07060 PMC10655124

[89] Kravchenko, A., Shevchenko, A., Ovchinnikov, V., Priimagi, A. & Kaivola, M. Optical interference lithography using azobenzene-functionalized polymers for micro- and nanopatterning of silicon. *Adv. Mater.* **23**, 4174–4177 (2011). 21823180 10.1002/adma.201101888

[90] Ito, H., Mutoh, K. & Abe, J. Bridged-imidazole dimer exhibiting three-state negative photochromism with a single photochromic unit. *J. Am. Chem. Soc.* **145**, 6498–6506 (2023). 36888966 10.1021/jacs.3c00476

[91] Brusina, M. A., Gubina, Y. A., Nikolaev, D. N., Ramsh, S. M. & Piotrovskii, L. B. Influence of oxidation conditions on the yield of 2-substituted imidazole-4,5-dicarboxylic Acids. *Russ. J. Gen. Chem.* **88**, 874–878 (2018).

