# Supplementary material for: High-performance topochemical polymerization-based photo-carving with sub-50 nm resolution utilizing visible light
Source: Nat Commun. 2026 Jun 25;17:7964. doi: 10.1038/s41467-026-73463-9 (PMC13448494; doi:10.1038/s41467-026-73463-9)
Supplement: Supplementary file 1 [file 41467_2026_73463_MOESM1_ESM.pdf]

Supplementary Information for

High-performance topochemical polymerization-based photocarving with sub-50 nm resolution utilizing visible light

Yangyang Ren[1,3,#], Zhenglian Qin[2,3,#], Yuchao Li[4,#], Yanan Zhao[4], Yanjun Gong[1,3], Huixian Liu[5], Jie Zhang[5], Yanke Che[1,6*], Jincai Zhao[1,3], Yuchen Wu[2,3,7*], and Yifan Zhang[1,3,8*].

Affiliations:

[1]Key Laboratory of Photochemistry, CAS Research/Education Center for Excellence in Molecular Sciences, Institute of Chemistry, Chinese Academy of Sciences, Beijing 100190, China.

[2]Laboratory of Bio-inspired Materials and Interfacial Science, Technical Institute of Physics and Chemistry, Chinese Academy of Sciences, Beijing 100190, China.

[3]University of Chinese Academy of Sciences, Beijing 100049, China.

[4]Guangdong Provincial Key Laboratory of Nanophotonic Manipulation, Institute of Nanophotonics, Jinan University, Guangzhou 511443, China.

[5]Department of Polymer Science and Engineering, University of Science and Technology of China, 96 Jinzhai Rd, Hefei, Anhui, 230026, China.

[6]Tianjin Key Laboratory of Life and Health Detection, Life and Health Intelligent Research Institute, Tianjin University of Technology, Tianjin 300384, P. R. China.

[7]College of Chemistry, Jilin University, Changchun 130012, China.

[8]Key Laboratory of Green Catalysis of Higher Education Institutes of Sichuan, School of Chemistry and Environmental Engineering, Sichuan University of Science and Engineering, Zigong 643000, P. R. China.

*Correspondence to: ykche@iccas.ac.cn (Y.K. Che), yuchenwu@iccas.ac.cn (Y.C. Wu), yfzhang@iccas.ac.cn (Y.F. Zhang)

[#]These authors contributed equally to this work.

Table of Contents:

Supplementary Fig. 1: Molecular structures of **1** to **4**.
Supplementary Fig. 2: ^{1}H NMR and MALDI-MS of molecule **4**.
Supplementary Fig. 3: Comparison of X-ray diffraction (XRD) patterns.
Supplementary Fig. 4: Simulation details.
Supplementary Fig. 5: The dewetting progress in a Sandwich-type structure.
Supplementary Fig. 6: Asymmetric-wettability micropillar templates.
Supplementary Fig. 7: Fluorescence optical microscopy images.
Supplementary Fig. 8: AFM images of different shapes single-crystalline film.
Supplementary Fig. 9: AFM images of precise thickness modulation.
Supplementary Fig. 10: Polarization property.
Supplementary Fig. 11: Bright-field TEM and corresponding SAED pattern.
Supplementary Fig. 12. AFM imaging of single-crystalline film from **1.**
Supplementary Fig. 13. UV-vis absorption spectrum of solution and film from **1.**
Supplementary Fig. 14: Topochemical polymerization-based photocarving.
Supplementary Fig. 15: The formation of oxidized polymers.
Supplementary Fig. 16: The kinetics of the formation of oxidized polymers.
Supplementary Fig. 17: Elemental analysis of single-crystalline film photoresists.
Supplementary Fig. 18: Programmable photocarving.
Supplementary Fig. 19: Preparation of photocarved dense line patterns.
Supplementary Fig. 20: Cross-sectional morphology of photocarved line patterns.
Supplementary Fig. 21: Comparison of LER along different facet directions.
Supplementary Fig. 22: Photocarving of amorphous film from **1**.
Supplementary Fig. 23: The development process of photocarved patterns.
Supplementary Fig. 24: 3D photocarving on spin-cast amorphous film.
Supplementary Fig. 25: Excited-state species investigation.
Supplementary Fig. 26: Photocarving on crystals from **4**.
Supplementary Fig. 27: Photoreactions investigation of molecule **4**.
Supplementary Fig. 28: The radical species of the photopolymerization.
Supplementary Fig. 29: The formation of oxidized products.
Supplementary Fig. 30: Hydrogen dioxide detection.
Supplementary Fig. 31: Photoirradiation of single-crystalline film from **1** in argon.
Supplementary Fig. 32. *In-situ* infrared analysis of the reaction process.
Supplementary Fig. 33: Detailed mechanism.
Supplementary References

Synthesis of monomers

The synthesis of monomers (molecules **1** to **4**) was performed by following the previously reported method[1].

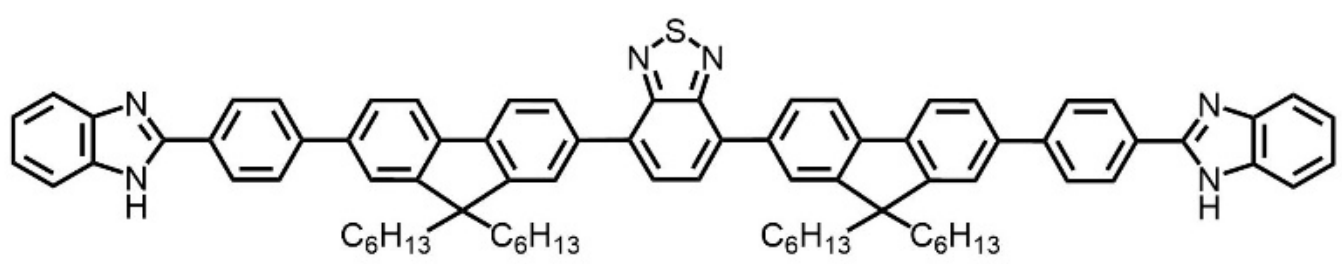

1

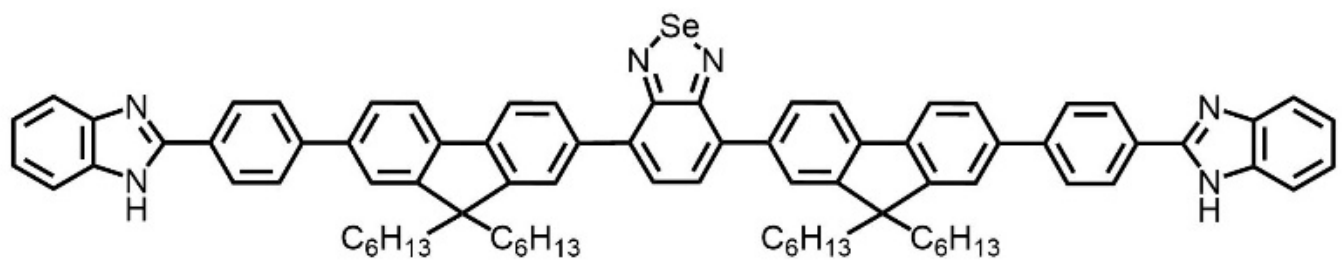

2

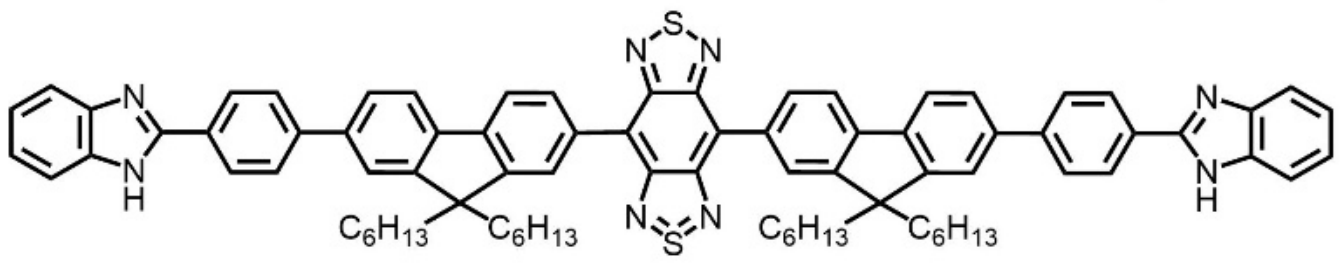

3

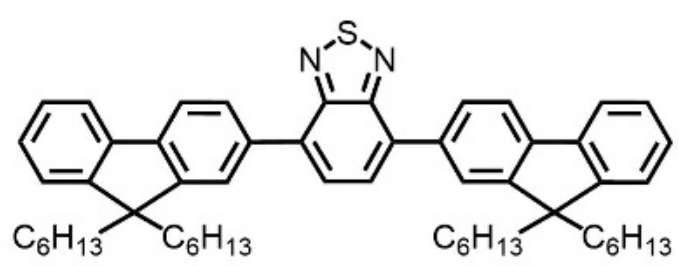

4

Supplementary Figure 1. Molecular structure of monomers. Molecular structures of **1** to **4**.

The synthesis of molecules can be briefly described as follows.

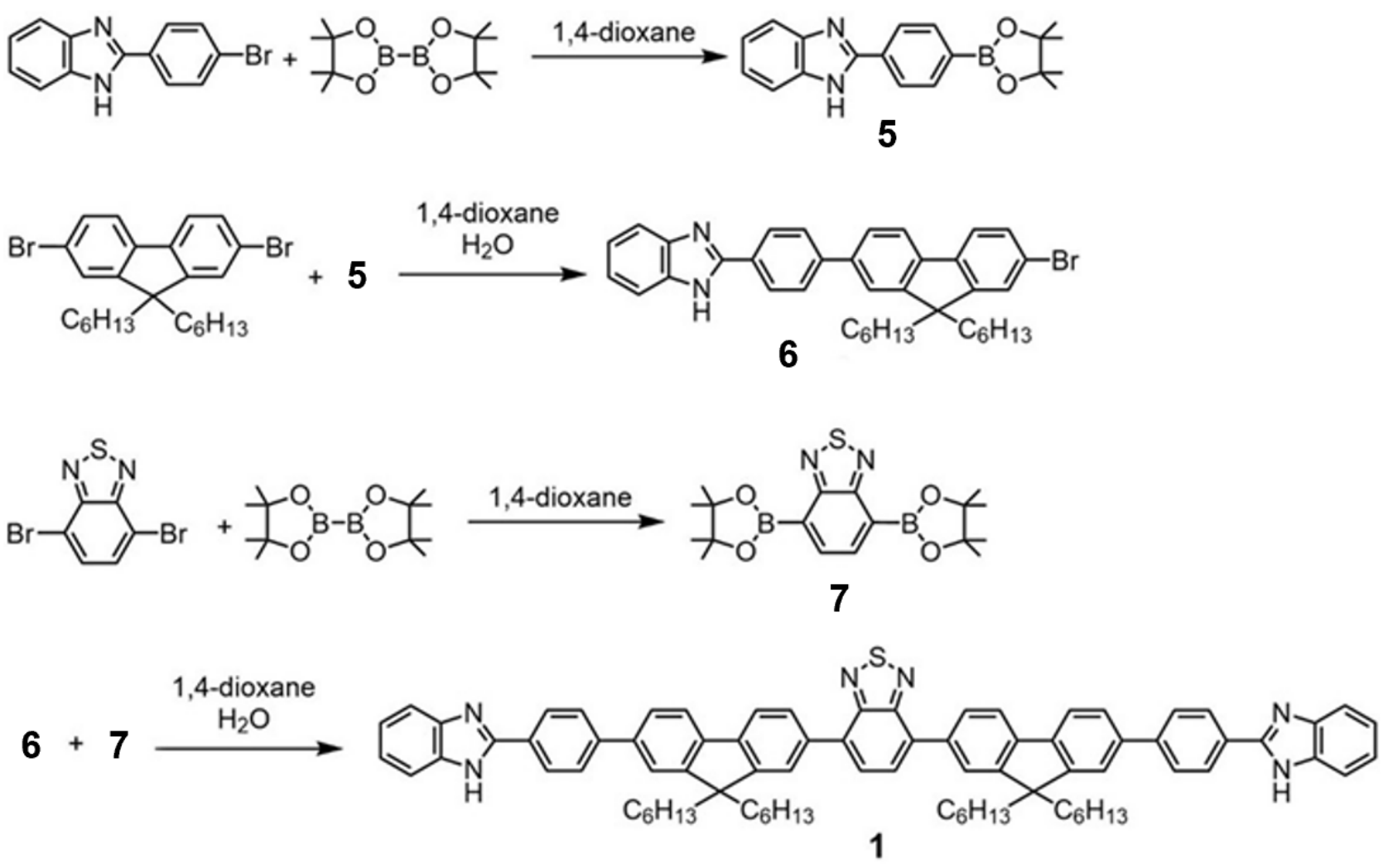

2-(4-(4,4,5,5-Tetramethyl-1,3,2-dioxaborolan-2-yl)phenyl)-1H-benzo[d]imidazole (**5**). A mixture of 2-(4-bromophenyl)benzimidazole, potassium acetate, bis(pinacolato)diboron, and Pd(dppf)Cl_2 in deoxygenated 1,4-dioxane was stirred at

80 °C under an argon atmosphere for 8 h. After removal of the solvent under reduced pressure, the residue was extracted with ethyl acetate and water. The combined organic layers were washed with saturated brine, dried over Na_2SO_4, and concentrated under reduced pressure. Purification by column chromatography (silica gel, petroleum ether/ethyl acetate = 30:1) afforded molecule **5**.

2-(4-(7-Bromo-9,9-dihexyl-9H-fluoren-2-yl)phenyl)-1H-benzo[d]imidazole (**6**). To a stirred solution of compound **5**, 2,7-dibromo-9,9-dihexyl-9H-fluorene, and $Pd(PPh_3)_4$ in deoxygenated 1,4-dioxane was added an aqueous K_2CO_3 solution. The reaction mixture was heated at 80 °C under argon for 8 h. After evaporation of the solvent under reduced pressure, the residue was poured into water and extracted with $CHCl_3$. The combined organic layers were washed with saturated brine, dried over Na_2SO_4, and concentrated under reduced pressure. The crude product was purified by column chromatography (silica gel, $CHCl_3$) to afford molecule **6**.

4,7-Bis(4,4,5,5-tetramethyl-1,3,2-dioxaborolan-2-yl)benzo[c][1,2,5]thiadiazole (**7**). A mixture of 4,7-dibromobenzo[c][1,2,5]thiadiazole, potassium acetate, bis(pinacolato)diboron, and $Pd(dppf)Cl_2$ in deoxygenated 1,4-dioxane (40 mL) was stirred at 80 °C under argon for 8 h. After removal of the solvent under reduced pressure, the residue was extracted with ethyl acetate and water. The combined organic layers were washed with saturated brine, dried over Na_2SO_4, and concentrated under reduced pressure. Purification by column chromatography (silica gel, petroleum ether/ethyl acetate = 20:1) afforded molecule **7**.

4,7-Bis(7-(4-(1H-benzo[d]imidazol-2-yl)phenyl)-9,9-dihexyl-9H-fluoren-2-yl)benzo[c][1,2,5]thiadiazole (**1**). To a stirred solution of molecule **6**, molecule **7**, and $Pd(PPh_3)_4$ in deoxygenated 1,4-dioxane (20 mL) was added an aqueous K_2CO_3 solution. The mixture was heated at 80 °C under argon for 8 h. After removal of the solvent under reduced pressure, the residue was poured into water and extracted with $CHCl_3$. The combined organic layers were washed with saturated brine, dried over Na_2SO_4, and concentrated under reduced pressure. The crude product was purified by column chromatography (silica gel, $CHCl_3$) to afford molecule **1**. The resulting molecule **1** was confirmed by 1H NMR and MALDI-MS.

Molecule **1**: ^1H NMR (400 MHz, THF-d8): δ: 11.83 (s, 2H), 8.32, 8.30 (d, J = 8.0 Hz, 4H), 8.25 (s, 2H), 8.15, 8.13 (d, J = 8.0 Hz, 2H), 8.06 (s, 2H), 8.00-7.92 (m, 8H), 7.87 (s, 2H), 7.82, 7.80 (d, J = 8.0 Hz, 2H), 7.73, 7.72, 7.70 (m, J = 4.0 Hz, 2H), 7.50, 7.49, 7.47 (m, J = 4.0 Hz, 2H), 7.21, 7.20, 7.19 (m, J = 4.0 Hz, 4H), 2.27-2.22 (q, J = 2.0 Hz, 8H), 1.20-1.17 (m, 24H), 0.91-0.89 (m, 8H), 0.83, 0.82, 0.81 (m, J = 4.0 Hz, 12H). ^{13}C NMR (175 MHz, $CDCl_3/CD_3OD$ = 3:1, ppm): δ: 159.8, 152.1, 151.2, 151.1, 143.9, 140.7, 140.5, 138.7, 137.0, 135.2, 128.4, 128.3, 127.6, 127.1, 126.8, 126.0, 124.1, 123.3,

121.2, 120.2, 119.5, 114.4, 55.2, 40.1, 31.2, 29.4, 23.7, 22.3, 13.5. MALDI-MS: (*m/z*) =1185.7.

Molecules **2**-**4** were synthesized according to modified procedures based on the method used for molecule **1** and previously reported protocols[1].

Molecule **2**: ^{1}H NMR (300 MHz, THF-d8): δ: 11.83 (s, 2H), 8.32, 8.30 (d, J = 8.0 Hz, 4H), 8.25 (s, 2H), 8.15, 8.13 (d, J = 8.0 Hz, 2H), 8.06 (s, 2H), 8.00-7.92 (m, 8H), 7.87 (s, 2H), 7.82, 7.80 (d, J = 8.0 Hz, 2H), 7.73, 7.72, 7.70 (m, J = 4.0 Hz, 2H), 7.50, 7.49, 7.47 (m, J = 4.0 Hz, 2H), 7.21, 7.20, 7.19 (m, J = 4.0 Hz, 4H), 2.27-2.22 (q, J = 2.0 Hz, 8H), 1.20-1.17 (m, 24H), 0.91-0.89 (m, 8H), 0.83, 0.82, 0.81 (m, J = 4.0 Hz, 12H). ^{13}C NMR (175 MHz, $CDCl_3/CD_3OD$ = 3:1, ppm): δ: 163.9, 156.1, 155.2, 147.9, 144.8, 140.7, 140.5, 138.7, 137.0, 135.2, 132.6, 132.4, 131.7, 131.2, 130.9, 130.1, 128.2, 127.4, 125.3, 124.3, 123.6, 118.5, 59.3, 44.2, 35.3, 33.5, 27.8, 26.4, 17.6. MALDI-MS: (*m/z*) = 1232.2.

Molecule **3**: ^{1}H NMR (400 MHz, Chloroform-*d*, ppm) δ 11.8 (s, 2 H), 8.33 (d, J = 8.0 Hz, 4 H), 8.16 (d, J = 8.0 Hz, 2 H), 8.08 (s, 2 H), 8.02-7.94 (m, 8 H), 7.89 (s, 2 H), 7.83 (d, J = 8.0 Hz, 2 H), 7.73 (d, J = 8.0 Hz, 2 H), 7.51 (d, J = 8.0 Hz, 2 H), 7.22 (d, J = 8.0 Hz, 2 H), 2.51-2.24 (m, 8 H), 1.35-1.14 (m, 24 H), 0.96-0.93 (m, 8 H), 0.84 (t, J = 8.0 Hz, 12 H). MALDI-MS: (m/z) =1243.4.

Molecule **4**: ^{1}H NMR (400 MHz, THF-d8, ppm): δ: 8.16 (s, 2 H), 8.06 (d, J = 8.0 Hz, 2 H), 7.98 (s, 2 H), 7.88 (d, J = 8.0 Hz, 2 H), 7.80 (d, J = 8.0 Hz, 2 H), 7.42 (d, J = 8.0 Hz, 2 H), 7.29-7.35 (m, 4 H). MALDI-MS: (m/z) =800.380.

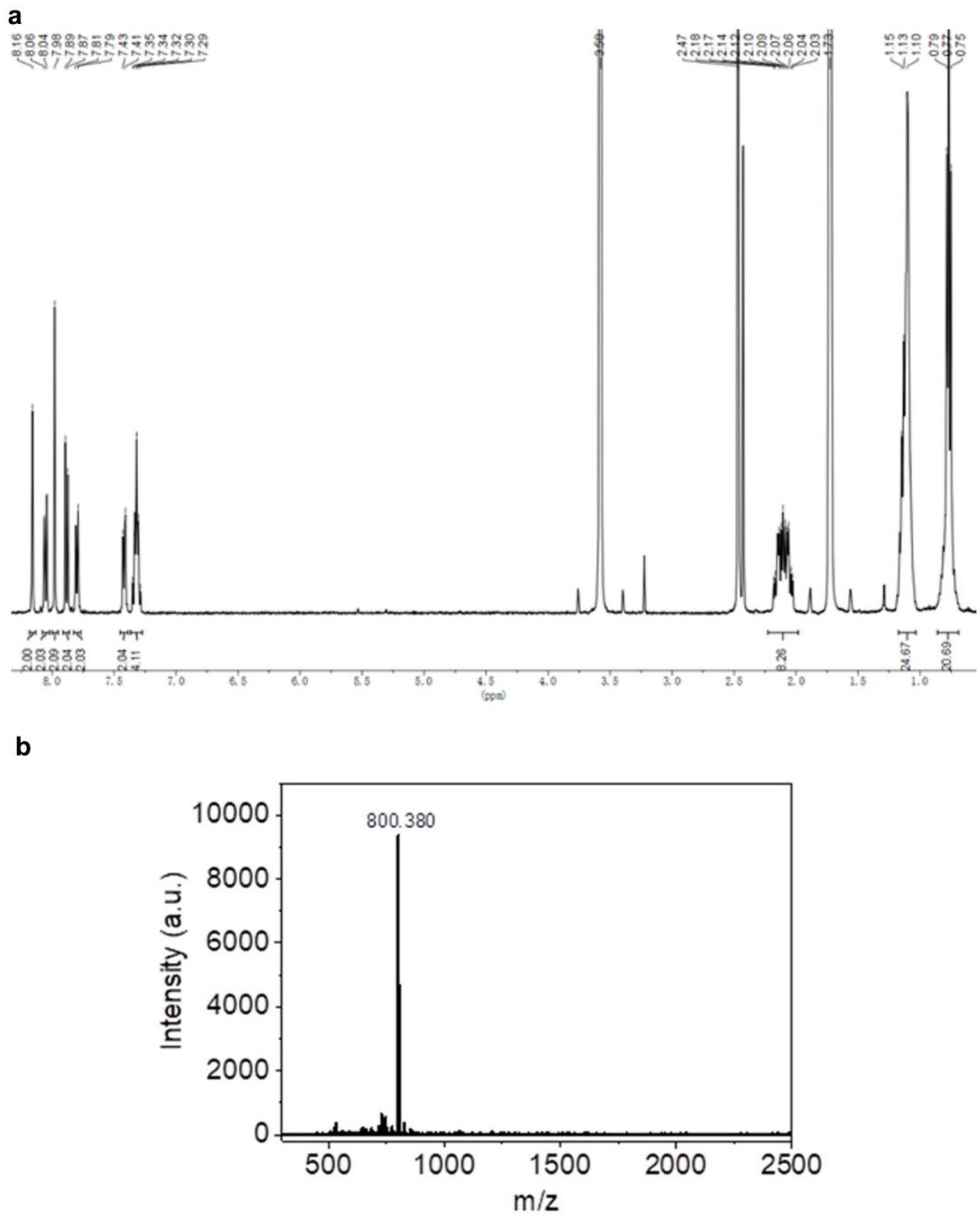

Supplementary Figure 2. Characterization of molecule 4. (**a**) ^{1}H NMR spectrum of molecule **4**. (**b**) MALDI-MS spectrum of molecule **4**.

Preparation methods

Preparation of large area single-crystalline film via a dual solid-liquid interface-confined strategy

Fabrication of asymmetric-wettability topographical template.

Five-inch N-doped silicon wafers (<100> oriented) were used to fabricate asymmetric topographical templates with a thickness of 525 μm. The silicon wafers were coated with photoresist (Shipley Microposit S1800 series) for about ~1 μm, so as to transfer the pre-designed patterns onto the surfaces by employing a direct laser writing apparatus (Heidelberg DWL200). After irradiation and development, the deep reactive-ion etching (DRIE, Alcatel 601E) with fluorine-based reagents was performed for 6 mins to fabricate the periodic micropillar structures. After resist stripping (Microposit Remover 1165), the topographical template was generated and subsequently cleaned with ethanol, acetone, and isopropyl alcohol. To fabricate asymmetric-wettability micropillars, selective modification was then performed by exposing unprotected sidewalls to heptadecafluorodecyltrimethoxysilane (FAS), while the tops of micropillars were protected by pre-covered SU8. After removing SU8 from the tops by using acetone, asymmetric-wettability topographical template with hydrophilic tops and hydrophobic sidewalls were formed.

Fabrication of single-crystalline film photoresists.

The initial saturated solution of molecular **1** were prepared by dissolving synthesized powder in pure propanol. Then the precursor solution was obtained at a volume ratio of 1:2 by adding dichloroethane into the initial solution. Silicon wafers were cut into 1.5×1.5 cm pieces and wiped clean with alcohol as substrate. In typical assemble system, The precursor solution (1 mg mL^{-1}) was dropped on an asymmetric-wettability micropillar template, following by covering with a pre-cleaned substrate to form the dual solid-liquid two-phase interface-confined space. After applying constant pressure to the composed sandwich structure unit, the whole unit was heated at a constant temperature of 60°C in a drying oven to evaporate the solvent. Finally, the patterned crystal film was left on the substrate for subsequent characterization.

Thickness regulation of large area single-crystalline film photoresists with tuneable dimensions.

To precisely tune the thickness of the crystal film, we employed a synergistic strategy of concentration and pressure. Multiple concentrations of 0.2-1.5 mg mL^{-1} were obtained by diluting the original solution, preserving the volume ratio of dichloroethane to propanol in total solvent as 1:2. Therefore, crystalline films in thickness of 236 to 2449 nm were fabricated with various concentrations of 0.1-1.5 mg mL^{-1}. To further

regulate the thickness of the film, the pressure ranging from 6.1 to 15.9 MPa were applied using different-concentration solutions.

Preparation of bulk crystals for single crystal analysis.

Bulk crystals from **1** with over 100 μm in each dimension for single crystal analysis and 3D topochemical polymerization-based photocarving were prepared by carefully adding hexane (3.5 mL) on the top of the propanol solution of **1** (0.5 mL, 0.5 mg/mL) in a vial (10 mL) to allow the slow crystal growth of **1** at the solvent interface. The same packing method from single bulk crystal and large-area single-crystalline film photoresist from **1** is confirmed by the X-ray diffraction (PXRD) results (attached figure).

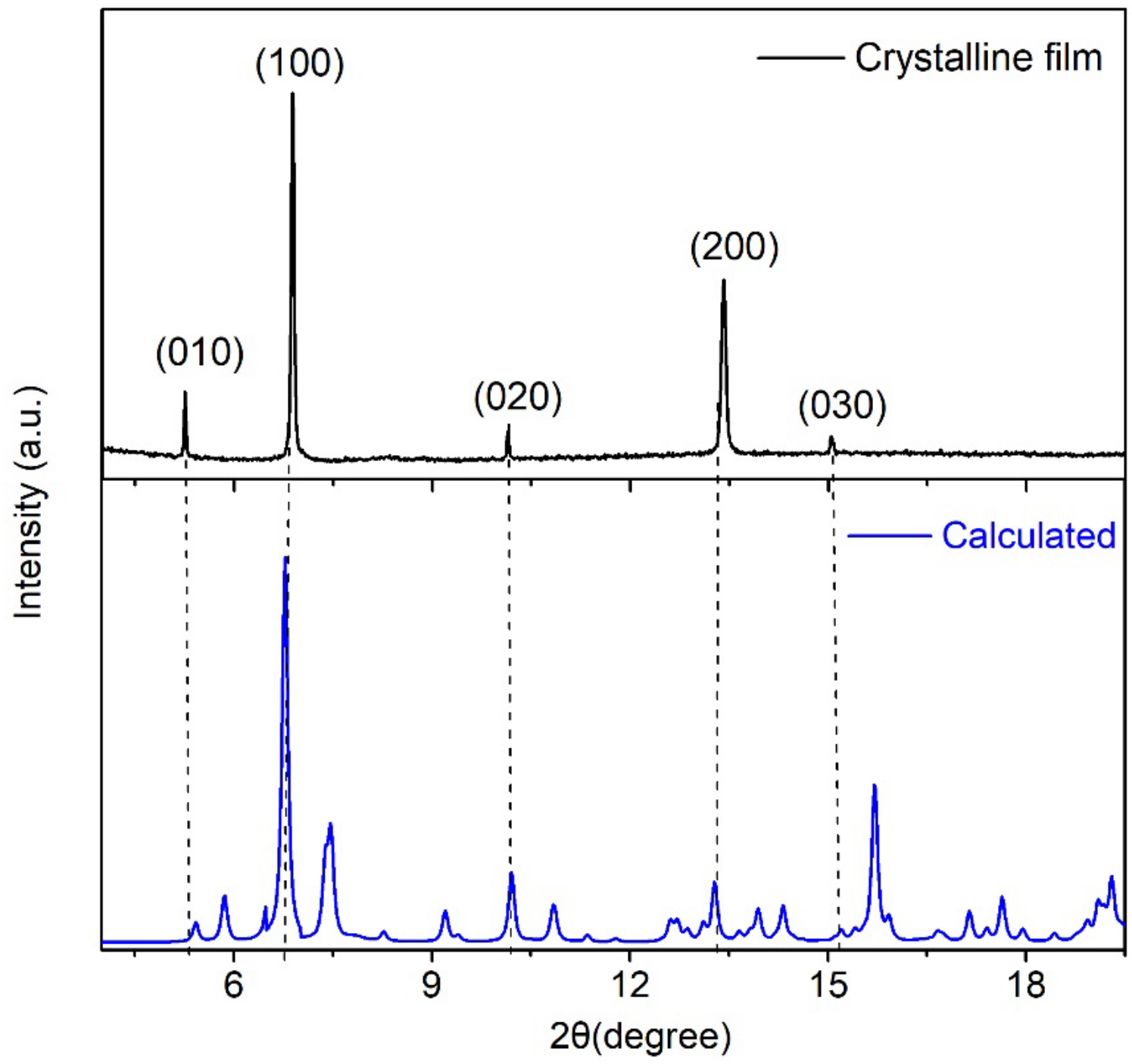

Supplementary Figure 3. Comparison of X-ray diffraction (XRD) patterns. a, Experimental XRD pattern (black) of large-area single-crystalline film photoresists from **1.** and **b**, Calculated XRD patterns (blue) from single-crystal XRD structure of bulk crystal from **1**.

Topochemical polymerization-based 2D photocarving of single-crystalline film photoresists

The programmed photocarving setup is a home-built system based on the home-build spatiotemporal light selective excitation system (SLSES). Upon utilizing a computer-controlled acousto-optic deflector (AOD) system to adjust the position and intensity of the optical traps (trap position resolution is 0.01 nm). Laser beams (containing cw lasers with wavelengths of 405, 470, 515, 635, and 1064 nm and pulsed laser with wavelength of 980 nm) is captured through AOD and beam expander, tightly focused onto the target sample by an objective lens (×100, numerical aperture: 1.4, WD = 2.0, oil-immersion, Nikon). In the photocarving experiments, optical trap sequences of different patterns such as "wire", "circle", "pentagon", and "horse" patterns are respectively set up with program designed trap points. The laser scanning dwell times is 16 ms for each trap point, with laser power set range from 1 to 700 μW. Upon laser activation, the laser beam rapidly scans through the designated patterned optical trap sequence point by point, achieving complete programmed photocarving patterns in 50 to 200 s for each cycle. The experimental process can be captured by a high-speed CCD (IDS) camera and monitored in real-time.

Preparation of positive mode photocarved patterns on glass substrate

The large-area single-crystalline film photoresists on silicon substrate after photocarving was first placed in a glass-bottomed culture dish (model: BS-20-GJM, Biosharp life science). Put the glass-bottom culture dish containing the photocarved samples on an upright optical microscope (Olympus IX71). Add 5 mL of acetonitrile and dimethyl sulfoxide (DMSO) solvent mixture in a volume ratio of 3:1 into the glass-bottom culture dish. Real-time observation through the optical microscope reveals that the unexposed areas remain intact while the exposed areas dissolve completely, yielding positive photolithographic patterns. To obtain detailed morphology of the positive photocarving patterns, SEM (HITACHI SU8010) were used for the characterization.

Preparation of negative mode photocarved patterns on silicon substrate

The large-area single-crystalline film photoresists on silicon substrate after photocarving was first placed in a glass-bottomed culture dish (model: BS-20-GJM, Biosharp life science). Position the glass-bottom culture dish containing the sample under an upright optical microscope (Olympus IX71). Rinse the glass-bottom culture dish containing the sample with 5mL of a developer consisting of chloroform, methanol, and n-hexane with volume ratio of 1:3:20 (v/v/v), followed by thorough immersion and cleaning with hexane. Real-time observation through the optical microscope reveals that the exposed areas remain intact while the unexposed areas dissolve completely, yielding negative photolithographic patterns. To obtain detailed morphology of the

negative photocarving patterns, SEM (HITACHI SU8010) were used for the characterization.

Preparation of negative mode photocarved 3D structures

The dodecahedral 3D structures were printed using a 1030 nm femtosecond laser with a pulse width of approximately 400 fs, a repetition rate of 0.1 MHz (YbFemto-ProH), and an optical power of 0.5 mW (pulse energy: 5 nJ). The laser beam was expanded using a 10× beam expander and then focused with a 63× oil-immersion objective (Nikon, numerical aperture: 1.4) in an inverted optical setup. To enable the 3D printing, a 2D galvanometric scanner was coordinated with a piezo nanopositioner (P-611.Z Piezo Z stage). The scanning speed ranged from 2 to 20 μm/s for the printed patterns. By steering the laser spot location, the programmed 3D patterns were printed into a large-area single-crystalline film placed on the surface of a glass coverslip (~150 μm thickness). Negative-mode 3D photocarving structures were obtained by rinsing the printed samples in a glass-bottom culture dish with 5 mL of a developer consisting of chloroform, methanol, and n-hexane with volume ratio of 1:3:20 (v/v/v) for three cycles, followed by thorough immersion and cleaning with hexane. The morphology of the resulting negative-mode dodecahedral 3D structure patterns was imaged by SEM (HITACHI SU8010).

Quantitative analysis of LER in the fabricated photocarved structures

The line edge roughness (LER) of the fabricated micro/nanostructure edges was analyzed using ProSEM V3.0. After inputting the scale bar information, a region of interest (ROI) was selected for edge detection to obtain the roughness data. The feature type was set to *Line and Spaces*, the polarity to *Line/Pillar*, the position to *mid*, and the analysis method to *sigmoidal signal*.

Simulation details for the light triggered topological polymerization in single-crystalline film photoresists

We consider an n × n 2D triangular lattice of monomers in a crystalline film. The bonding probability p between two nodes residing on adjacent lattice sites is set to be in the range [0, 1], which is linear increased with laser power (Supplementary Figure 21). Due to the limitations to polymerization on the photoresists lattice imposed by the molecular orientation, we set rules for bonding formation in the crystalline film and implement a Monte Carlo type of simulation in MATLAB. Specifically, we chose the molecular orientation of monomers to be the parallel direction, and each node has one reaction site on the left and one on the right in the parallel direction. We then proceed to calculate the probability P(m) of connecting any two nodes in the triangular lattice through m nodes.

$$P(m) = \sum_{i,j} P(i,j,m)$$

Where i and j enumerate all n by n nodes on the triangular lattice, and $P(i,j,m)$ is the probability of finding two nodes connected in the same chain and separated by distance m.

When new bonds form, the lattice sites involved are colored. Each connected chain is represented by a different color.

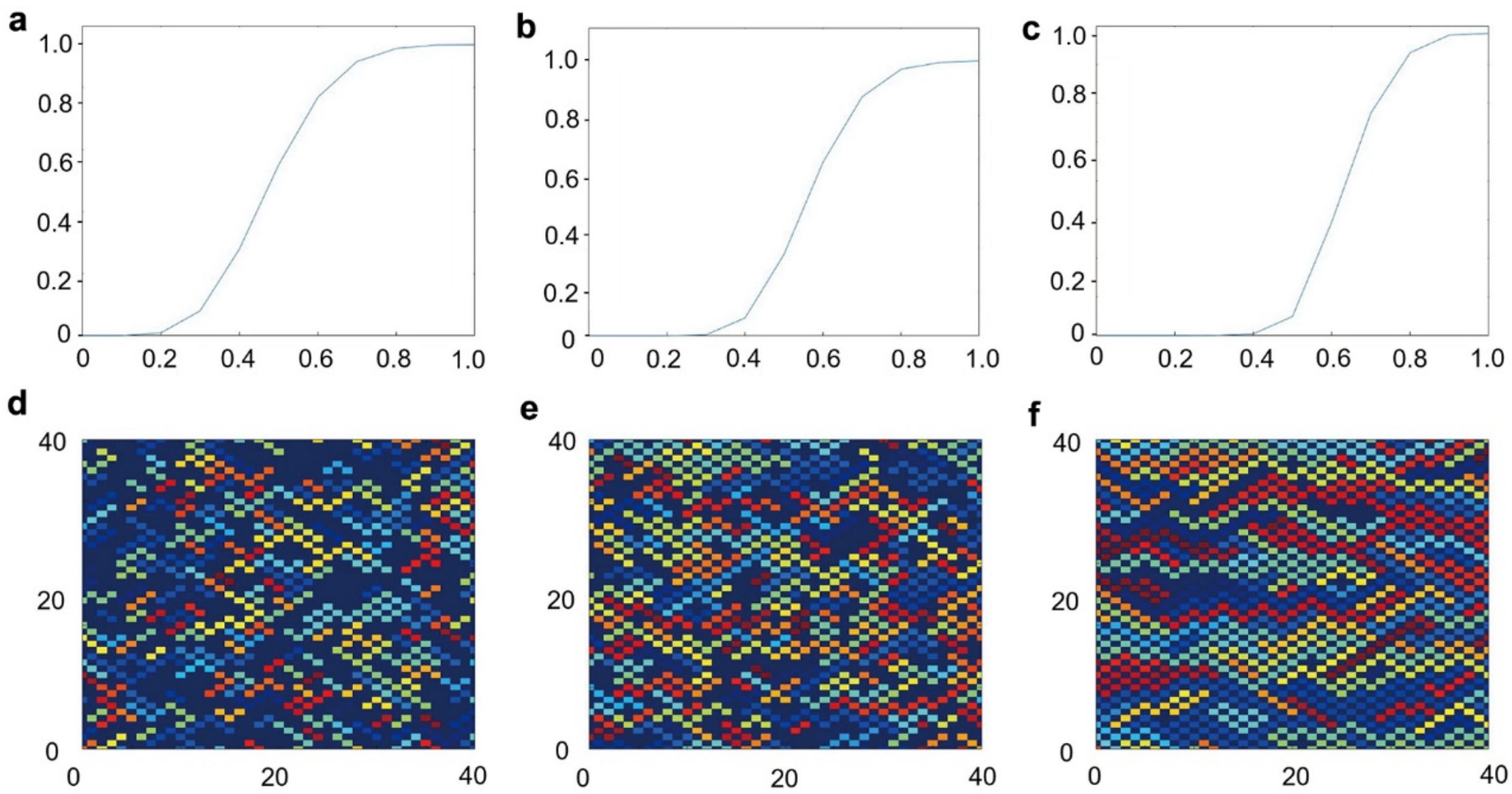

Supplementary Figure 4. The simulated probability of polymer formation with certain degree of polymerization (D_p) with varied relative reaction kinetics in a crystalline matrix. The calculated probability of formed polymer (**a**, D_p = 5, **b**, D_p = 10, and **c**, D_p = 20) as a function of relative reaction kinetics in a crystalline matrix. The

corresponding schematic diagram of polymer formation with different Dp in a 500 × 500 matrix (**d**, D_p = 5, **e**, D_p = 10, and **f**, D_p = 20).

Other supplementary figures

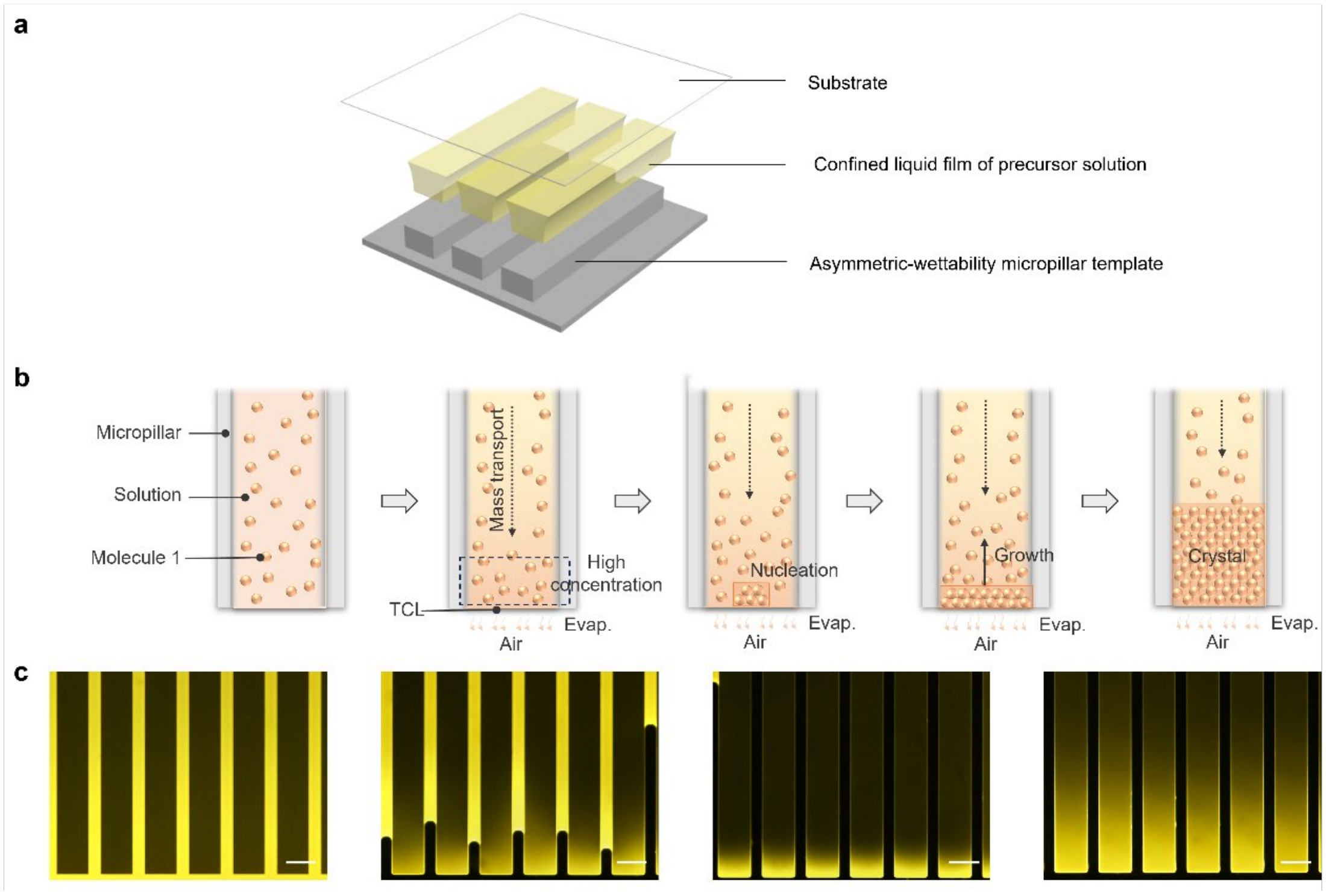

Supplementary Figure 5. The dewetting progress in a Sandwich-type structure using the confined capillary-bridge assembly method. a, The large-area highly oriented crystalline films were constructed through a "Sandwich-type" confined capillary-bridge assembly technique, which involves bridging a solution over asymmetric-wettability micropillar templates on various pre-cleaned substrates such as silicon, glass, and quartz. **b**, Schematic diagram illustrating concentration distribution changes during dual solid-liquid interface-confined assembly process. **c**, *In-situ* fluorescence microscope images of the dewetting process. Scale bar = 100 μm.

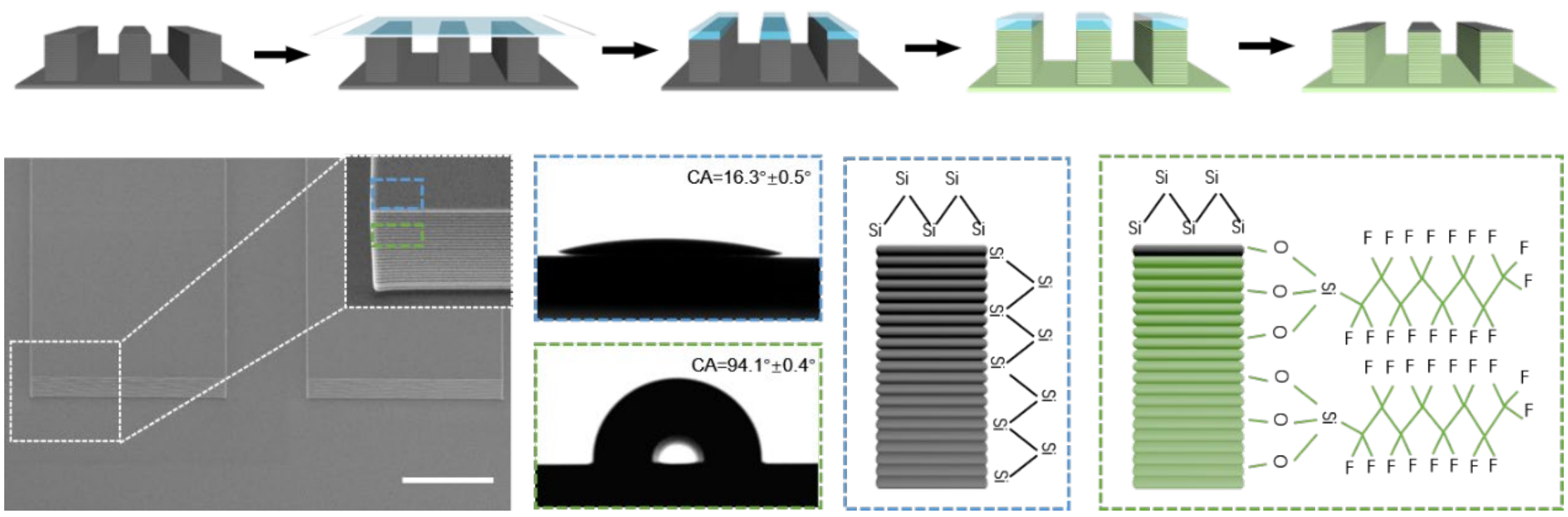

Supplementary Figure 6. Asymmetric-wettability micropillar templates.

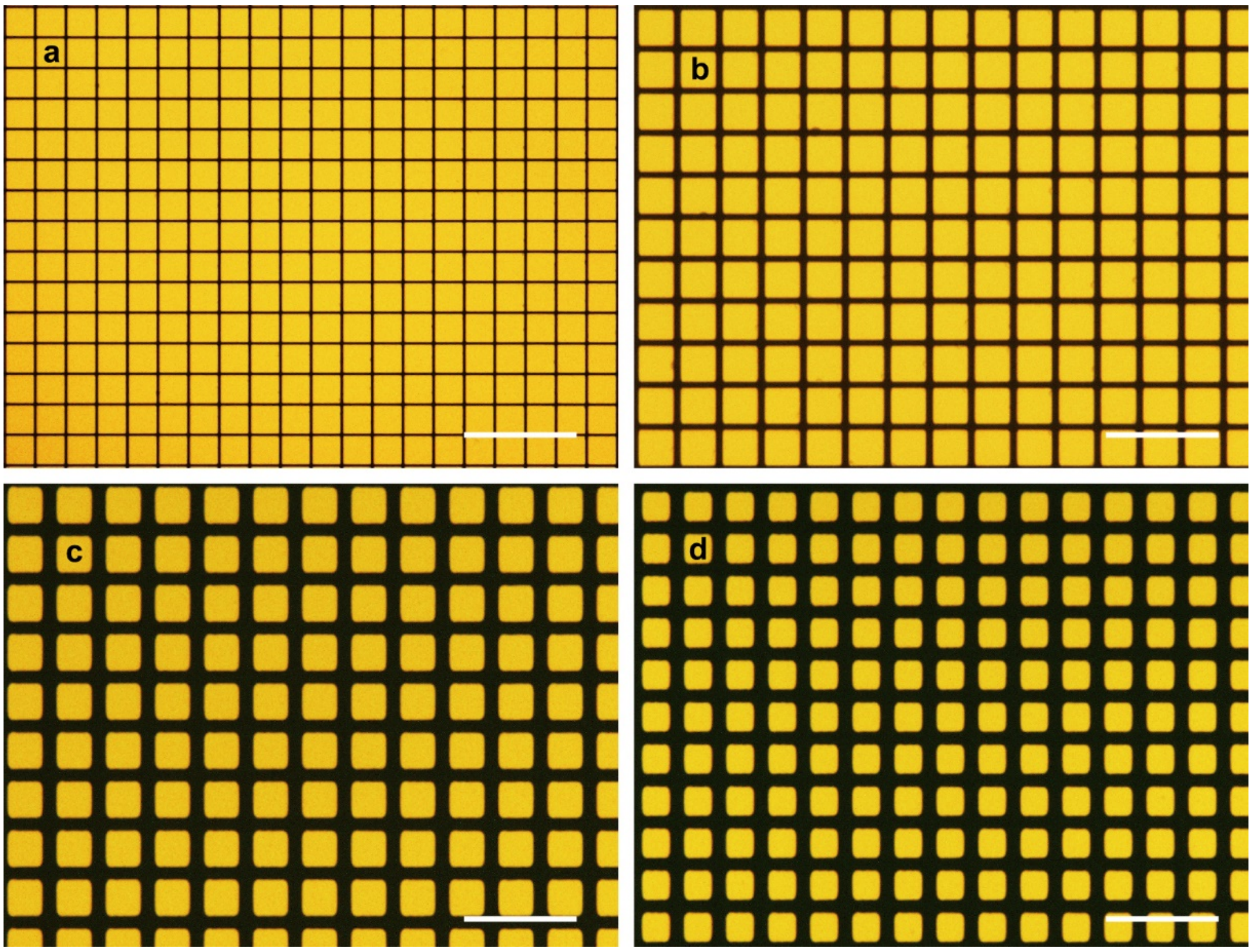

Supplementary Figure 7. Large-area single-crystalline patchy film arrays from 1. Fluorescence optical microscopy images of the large-area single-crystalline film arrays with varying gap distances **a**, 15 μm, **b**, 30 μm, **c**, 45 μm, and **d**, 60 μm. Scale bar = 1 mm.

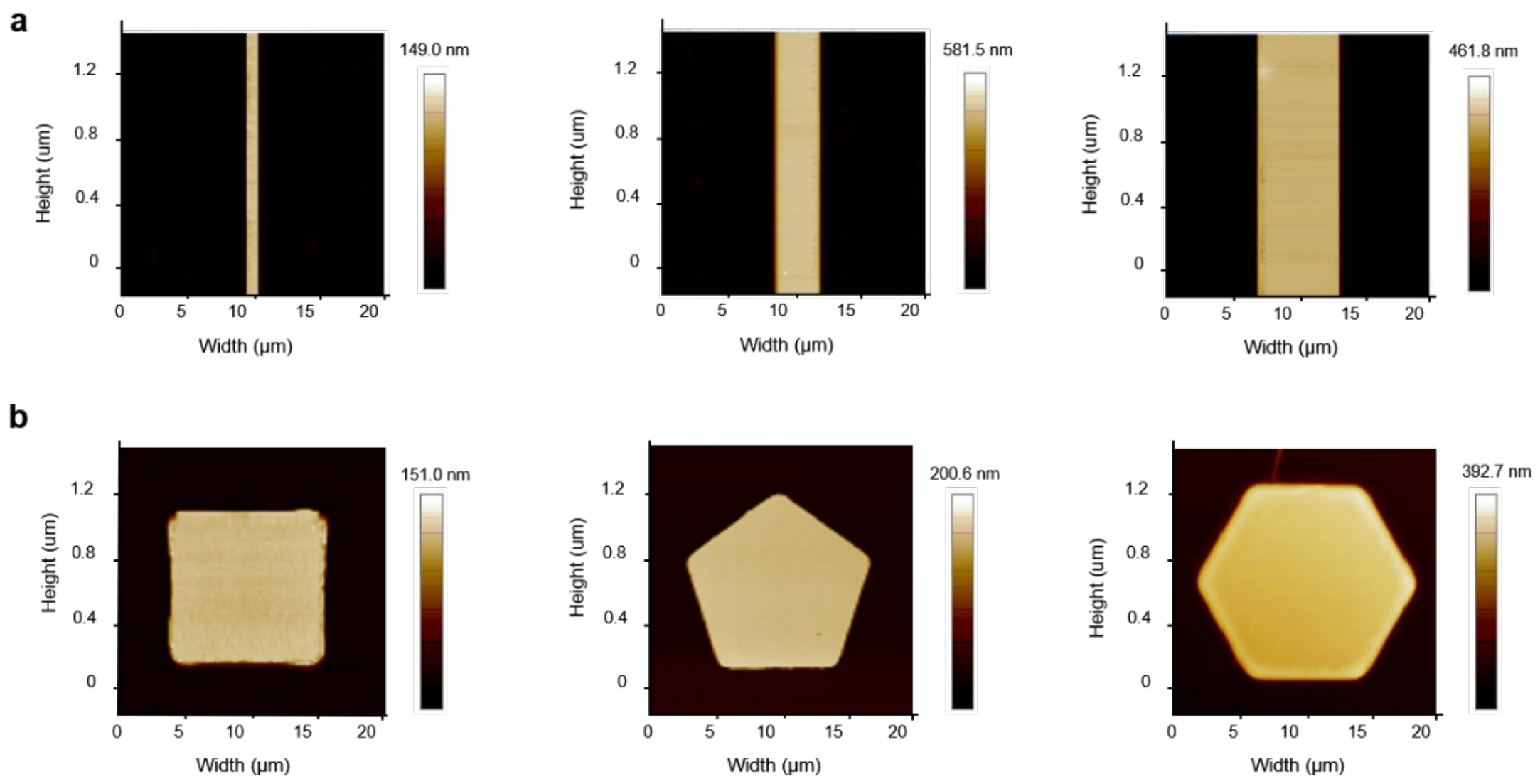

Supplementary Figure 8. Shapes and sizes control of mono-patchy single-crystalline film photoresists from 1. AFM of polygonal shape of self-assembly patterning. **a**, threads with tuneable width. **b**, polygons include squares, pentagons, and hexagons.

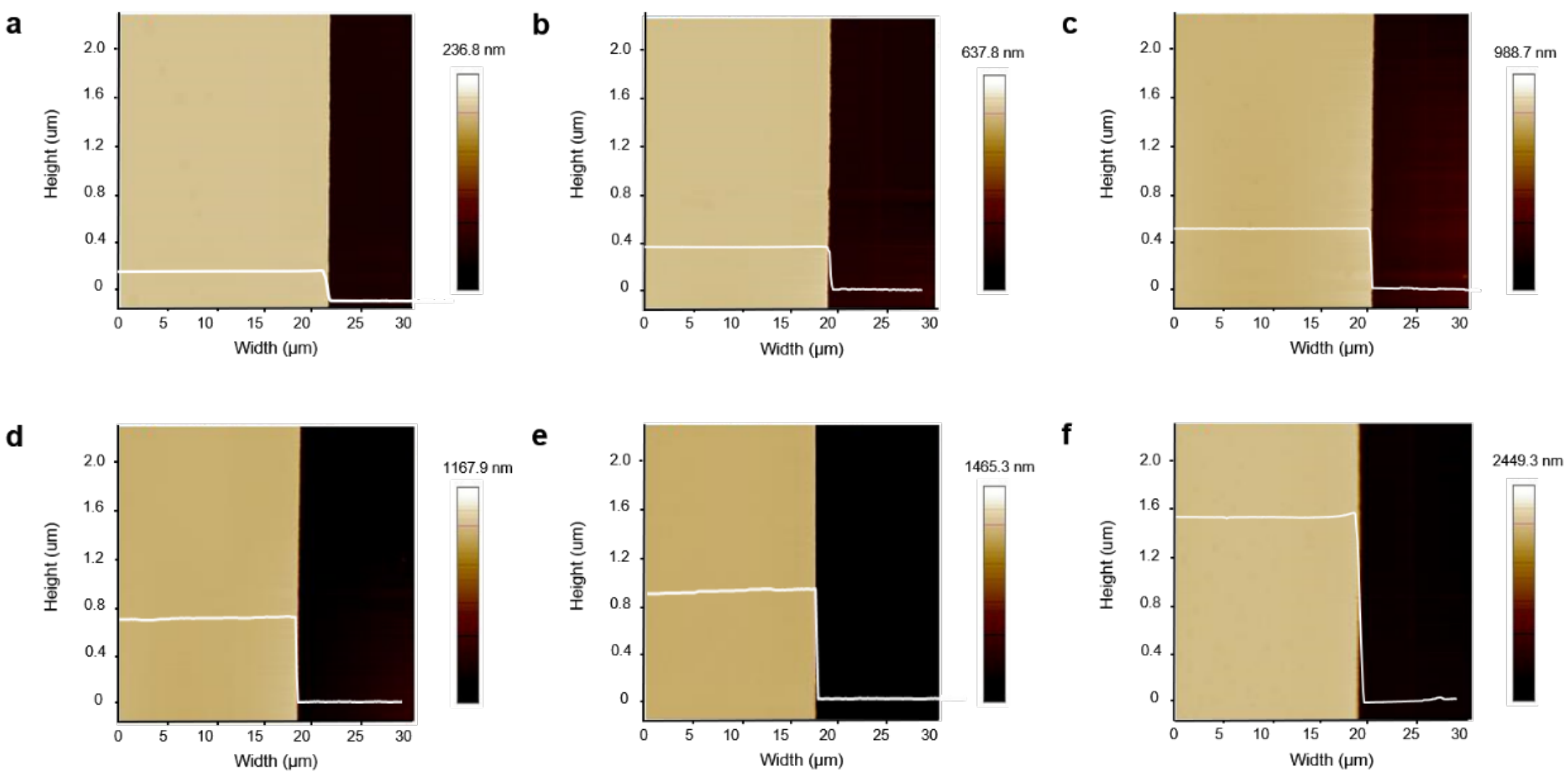

Supplementary Figure 9. Precise thickness modulation of mono-patchy single-crystalline films from 1. a-f, AFM characterization of single-crystalline film photoresist indicates the thickness ranging from 239 to 2449 nm controlled by using different concentration of solution.

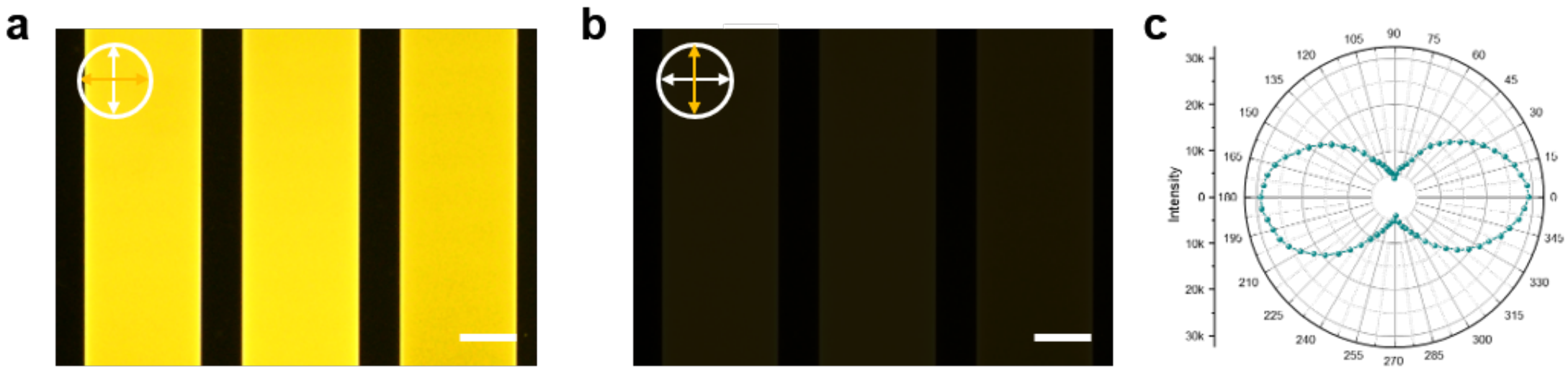

Supplementary Figure 10. Polarization property of single-crystalline film photoresist from 1. Fluorescence images of the portion of typical single-crystalline film photoresist array from **1** imaged at polarized angle at **a**, 90° and **b**, 30°. Scale bar = 50 µm. **c**, The polarized emission of the single-crystalline film photoresist as a function of polarized angles.

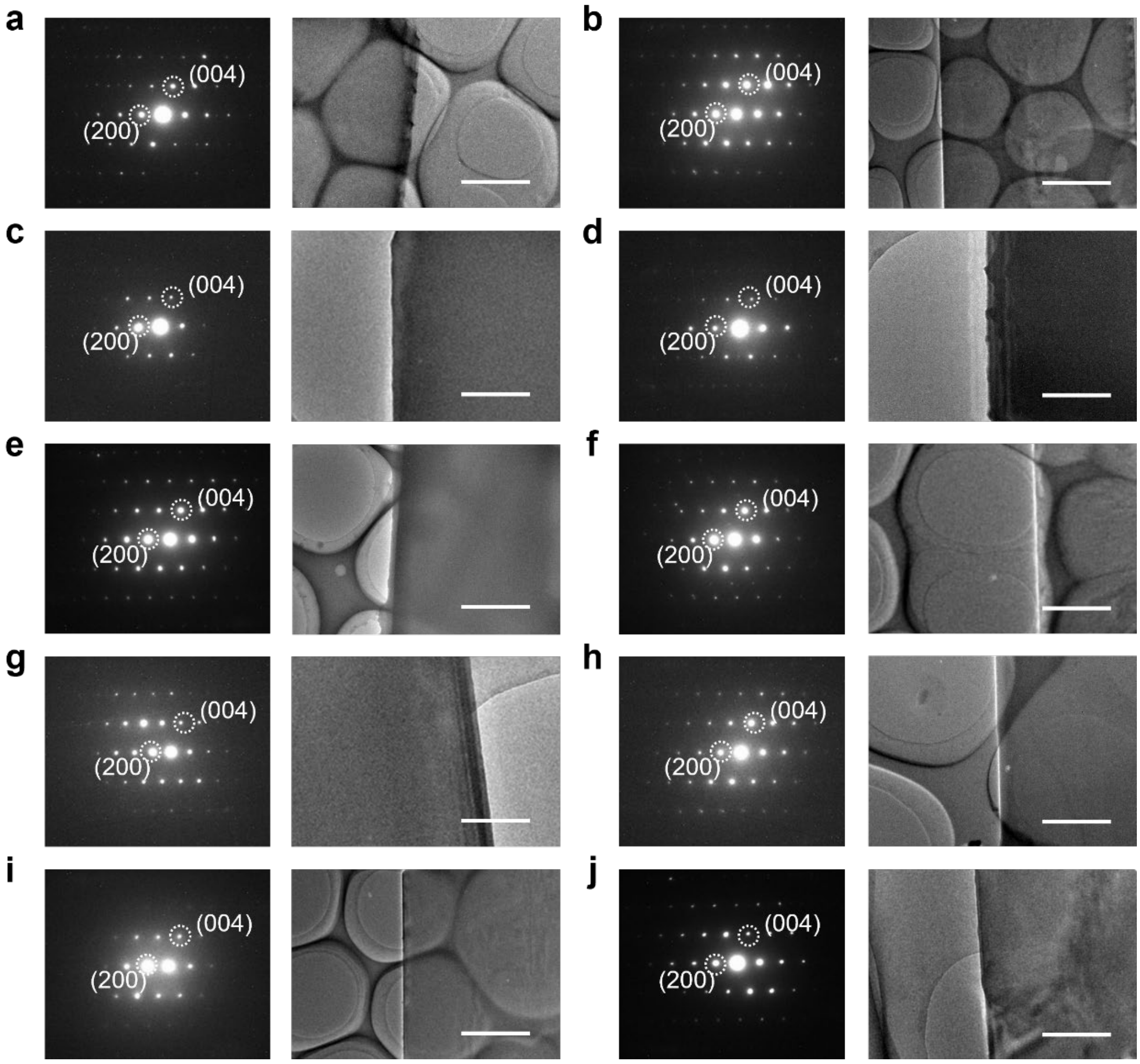

Supplementary Figure 11. Uniform crystallographic orientation revealed by TEM and SAED. TEM images obtained from randomly selected regions of the film, along with the corresponding SAED patterns, indicate a high degree of crystallographic orientation uniformity within individual domains, without observable grain boundaries associated with polycrystalline structures. Scale bar = 1 μm. The circular patches beneath the single-crystalline film of **1** in (**a-j**) originate from the supporting carbon film on the TEM copper grid.

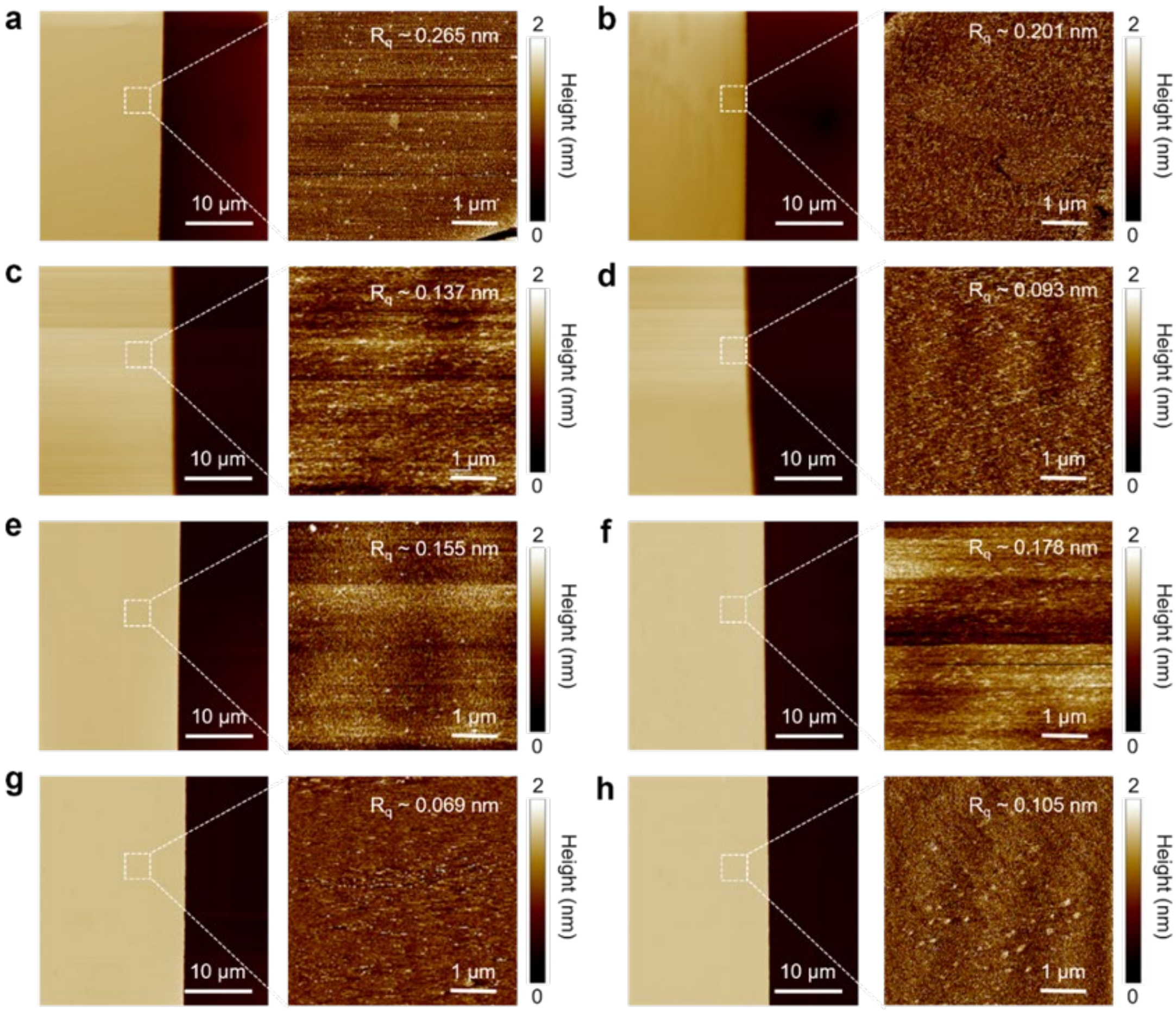

Supplementary Figure 12. **Continuous morphology and surface flatness revealed by AFM.** High-resolution AFM images obtained from multiple regions of the film reveal a continuous morphology without discernible cracks or inter-platelet boundaries over lateral length scales of ~20 μm. The surface roughness measured across ten randomly selected areas (~25 μm^2 each) falls within 0.05-0.27 nm, indicating a high degree of surface flatness.

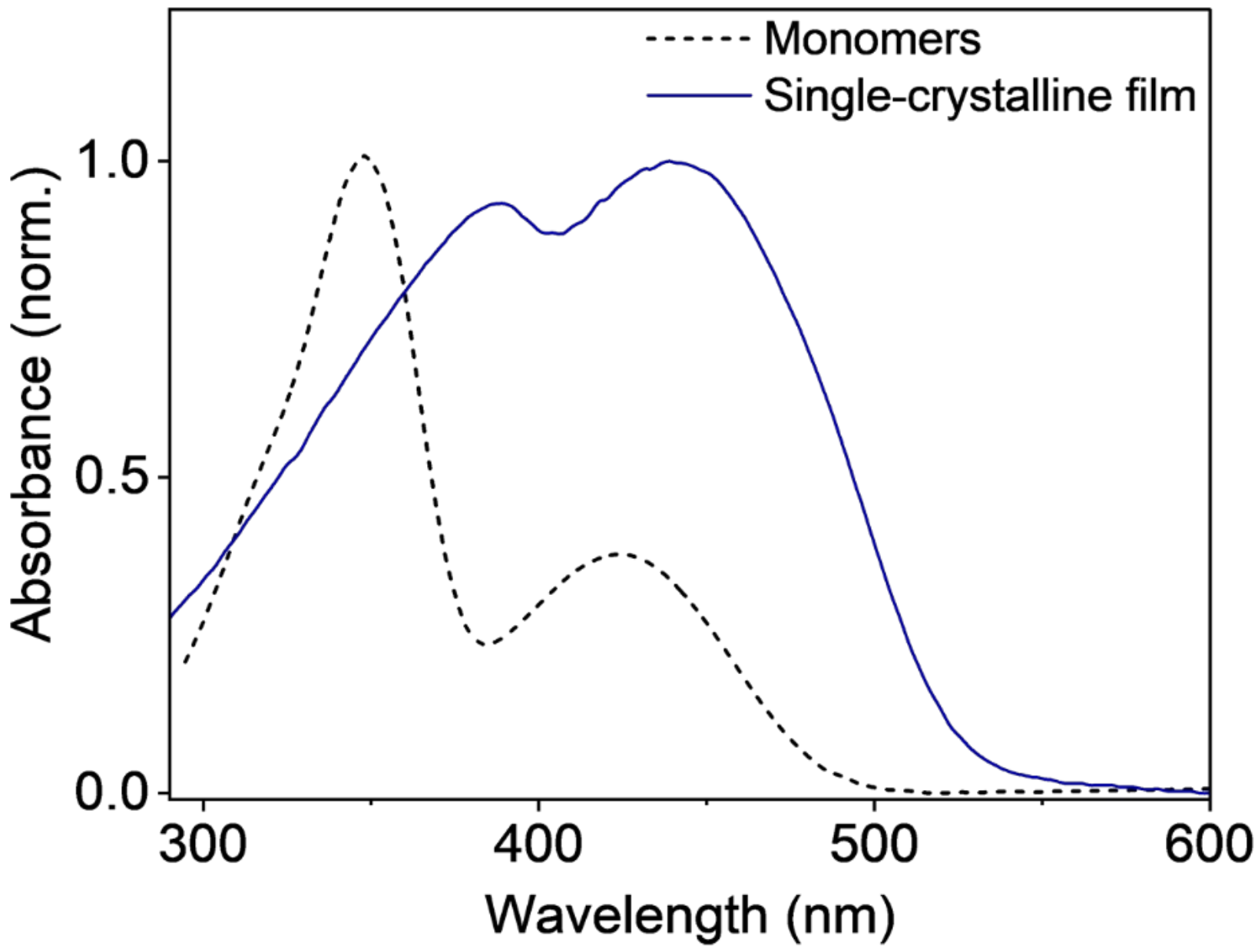

Supplementary Figure 13. UV-vis absorption of monomer solution in THF (black dashed line) and single-crystalline film (blue solid line) from **1**.

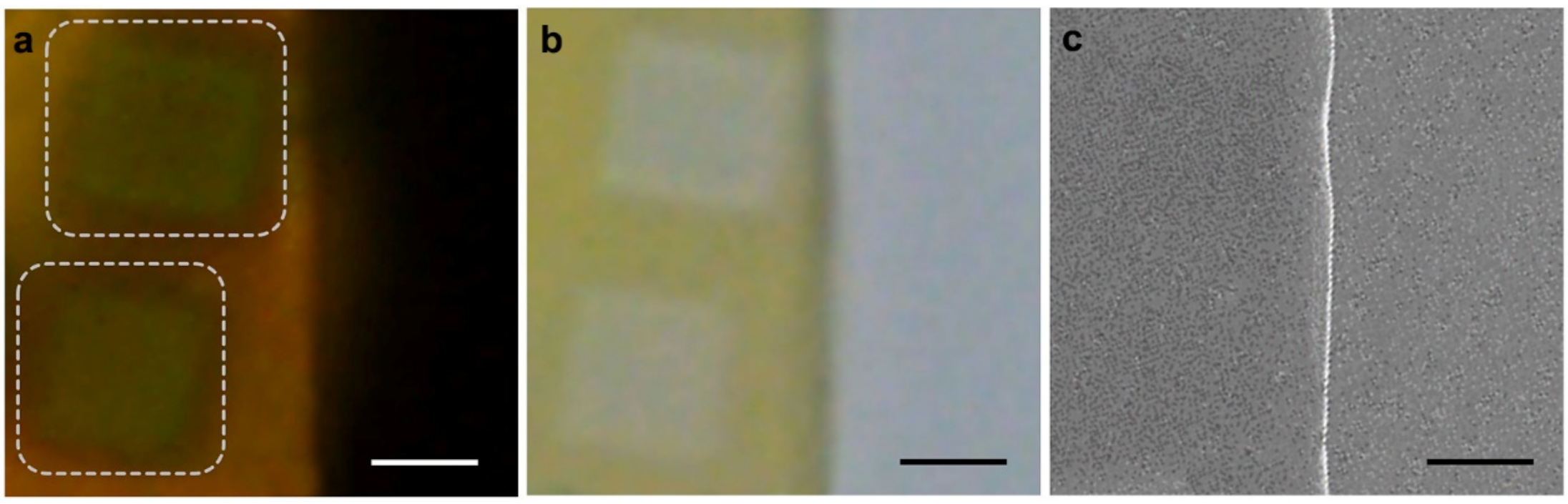

Supplementary Figure 14 Topochemical polymerization-based photocarving on single-crystalline film photoresist. a, Fluorescence mode and **b**, Transmission mode optical image and, **c**, SEM image of the photocarved single-crystalline film photoresist from **1** using continuous wavelength 488 nm visible lasers (470 nm, laser power 10 μW). Grey dotted squares represent the visible light exposed regions. Scale bar = 2 μm.

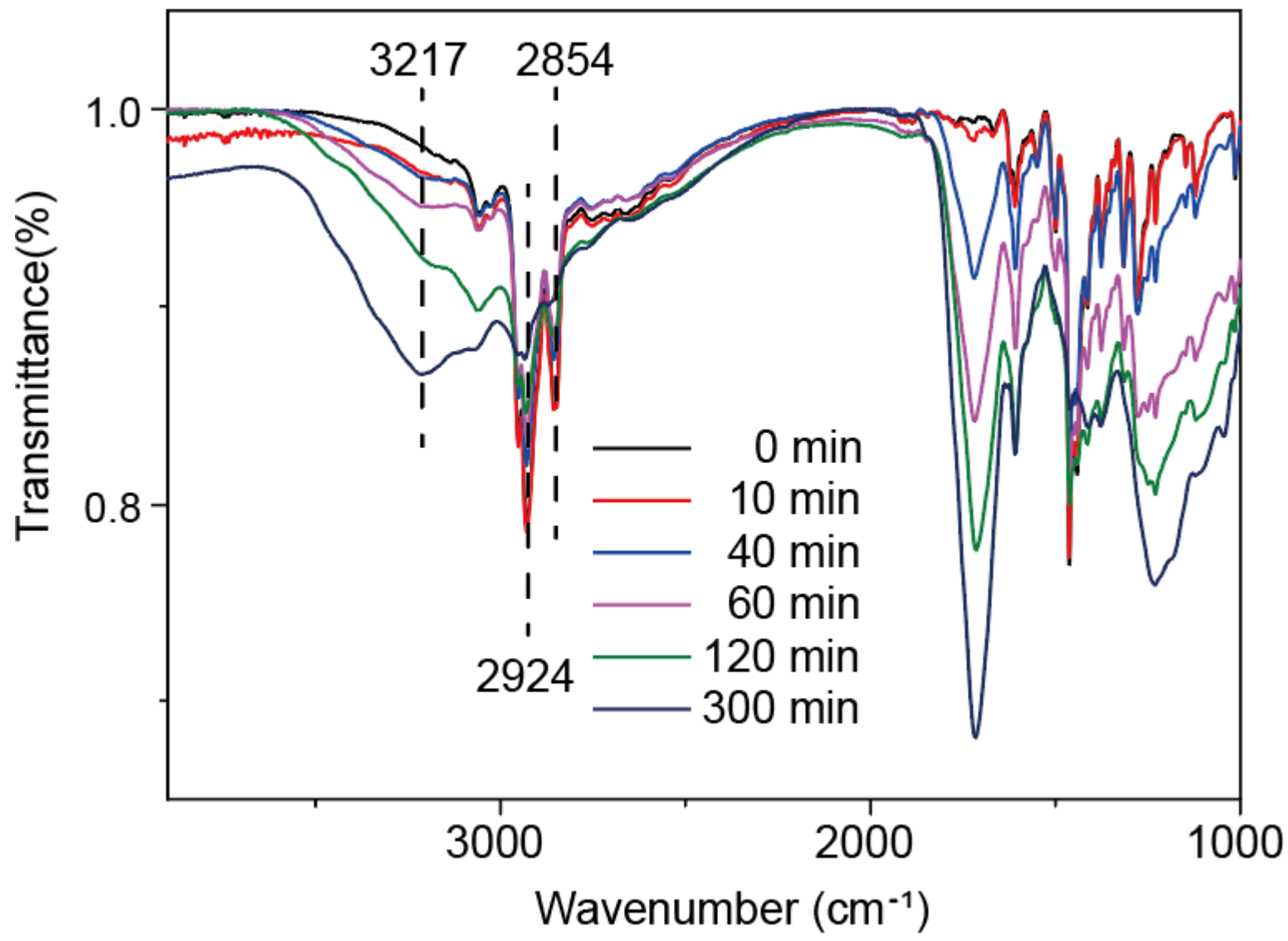

Supplementary Figure 15. The formation of oxidized polymers during the topochemical polymerization process. *In-situ* infrared spectrum of large-area single-crystalline film photoresist under different laser irradiation durations (480 nm, 50 µW) shows the characteristic peaks variation at 3217, 2924, and 2854 cm^{-1}. The broad vibration peak at 3217 cm^{-1}, representing the stretching vibration of oxygen-hydrogen bond[2], indicates the generation of hydroxy substitutes on the resulting polymers. The peaks diminishing at 2924 and 2854 cm^{-1}, representing the stretching vibration of carbon-carbon bond[2], indicates the cleavage of alkyl chain or aromatic ring groups during the photocarving process.

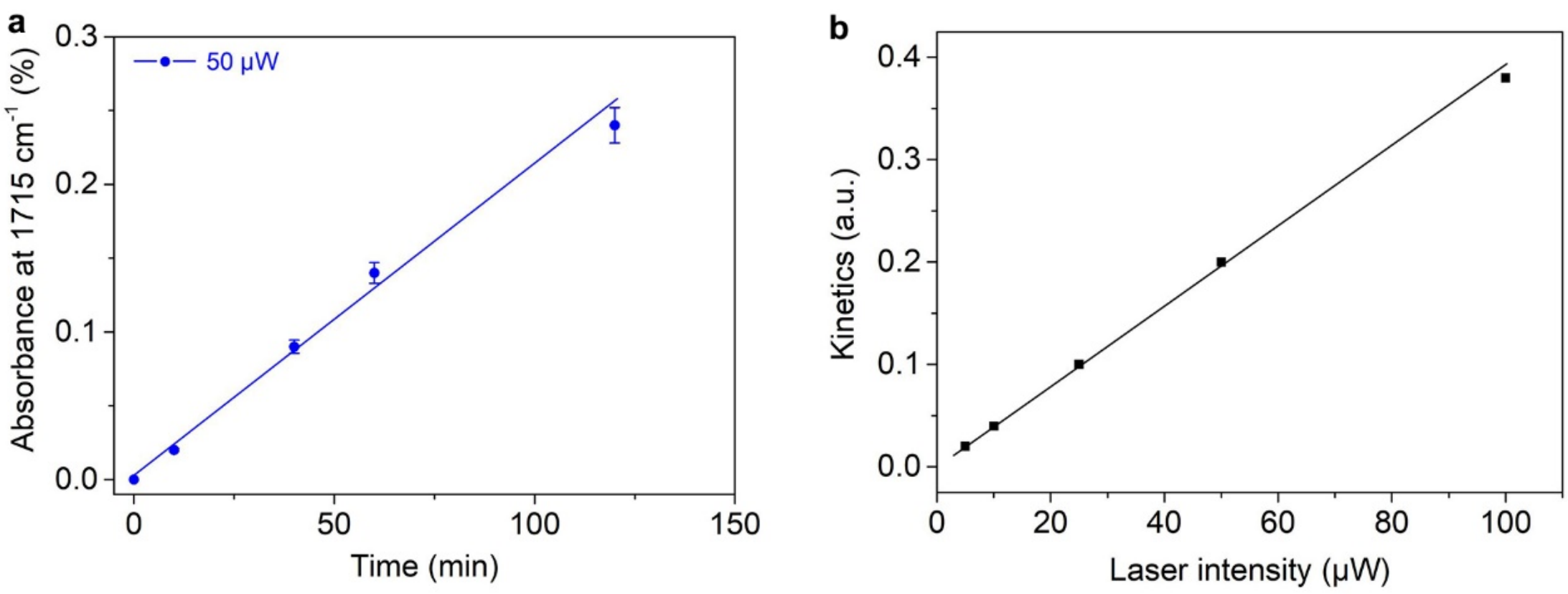

Supplementary Figure 16. The kinetics of the formation of oxidized polymers under visible laser irradiation at different intensities. a, The relationship between the absorbance variations at 1715 cm^{-1} as the function of irradiation time. **b**, The linear relationship between the kinetics of the oxidized polymer formation as the function of irradiation time.

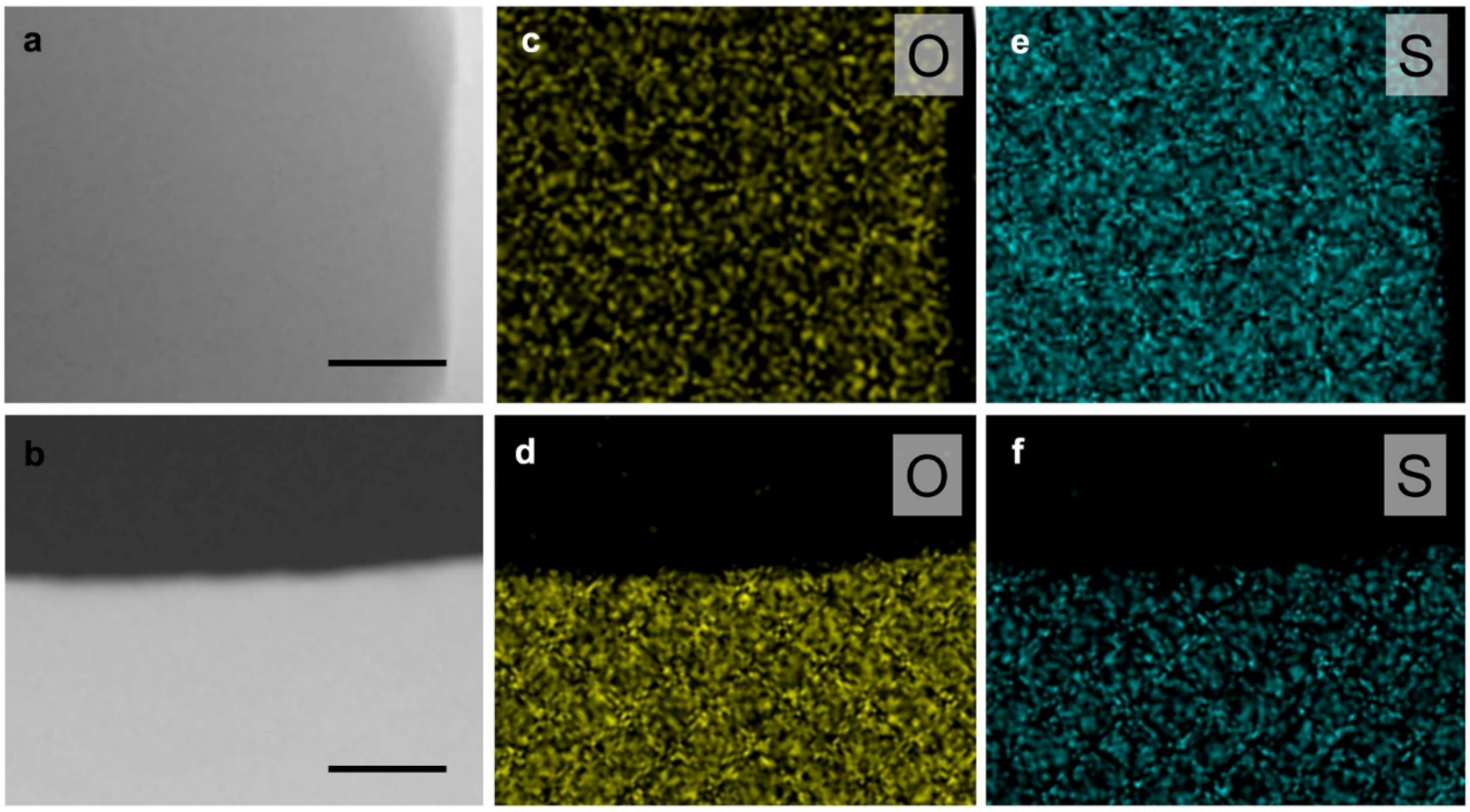

Supplementary Figure 17. Elemental analysis of single-crystalline film photoresists upon topochemical polymerization-based photocarving. Low-angle annular dark-field (LAADF) STEM images of single-crystalline film from **1** before (**a**) and after (**b**) photoirradiation by 470 nm continuous wavelength laser (20 μW). Scale bar = 300 nm. Corresponding SEM-EDX mapping images of the elementary distribution of oxygen (O) before (**c**, 0.1 w.t.%) and after (**d**, 11 w.t.%) photoirradiation, and sulfur (S) before (**e**, 3 w.t.%) and after (**f**, 2 w.t.%) photoirradiation.

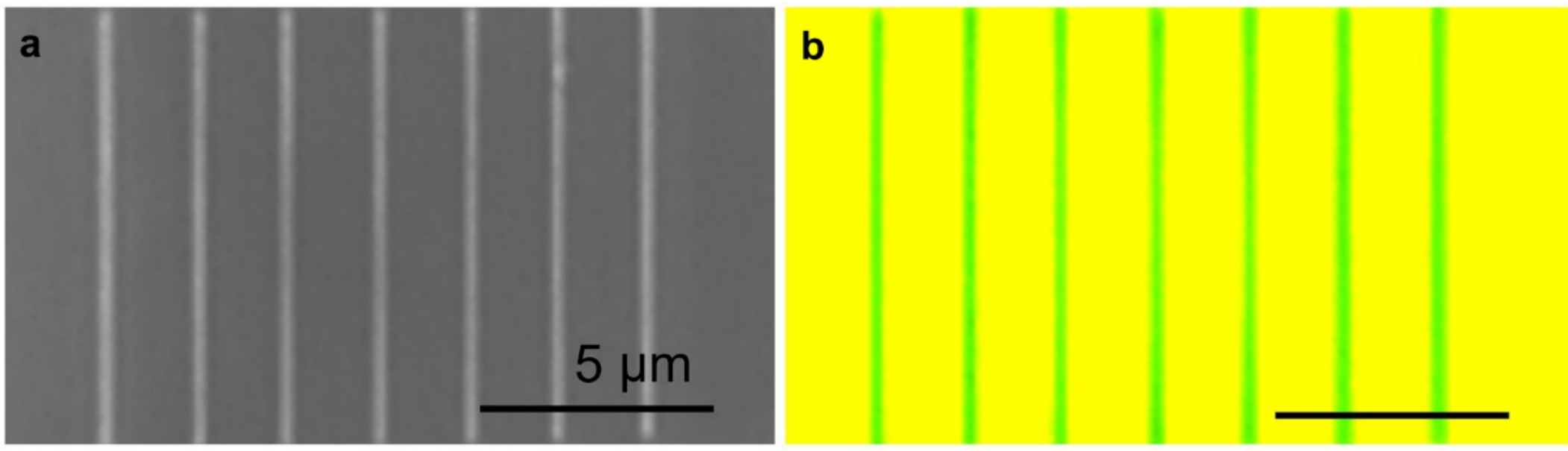

Supplementary Figure 18. Programmable topochemical polymerization-based photocarving on single-crystalline film using CW visible laser. a, Transmission mode and **b**, merged fluorescence mode confocal scanning laser microscopic images of the photocarved single-crystalline film from **1** after photocarving with linear arrays pattern.

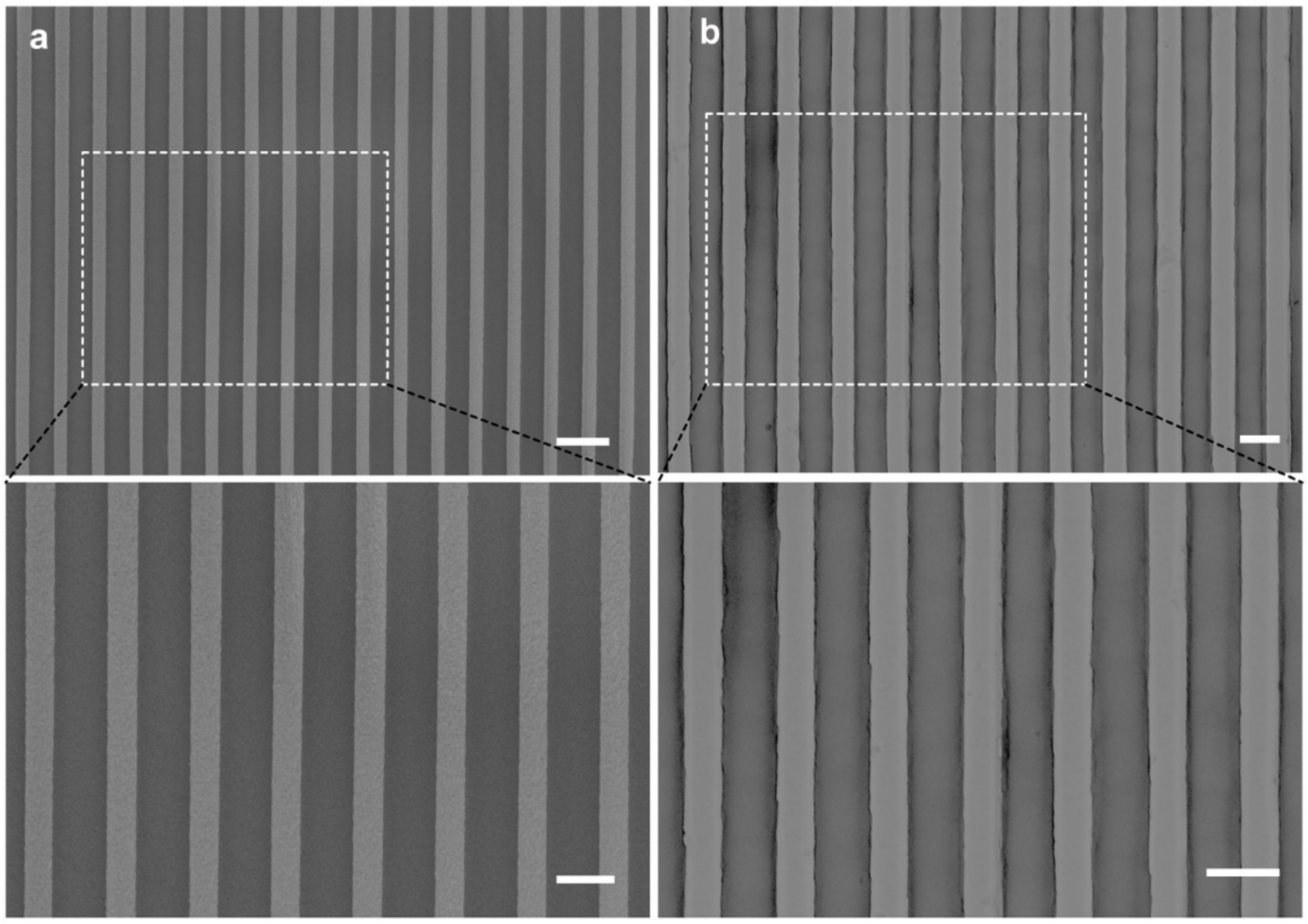

Supplementary Figure 19. Preparation of photocarved dense line patterns. Bright-field SEM images of photocarved 1:1 line-to-space dense line patterns on films from **1**, showing **a**, a resolution of 96 nm and a line-edge roughness (LER) of 2.4 nm, and **b**, a resolution of 63 nm and an LER of 3.5 nm. Scale bar = 400 nm (**a**), 200 nm (**a**, magnified), and 100 nm (**b**).

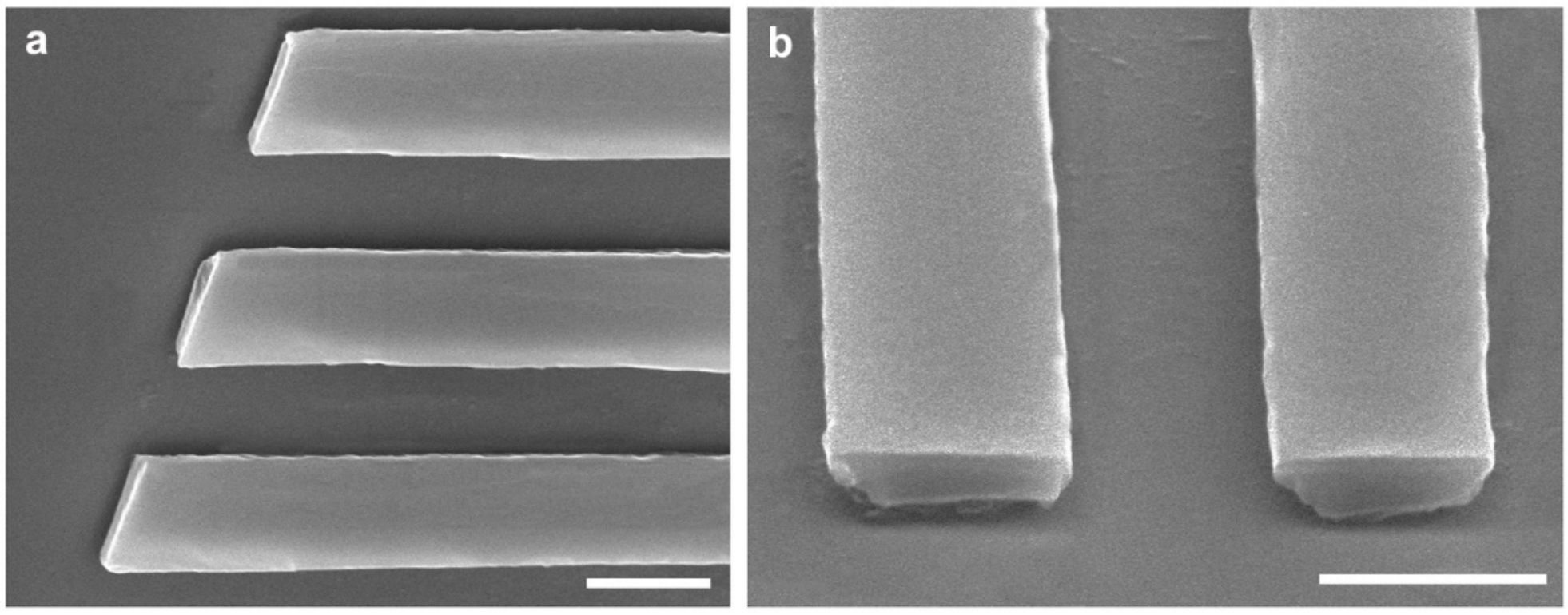

Supplementary Figure 20. Cross-sectional morphology of photocarved line patterns. a-b, Bright-field SEM images of the cross sections of photocarved line patterns on films of **1**. Scale bar = 200 nm.

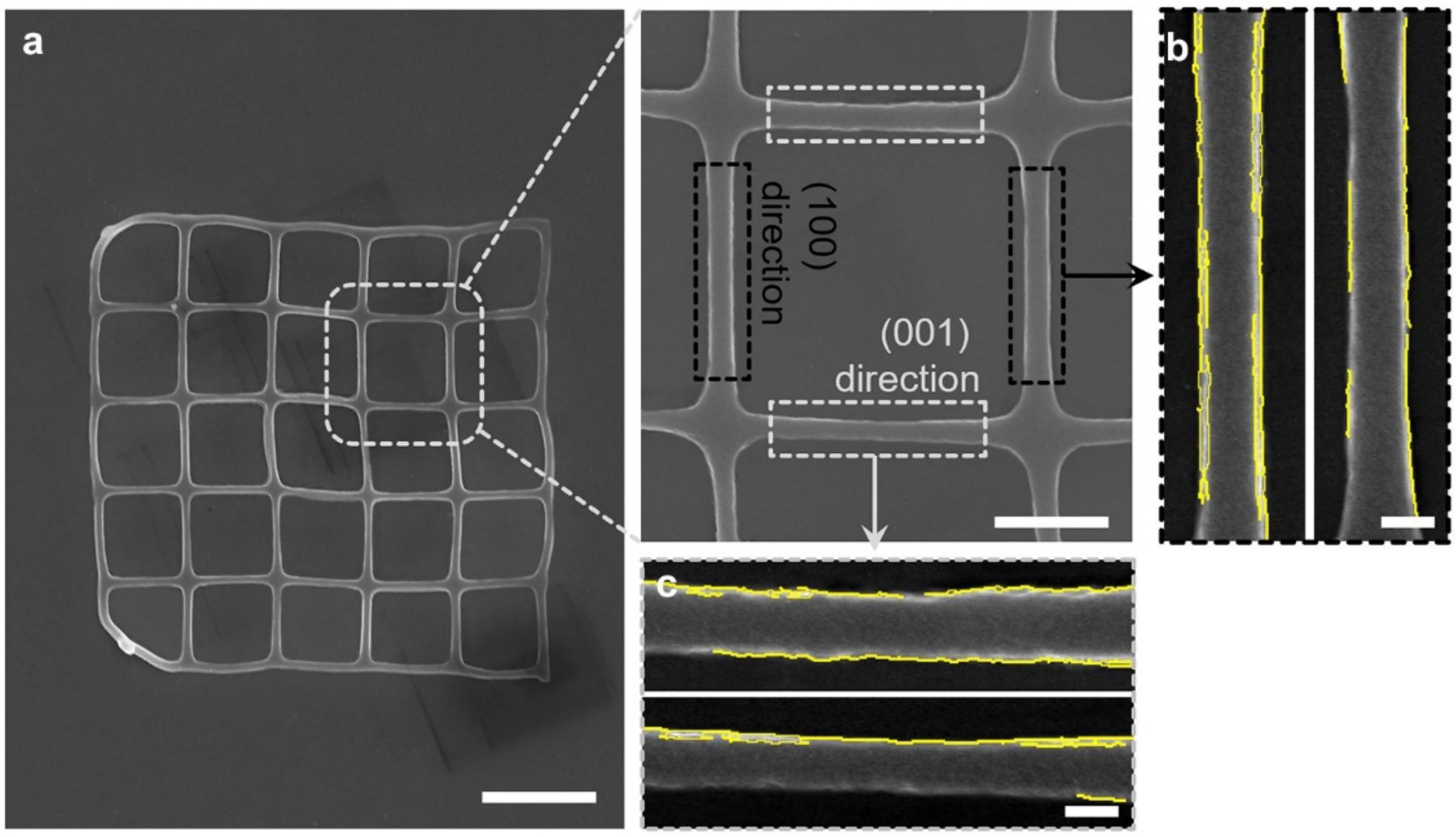

Supplementary Figure 21. Comparison of line-edge roughness (LER) along different facet directions. a, SEM images and enlarged view of photocarved square-pore grid patterns with orthogonal lines, align with the (001) and (100) facet directions. Scale bars = 1 μm and 500 nm (insets). **b**, Quantitative analysis of LER along the (100) direction, yielding values of 3.97 and 2.62 nm (black-dotted region). Scale bar = 100 nm. **c,** Quantitative analysis of LER along the (001) direction, yielding values of 3.46 and 2.94 nm (grey-dotted region). Scale bar = 100 nm.

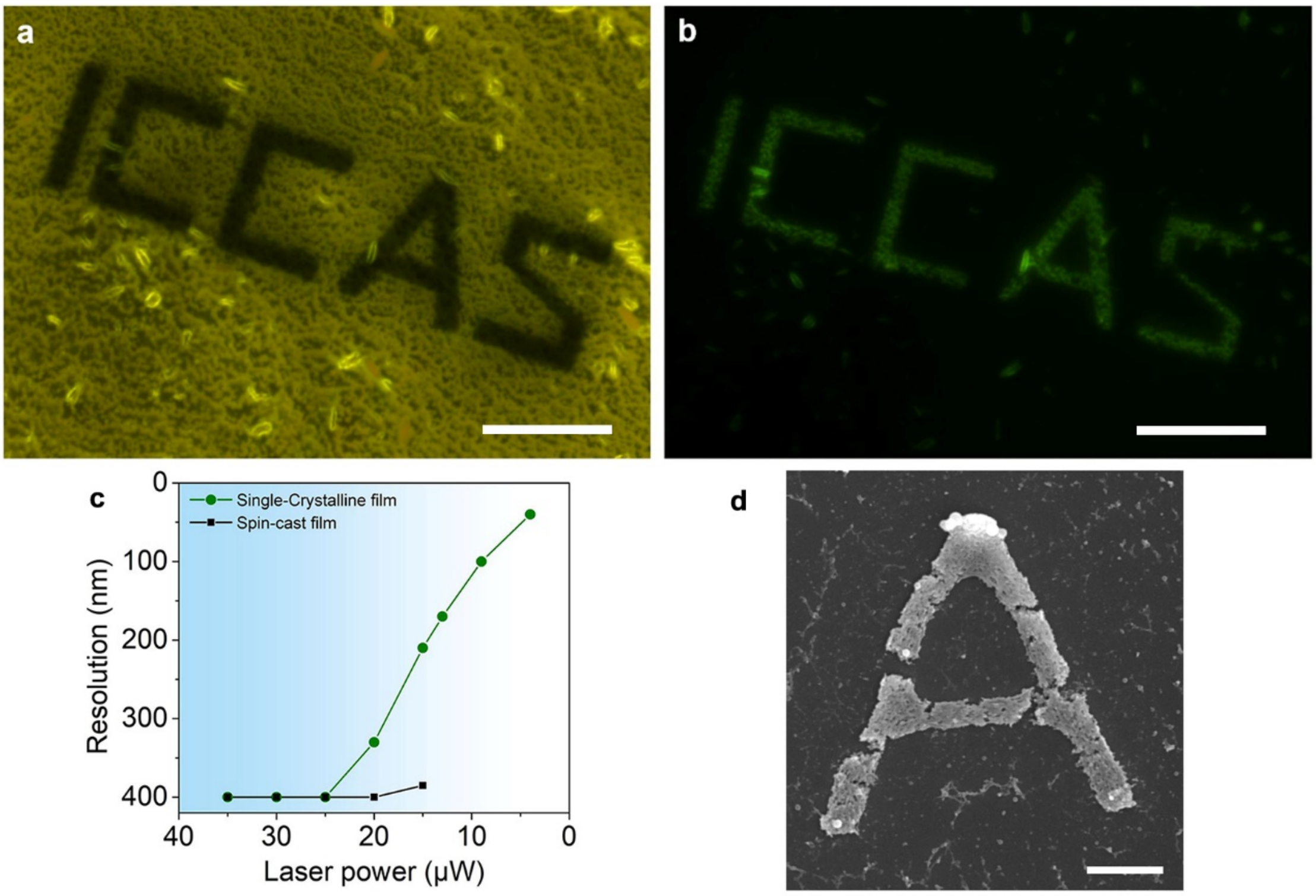

Supplementary Figure 22. Photocarving of amorphous film from 1. a, Fluorescence mode optical images of amorphous film from **1** after photocarving with a "ICCAS" pattern (470 nm, 20 μW). **b**, Fluorescence mode optical images of amorphous film from **1** after developed by negative mode developer. Scale bar = 10 μm. **c**, Comparison of the relationship between applied laser power and attainable photocarving resolution in single-crystalline film photoresists versus spin-cast film photoresists. **d**, SEM images of attained negative mode pattern 'A' with resolution of 389 ± 20 nm prepared from photocarving on spin-cast film from **1** after development.

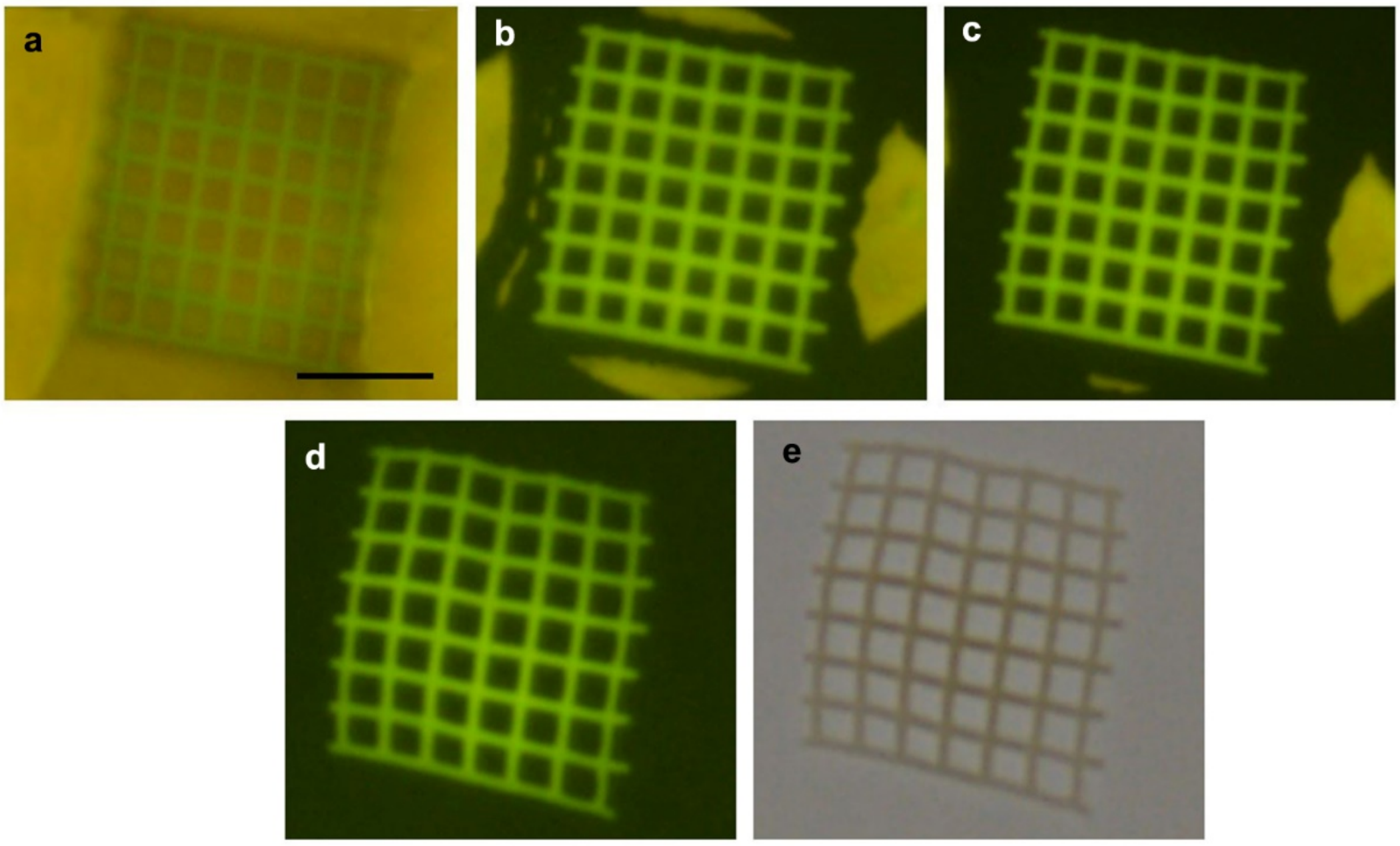

Supplementary Figure 23. The development process of photocarved patterns on single-crystalline film photoresists using continuous wavelength visible laser. Typical procedures for the development of photocarved patterns on large-area single-crystalline film photoresist from **1**. **a**, Fluorescence mode optical image of photocarved large-area single-crystalline film photoresist with a programmed "net" pattern. Scale bar = 5 μm. **b**, First and **c**, second round of dissolving unexposed regions using the negative developer. **d**, Fluorescence mode optical image of the remaining negative mode structure after washing with hexane. **e**, Transmission mode optical image of the resulting negative mode of photocarved structure on glass substrate.

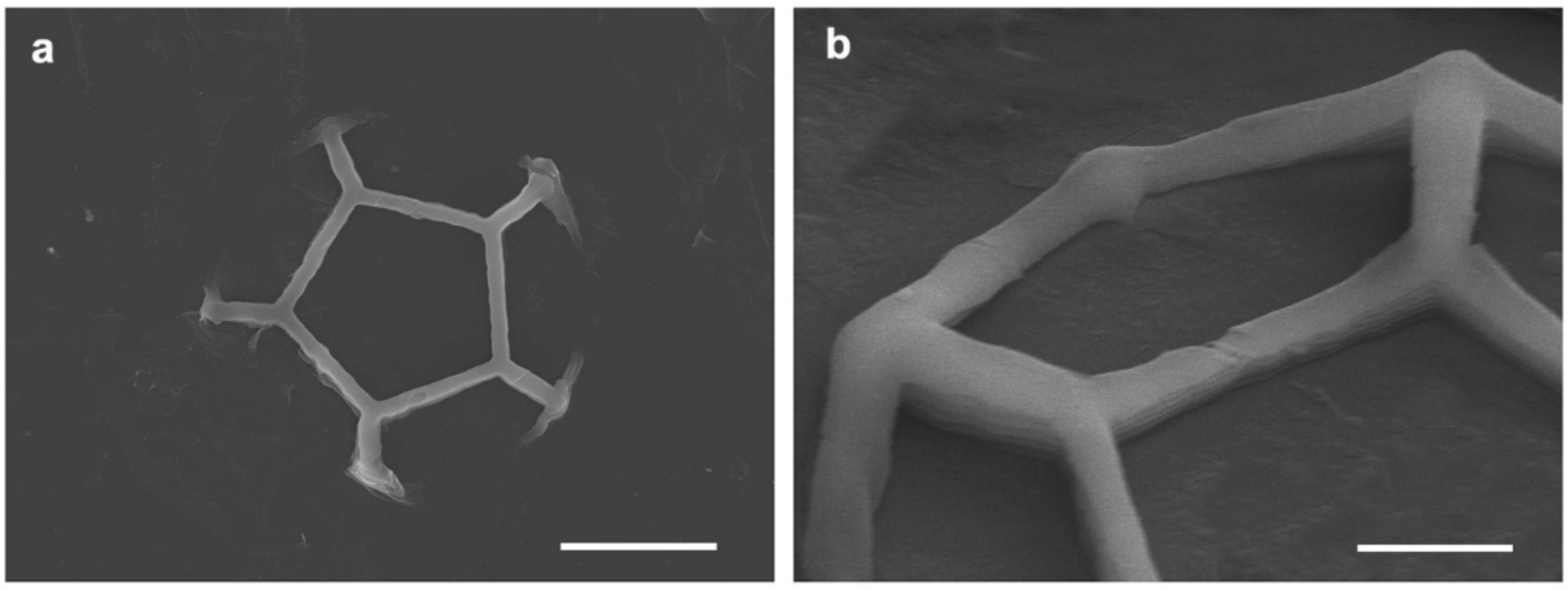

Supplementary Figure 24. 3D photocarving on spin-cast amorphous film. SEM images of the negative-mode dodecahedral 3D structure are presented in **a**, top view and **b**, 40-degree oblique view. Scale bar = 3 μm (**a**), and 2 μm (**b**).

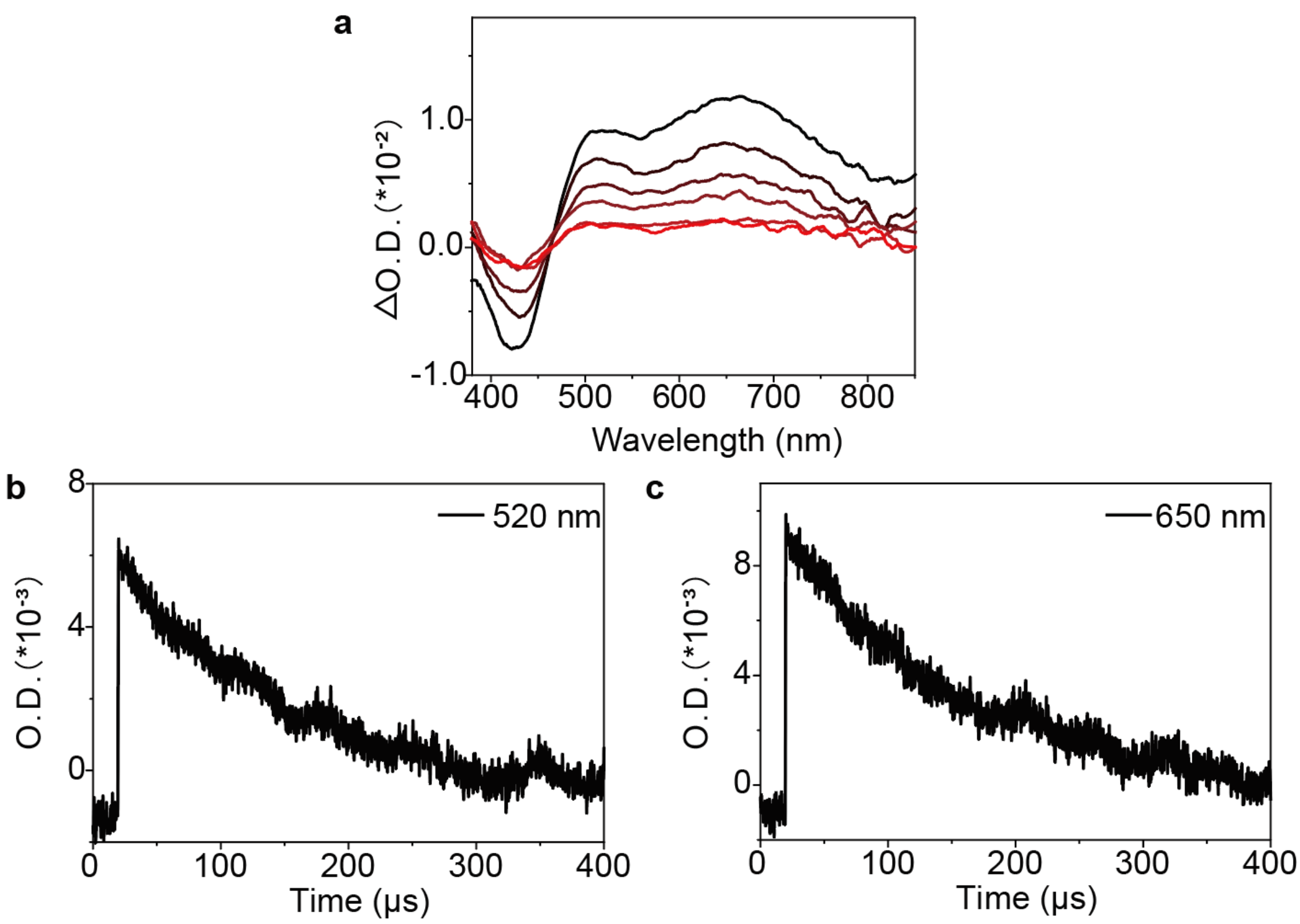

Supplementary Figure 25. Excited-state species investigation in photoinitiated topochemical polymerization. a, The flash photolysis spectrum of the large-area single-crystalline film photoresist exhibits an inverse peak spanning from 400 to 450 nm, attributed to the film's fluorescence emission. Additionally, a broad positive absorption ranging from 500 to 800 nm, exceeding a lifetime of 300 microseconds, characterizes benzophenone radicals[3]. **b**, The kinetic changes corresponding to the flash photolysis spectrum at the 520 nm position and **c**, the 650 nm position further corroborate the existence of radicals with a lifetime exceeding 300 microseconds.

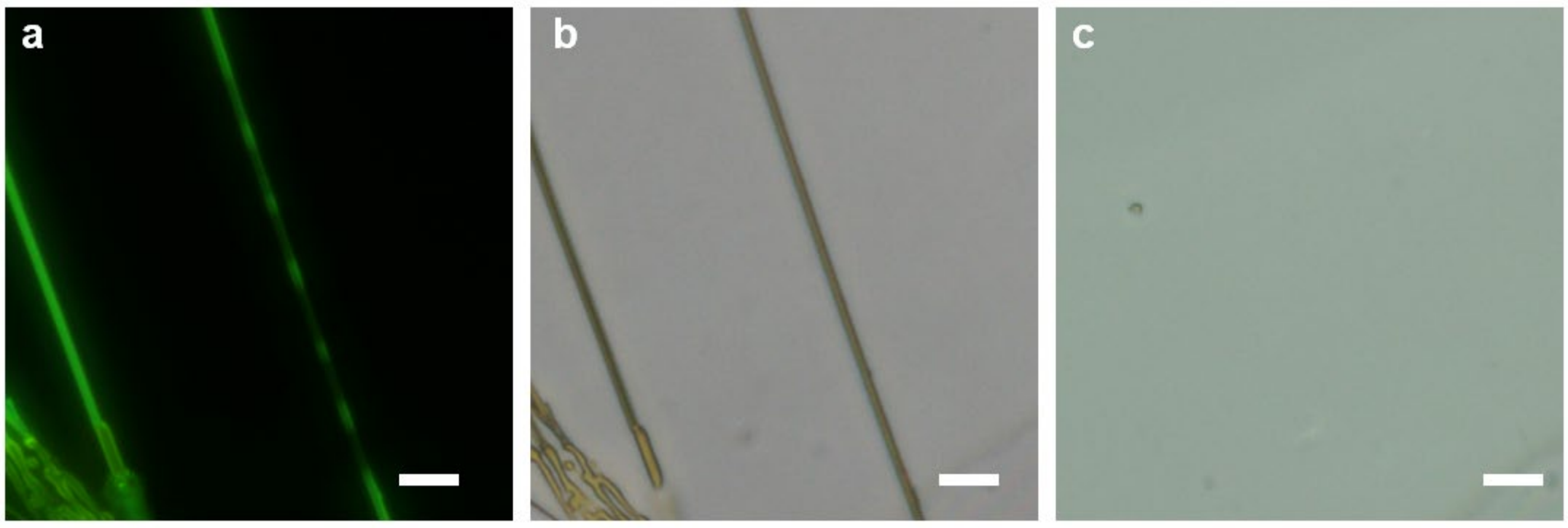

Supplementary Figure 26. Photocarving on crystals from 4. a, Fluorescence and **b**, transmission mode optical image of crystals from **4** after photocarving using visible laser (470 nm, 10 μW). Scale bar = 10 μm. **c**, Transmission mode optical image of glass substrate after developing photocarved crystals from **4**.

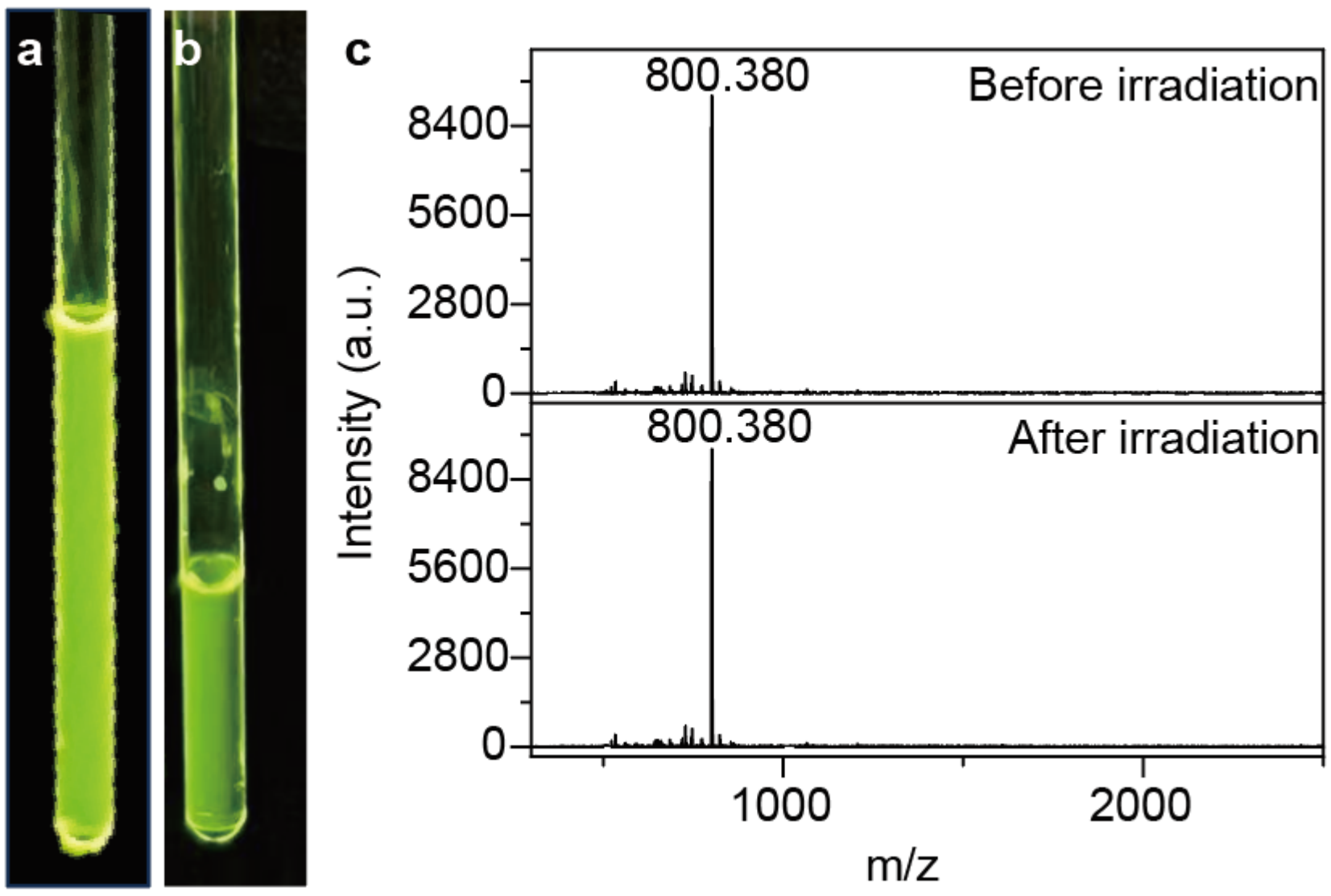

Supplementary Figure 27. Photoreactions investigation of molecule 4 on visible laser irradiation. The solution of monomers **4** before (**a**) and after (**b**) 4 hours of laser irradiation (470 nm, 10 μW) showed no fluorescence colour change. The decrease in solvent volume is attributed to the evaporation of the solvent (tetrahydrofuran) upon irradiation. **c**, MALDI-TOF analysis results of the molecular mass before and after 4 hours of visible laser irradiation.

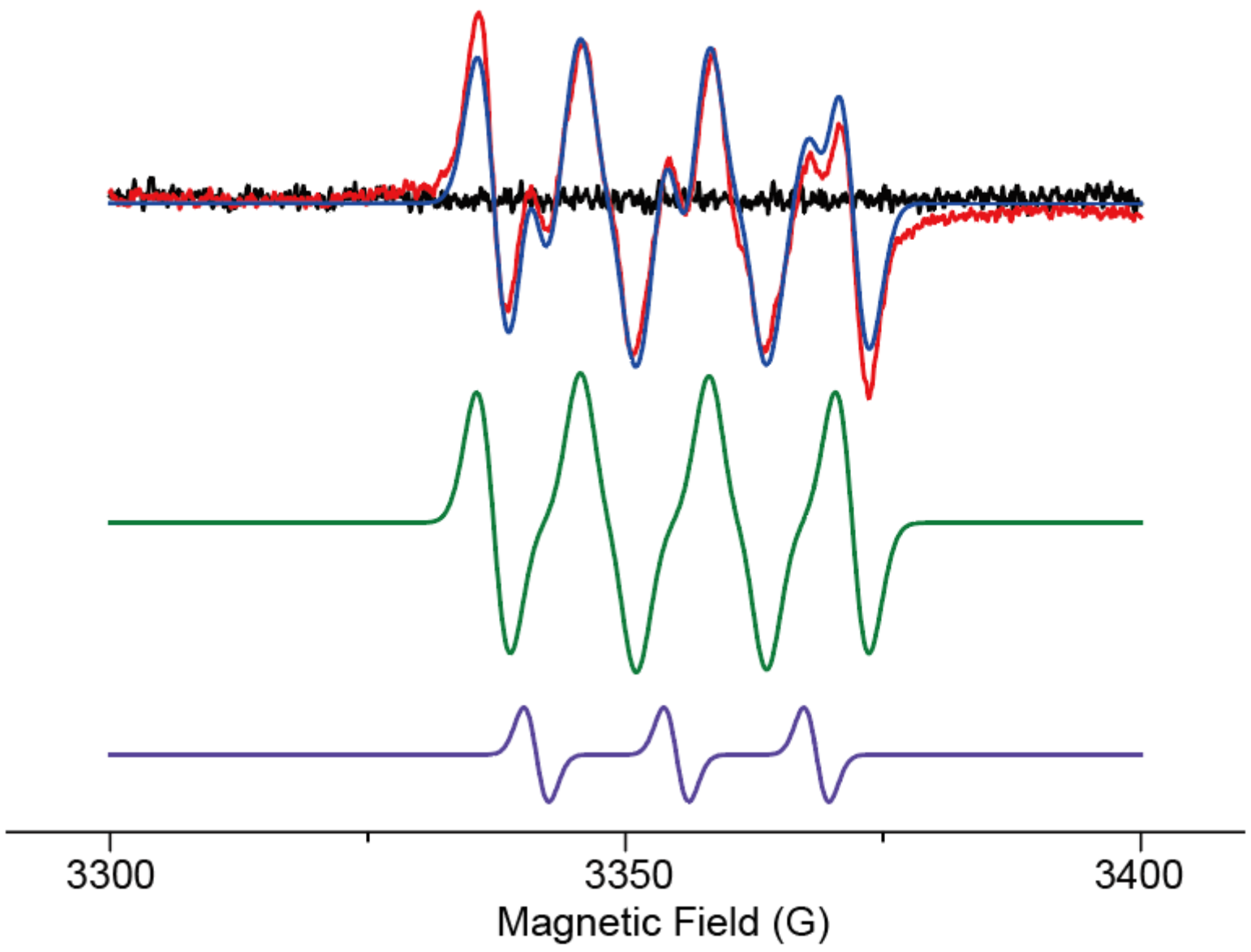

Supplementary Figure 28. The radical species of the photopolymerization. Photoinduced electron paramagnetic resonance (EPR) analysis of the photopolymerization of **1** was conducted with 470 nm laser irradiation (5 W) for 5 minutes (red curve) and without irradiation (black curve). The spectral despectrometry[4] results show that the EPR result under laser irradiation exhibits a mixed characteristic peak of radical species analogous to DMPO-OR• (purple line, g = 2.007, AN=12.56G, AH=9.75G)[5] and DMPO-O_2•$^-$ (green line, g = 2.00478, AN=13.63G)[6]. The backward simulation of the mixed EPR curve (blue line) from the two inferred radical species fits well with the experimental EPR curve (red line), further corroborating the validity of the inferred results.

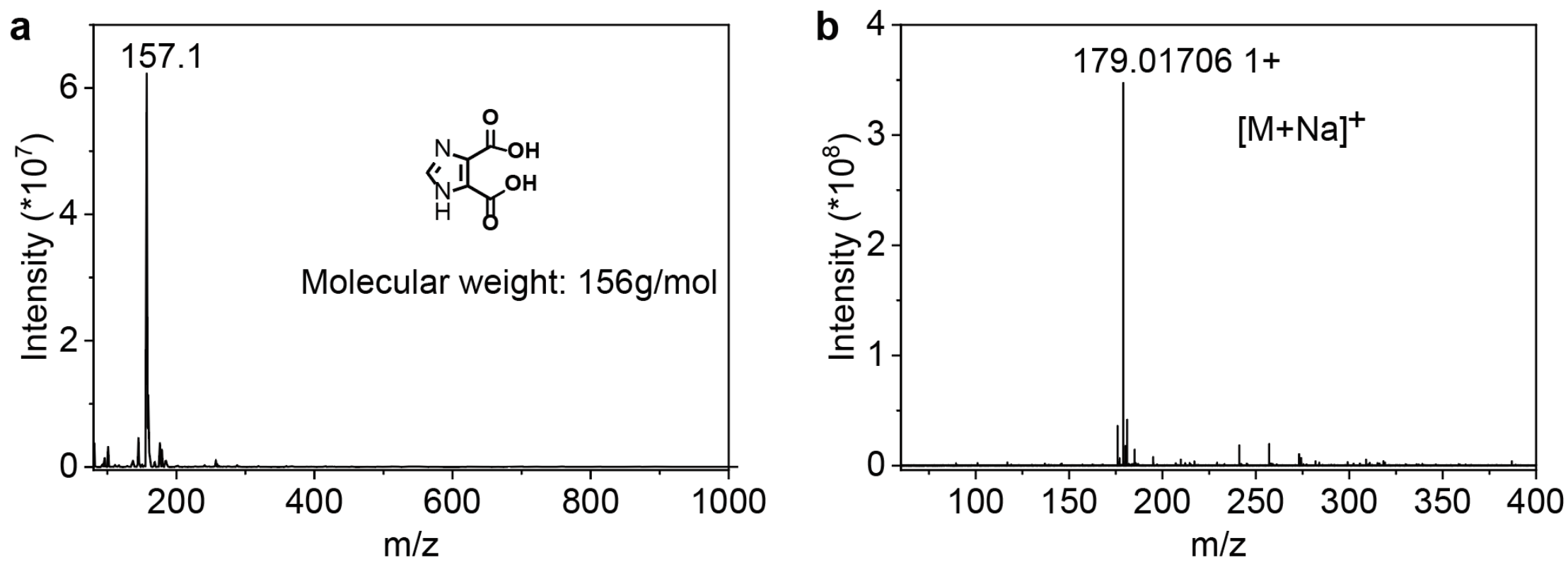

Supplementary Figure 29. The formation of oxidized products during the photocarving process. a, The high-performance liquid chromatography-mass spectrometry (HPLC-MS) analysis reveals a peak with a mass-to-charge ratio of 157 (m/z) after the photopolymerization reaction. **b**, High-resolution mass spectrometry analysis (HR-MS) shows a peak with a mass-to-charge ratio of 179 (m/z) after the photopolymerization reaction, suggesting the formation of a sodium ion complex by the product with a molecular weight of 156.

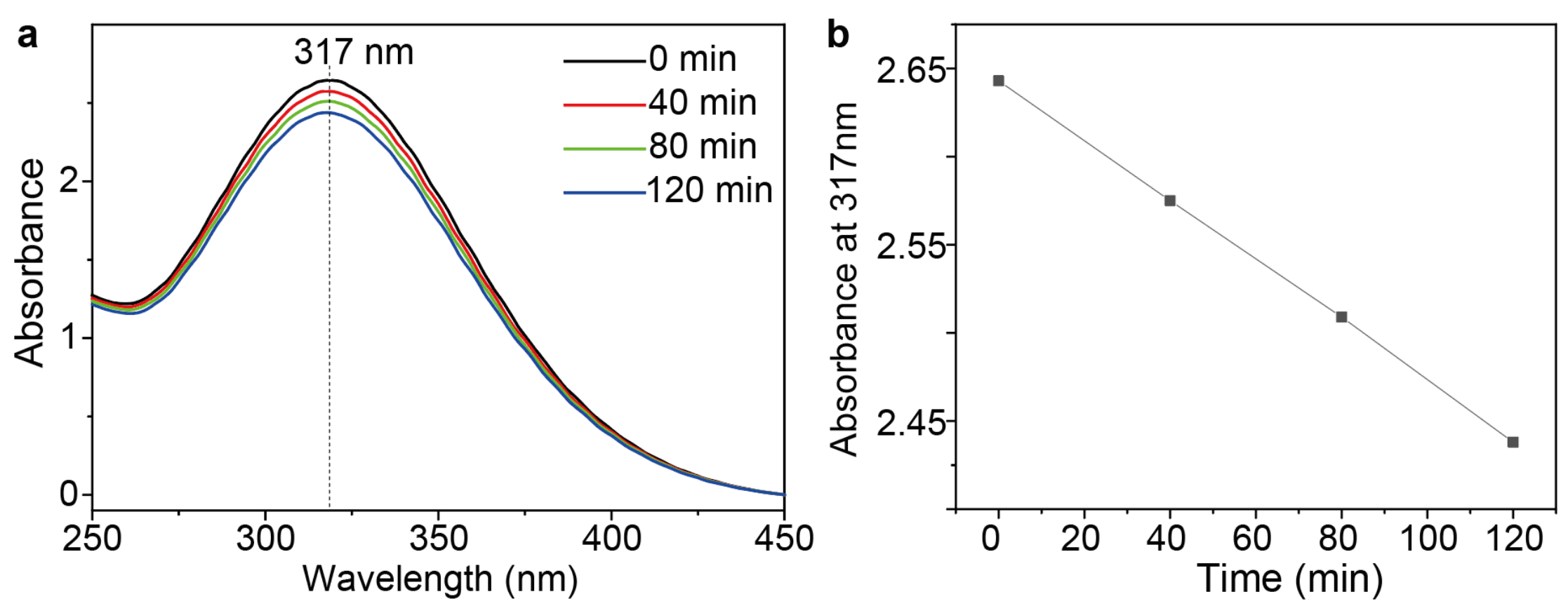

Supplementary Figure 30. Hydrogen dioxide detection during the photocarving process. a, Absorption spectrum variation of $Ce(SO_4)_2$ titration experiments at different irradiation durations (470 nm LED light, 5 mW) for 40, 80, and 120 mins. **b**, Plot of absorbance variations at 317 nm as a function of different irradiation durations.

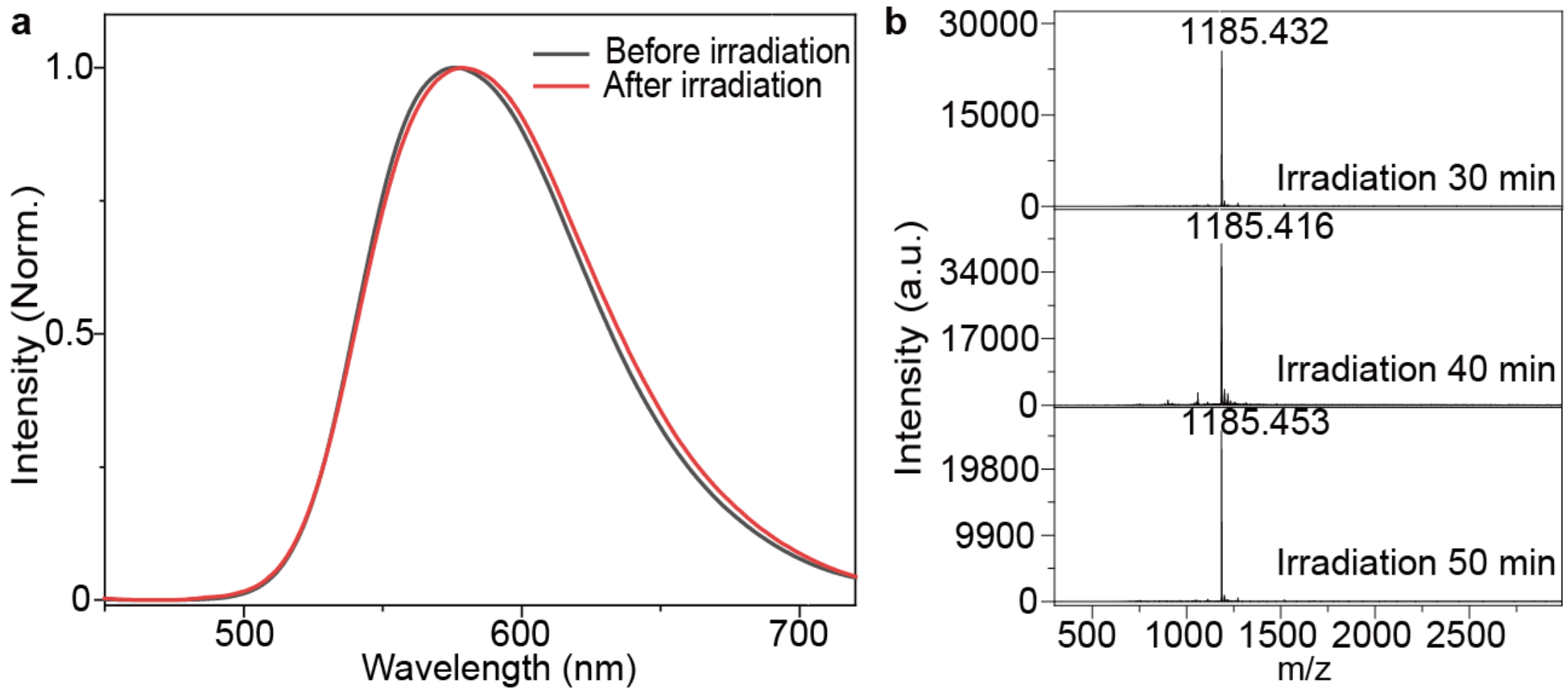

Supplementary Figure 31. Photoirradiation of single-crystalline film from 1 in argon. a, Fluorescence spectrum of single-crystalline film photoresists before (black) and after visible laser irradiation for 5 hours (470 nm, 20 μW). **b**, Mass spectrometry analysis of single-crystalline film photoresists from **1** under irradiation for different durations. The unaltered fluorescence emission and molecular weight result indicates the photoresists did not undergo topochemical polymerization under argon.

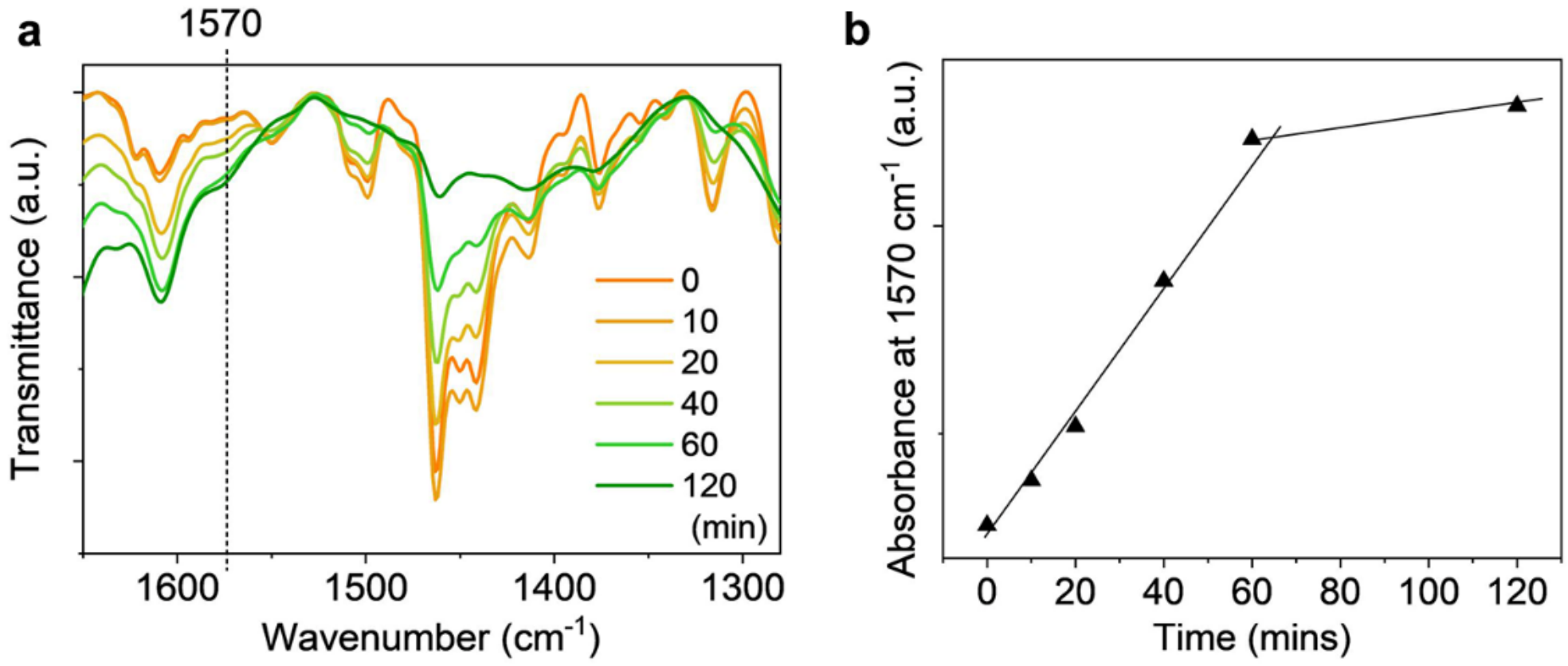

Supplementary Figure 32. *In-situ* infrared analysis of the photoinduced polymerization process. a-b, *In-situ* infrared spectra of a large-area single-crystalline film photoresist collected under different laser irradiation durations (480 nm, 50 μW). A new peak emerges at approximately 1570 cm^{-1} and evolves with irradiation time, which can be assigned to vibrations associated with N-O bonding[7]. This observation indicates the formation of oxygen-bridged linkages involving nitrogen atoms during the photoinduced polymerization process.

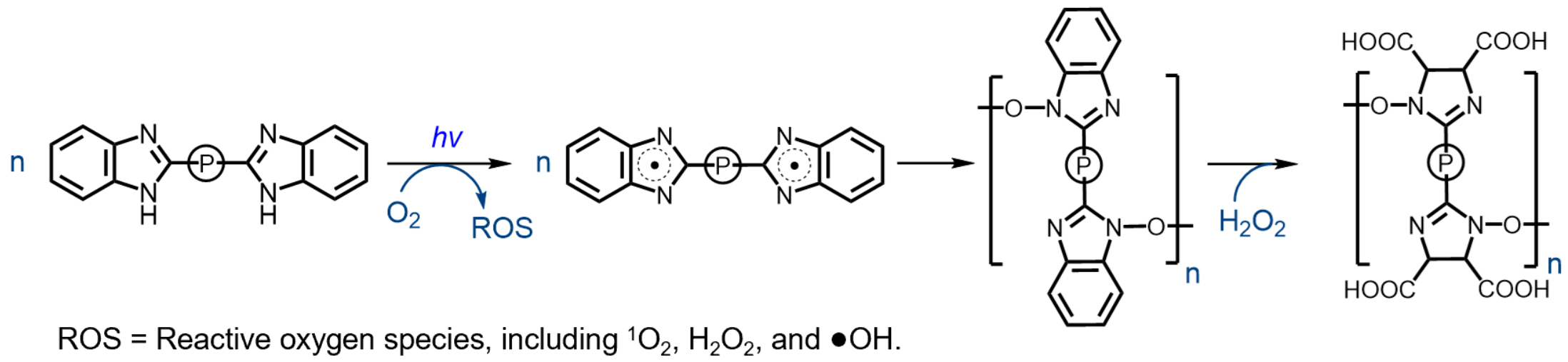

ROS = Reactive oxygen species, including $^1O_2$, H_2O_2, and ●OH.

Supplementary Figure 33. Detailed photoinduced topochemical polymerization mechanism.

Supplementary References

1 Gong, Y. *et al.* Unprecedented Small Molecule-Based Uniform Two-Dimensional Platelets with Tailorable Shapes and Sizes. *J. Am. Chem. Soc.* **144**, 15403-15410 (2022).

2 Infrared Spectra of Some Common Functional Groups.

3 Hatano, S., Horino, T., Tokita, A., Oshima, T. & Abe, J. Unusual Negative Photochromism via a Short-Lived Imidazolyl Radical of 1,1′-Binaphthyl-Bridged Imidazole Dimer. *J. Am. Chem. Soc.* **135**, 3164-3172 (2013).

4 Stoll, S. & Schweiger, A. EasySpin, a comprehensive software package for spectral simulation and analysis in EPR. *Journal of Magnetic Resonance* **178**, 42-55 (2006).

5 Dubose, C. M., Rehorek, D., Um, O. & Janzen, E. G. Spin trapping: ESR parameters of spin adducts. *J. Free radical biology medicine* **51**, 55-56 (1988).

6 Chen, L. *et al.* Accurate identification of radicals by in-situ electron paramagnetic resonance in ultraviolet-based homogenous advanced oxidation processes. *Water Research* **221**, 118747 (2022).

7 Miller, L. M. & Coates, J. P. in *Encyclopedia of Analytical Chemistry* 1-24 (2026).
