# Supplementary material for: High-performance topochemical polymerization-based photo-carving with sub-50 nm resolution utilizing visible light
Source: Nat Commun. 2026 Jun 25;17:7964. doi: 10.1038/s41467-026-73463-9 (PMC13448494; doi:10.1038/s41467-026-73463-9)
Supplement: Supplementary file 2 [file 41467_2026_73463_MOESM2_ESM.pdf]

nature portfolio

Peer Review File

High-performance topochemical polymerization-based photo-carving with sub-50 nm resolution utilizing visible light

Corresponding Author: Professor Yuchen Wu

This file contains all reviewer reports in order by version, followed by all author rebuttals in order by version.

Version 0:

Reviewer comments:

Reviewer #1

(Remarks to the Author)
Dear authors,

I see with your work nice advances in the photocarving lithography with respect to the state of the art. The work is overall well explained and supported by experiments and literature review. The comparison with other lithography technologies (iline, KrF, .. EUV) is welcomed, but I don't think the position of your work in terms of resolution can hit the ArF resolution. I suggest you review that part and differently explain what you meant for that.
It would be also interesting to discuss if your single-crystalline film photoresist could be expanded to other lithographic technologies or it is confined to the photocarving technique.
Maybe because I'm not familiar with the dual solid liquid interface method, but I'm not sure I understand the methodology, I suggest improving the illustration of the film preparation in figure 1. Kind regards.

Reviewer #2

(Remarks to the Author)
The authors report a topochemical polymerisation approach to achieve sub-50 nm patterns using donor–acceptor small molecules end-capped with heteroaromatic benzimidazole moieties. Building on a radical-coupling process between smaller-bandgap fluorescent molecules, they propose a photolithographic protocol driven by a visible-laser light source.

Comments and questions
- A key concern is the mechanism underlying the solubility switch of Compound 1 films. Because no solubility change is observed in the absence of oxygen molecules—often implicated in radical processes—pathways other than radical cross-linking between neighbouring imidazole nitrogens may be operative. Could radical reactions in the solubilising hexyl chains (e.g., H-abstraction and subsequent cross-coupling) contribute, given that aliphatic sp3(C)–H bonds have lower bond-dissociation energies (BDEs) than aromatic N–H? In addition, the proposed oxidation of the benzimidazole ring to a bis-carboxylic acid (Fig. 2b) is difficult to accept without stronger literature support.

- On page 7, the text suggests that propanol may participate in intermolecular cross-linking. Has propanol been detected in the bulk by single-crystal X-ray analysis, or otherwise verified spectroscopically?

- On page 4, the statement "Photosensitive monomers comprising imidazole moieties were designed for photoinduced topochemical polymerization and preferential pre-arrangement in the form of crystalline film" lacks citations. Please substantiate this design rationale with appropriate literature precedents.

- The energy of the incident laser beam required to induce the solubility change in Compound 1 films should be reported. Given the timescale in Fig. 2k, the required dose appears substantial; quantitative data are needed.

- No image for LER extraction is provided, and the analysis conditions and software are not described.

- For relevance to integrated-circuit photolithography, demonstration of dense patterns (1:1 line/space) is needed rather than

isolated lines. Cross-section images of densely patterned lines are strongly recommended.

- Can the LER of line patterns be quantitatively compared for patterns formed on different crystal growth faces?

- The developer solutions listed on page 7 appear to differ from those in Supporting Information. Please reconcile these discrepancies.

The approach shows promise for building nanostructures with a relatively simple fabrication setup. However, critical mechanistic evidence and practical details are missing. Additional data and clarifications are needed before the manuscript can be considered for publication or serve as a reliable guide for the community.

Reviewer #3

(Remarks to the Author)
In this paper, authors have fabricated large-area single-crystal thin films via a dual solid-liquid interface confinement strategy. The confinement strategy utilizes directed fluid flow to suppress uncontrolled nucleation, facilitating the creation of large-area single-crystal thin-film photoresists with tunable dimensions. The designed monomers feature benzimidazole moieties at their termini and authors have explored photo-induced radical formation and dimerization of benzimidazole ring for topochemical polymerization. Authors demonstrated photocarving on these photoresists, achieving resolutions below 50 nm using an ultra-low-power continuous-wave visible laser. The process is compatible with standard commercial optical microscopes, and this research introduces a new framework for sub-diffractional lithography. This paper is interesting, but requires major revision before publication in Nat. Commun.
Comments:
1) Some recent reviews on topochemical polymerization (eg. Chem. Sci. 2021, 12, 5361) is missing in the reference list
2) Tacticity refers to the relative stereochemical arrangement of pendant groups. I don't see a group that can generate tacticity in the polymer mentioned here. Authors may clarify what they meant by isotactic?
3) Authors cited 2D porous frame work in refs 7-10. Two recent studies on 2D polymers synthesis by topochemical polymerization (Nat. Commun. 2024, 15, 6638 and JACS 2025, 147, 37798) is missing in this list.
4) GIWAXs suggest crystallinity. How the authors know the film is single-crystalline in nature? What proof authors have to rule out multiple platelets ? For example, how do they rule out minute cracks?
Authors state that "Successful topochemical addition reactions have been reported in crystal lattices involving diverse reactive groups, including alkynes/azide4,18,19, alkene/azides20, olefins21-23, diynes17,24,25, triynes26-28, dienes29-31, trienes29, para-quinodimethanes32-36 and bis(indanone)s14,37,38". In this context, the recent examples of polymerisation via Topochemical Diels-Alder Cycloaddition -TDAC (JACS 2024, 146, 30495) and Topochemical alkyne Nitril-oxide cycloaddition -TANOC (Angew. Chem. Int. Ed., 2025, 64, e202520947) may be included.
5) Authors stated that "The single-crystalline nature of the mono-patch film from 1 was confirmed through well-defined diffraction peaks, the absence of diffraction rings, and consistent lattice parameters obtained from grazing-incidence wide-angle X-ray scattering (GIWAXS) measurements". The absence of diffraction ring means that the sample is NOT crystalline! Authors may clarify this.
6) Authors state that "Common crystal forms—such as plate-like, rod-like, or sheet-like structures—are prone to stress accumulation during conformational changes in topochemical reactions, often resulting in fracture or cracking." A reference for this statement is essential (JACS 2024, 146, 27100)
7) What happens to the solvent (propanol) which was hydrogen bonded to the imidazole unit, upon polymerization?
8) What are the circular patches in Fig 2 fluorescent microscopic images, apparently with grain boundaries? Does it say that the film is not a continuous crystal?
9) Same issue is with supplementary Fig 11
10) In figure 2H, the crystal packing of the polymer, the N-N single-bond distance between two imidazole rings seems to be unusually too long.
11) The absorption spectra shows that there is no absorption after 450 nm. Then how the irradiation using 488 nm effect the reaction?
12) The heading "Programmed 2D and 3D topochemical polymerization-based photocarving" and the description is mis-leading. topochemical 2D and 3D topochemical polymerization refer to 2D polymer /3D polymer synthesis via topochemical polymerization. What authors have achieved is just photocarving in a plane or 3D. The heading and the text should be rephrased.
13) Page 10: large-are should be large-area
14) Authors stated that "In contrast, no topochemical polymerization occurred on single-crystalline film photoresists in the absence of oxygen". What is the role of oxygen in topochemical polymerization? If the benzimidazole radical is formed under photoirradiation, why can't it just dimerize? Does it mean that the benzimidazole units are connected via peroxy linkage than N-N single bond? The higher oxygen content after photoirradiation is also supportive of this argument. Additionally, the distance between heterocyclic rings in Fig 2H also suggested a larger separation.

Version 1:

Reviewer comments:

Reviewer #2

(Remarks to the Author)
I found that the authors made efforts to address the questions and comments. My only remaining concern is the structural feasibility of the proposed covalent linkage between monomer 1 molecules. The authors suggest the formation of an “N–O–O–N” bond; however, in light of Ref. 81 (S. Hatano et al., J. Am. Chem. Soc. 2013, 135, 3164–3172), it seems plausible that alternative bond structures could form between the benzimidazole moieties of neighboring monomers, for example a “C-O-O-C” or “C-O-C” bond. Therefore, it is strongly recommended that the authors experimentally confirm the nature of the covalent bond formed between two monomer 1 molecules before publication.

Reviewer #3

(Remarks to the Author)
Authors have addressed all the comments raised by me in a satisfactory manner. The explanations and additions in teh manuscript are convincing . Now the paper has become much better. I recommend acceptance.

Open Access This Peer Review File is licensed under a Creative Commons Attribution 4.0 International License, which permits use, sharing, adaptation, distribution and reproduction in any medium or format, as long as you give appropriate credit to the original author(s) and the source, provide a link to the Creative Commons license, and indicate if changes were made.
In cases where reviewers are anonymous, credit should be given to 'Anonymous Referee' and the source.
The images or other third party material in this Peer Review File are included in the article’s Creative Commons license, unless indicated otherwise in a credit line to the material. If material is not included in the article’s Creative Commons license and your intended use is not permitted by statutory regulation or exceeds the permitted use, you will need to obtain permission directly from the copyright holder.
To view a copy of this license, visit https://creativecommons.org/licenses/by/4.0/

Responses to the Reviewers' Comments:

We have addressed the Reviewer's comments in the order R1-3.

Reviewer 1 (R1):

The reviewer makes very positive comments. "I see with your work nice advances in the photocarving lithography with respect to the state of the art. The work is overall well explained and supported by experiments and literature review."

Revisions suggested by R1 and our responses are outlined below:

Comment 1: *The comparison with other lithography technologies (iline, KrF, EUV) is welcomed, but I don't think the position of your work in terms of resolution can hit the ArF resolution. I suggest you review that part and differently explain what you meant for that.*

Response 1: We thank the reviewer for their suggestion. We agree that the resolution of our single-crystalline photoresist direct-photocarving technique, which is comparable to dry ArF lithography (approximately 90 nm), still falls short of the resolution achieved by immersion ArF photolithography. Following the reviewer's advice, we have revised the description to specify "dry ArF" instead of "ArF." In our future work, we aim to further develop excitation sources with shorter wavelengths to improve the resolution of our single-crystalline photoresist direct-writing technique to be comparable to that of immerse ArF photolithography.

Comment 2: *It would be also interesting to discuss if your single-crystalline film photoresist could be expanded to other lithographic technologies or it is confined to the photocarving technique.*

Response 2: We thank the reviewer for their valuable suggestion. Since the photolithography technique primarily relies on area light source exposure, it does not involve the focal intensity gradients that occur in point-source photocarving. As a result, the super-resolution advantages of our photoresist are less likely to be fully realized under conventional photolithography conditions. Moreover, although the reported two-dimensional single-crystalline photoresist exhibits noticeable absorption around 200 nm (figure below) and could, in principle, be activated by DUV or ArF beam sources, photolithography typically requires the mask to be in direct contact with the resist. This contact may pose a risk of mechanically damaging the single-crystalline film photoresist. Therefore, its actual performance needs to be evaluated using real photolithography equipment, and we plan to investigate its potential applicability in such systems in our future work.

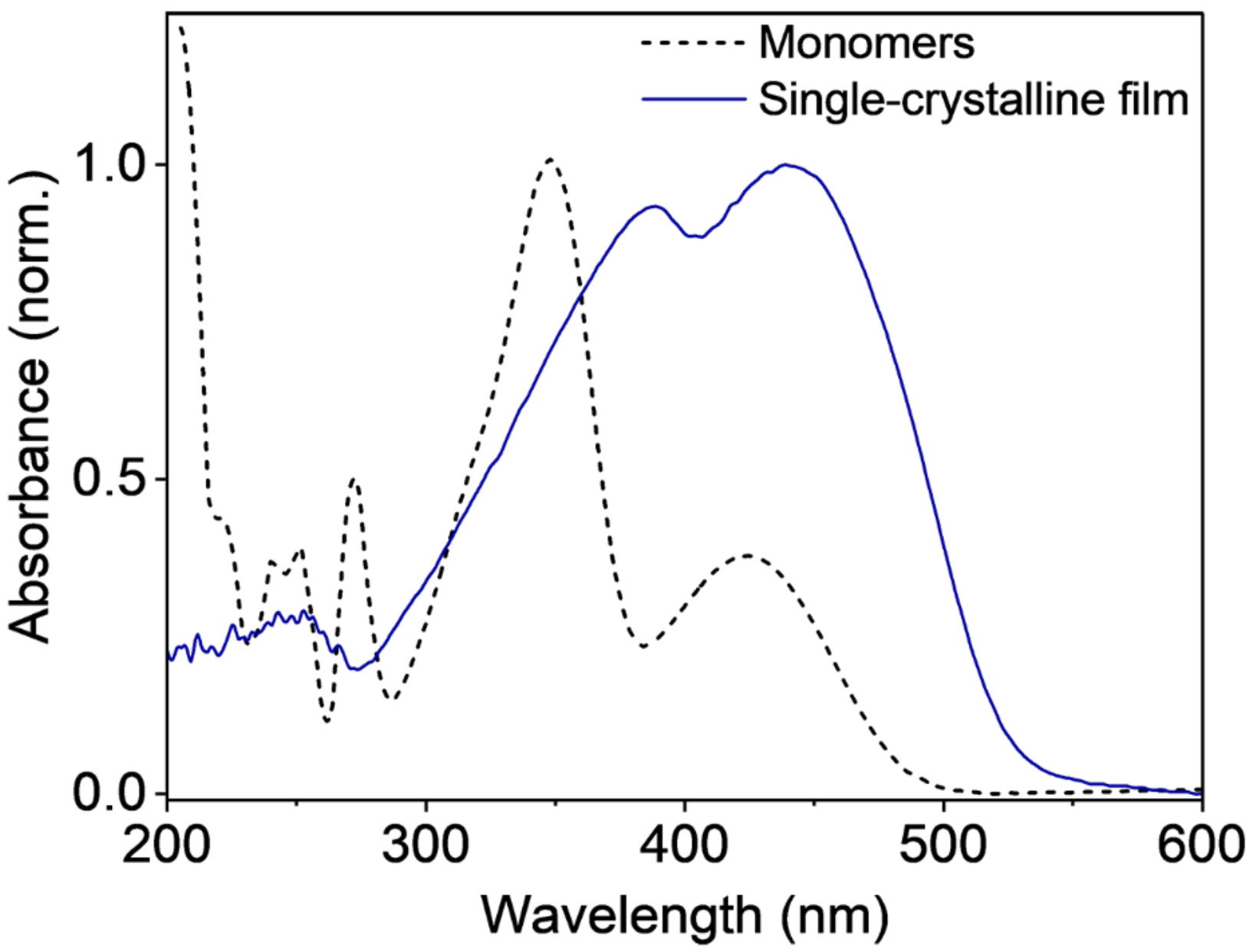

Figure. UV-vis absorption of monomer solution in THF (black dashed line) and single-crystalline film (blue solid line) from **1**.

Comment 3: *I suggest improving the illustration of the film preparation in figure 1.*

Response 3: We thank the reviewer for this helpful suggestion. To improve the clarity of the dual solid–liquid interface method and facilitate understanding for readers who may be less familiar with this technique, Figure 1 has been revised to provide a clearer, stepwise schematic of the film preparation process. The figure caption and the corresponding description have also been expanded to more explicitly define the key experimental steps and crystallization process. In particular, we have added annotations illustrating how the solute concentration evolves within the capillary bridge at the top of micropillar during solvent evaporation, together with the associated nucleation and crystallization processes. Because the three-phase contact line (TCL, C_{TCL}) exhibits more efficient solvent volatilization and diffusion than the solution interior (C_{Bulk}), an asymmetric evaporation environment is established within the capillary bridge. This asymmetry induces solvent transport towards TCL to compensate for evaporative loss, resulting in a progressive redistribution of solute concentration. Consistent with this picture, *in situ* optical microscopy observations provided in Supplementary Fig. 5 show that solvent evaporation leads to the formation of a localized high-concentration region at the three-phase line ($C_{TCL} > C_{Bulk}$). This region acts as a preferential nucleation site, thereby enabling the oriented growth of crystals.

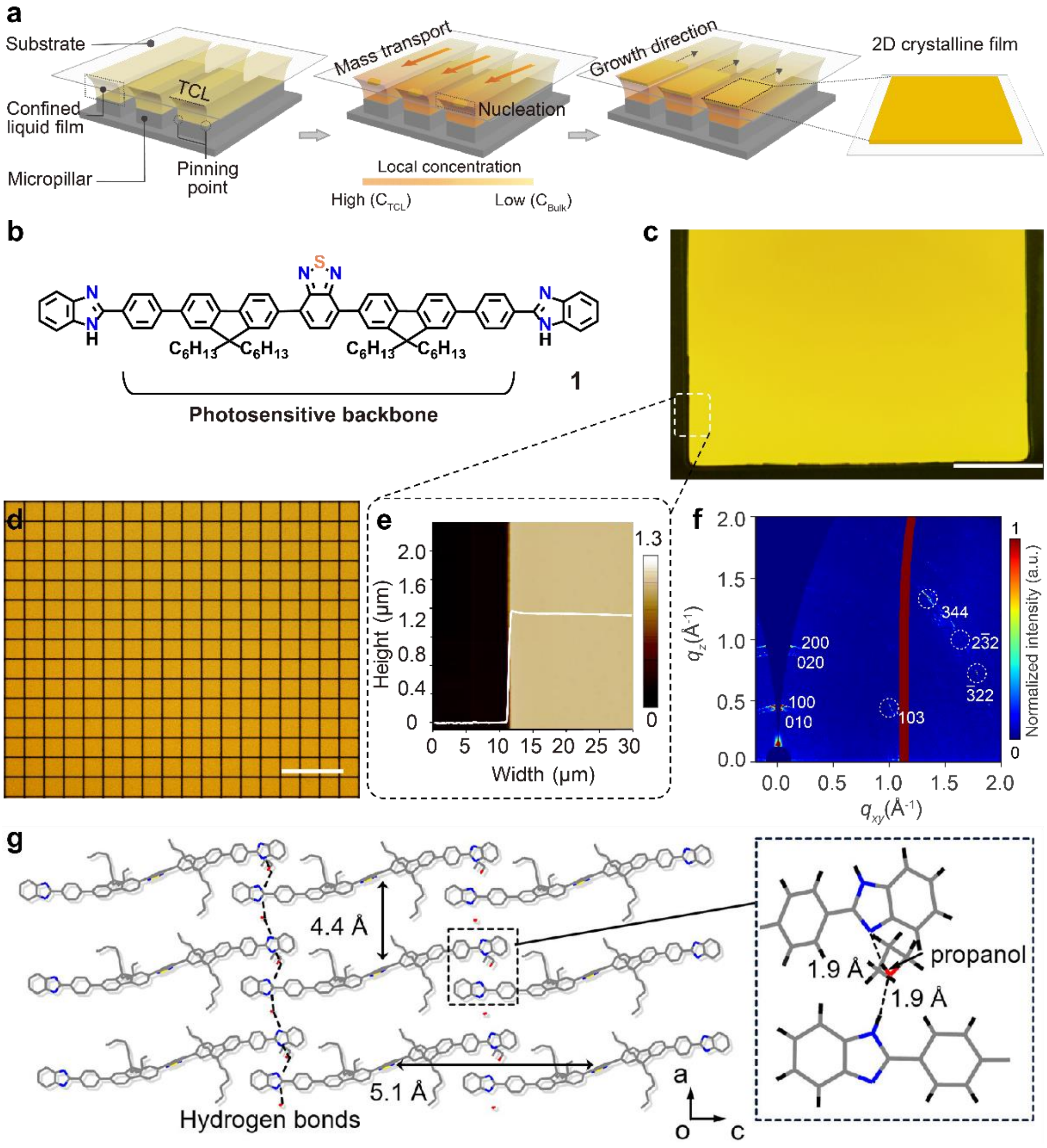

Fig. 1 | Preparation of large-area highly oriented crystalline film photoresists. **a**, Illustration of the preparation of large area highly oriented crystalline film through dual solid-liquid interface-confined assembly method. **b**, Molecular structure of monomer **1**. **c**, Fluorescence microscopic image of the large-area mono-patchy highly oriented crystalline film from **1**. **d**, Fluorescence microscopic images of the large-area highly oriented crystalline film arrays with gap distance of 15 μm. **e**, AFM height image of the corresponding marked region in **c**. **f**, GIWAXS image of the mono-patchy highly oriented crystalline film from **1**. **g**, Detailed packing methods in highly oriented crystalline film photoresist from **1**. Scale bars, **c**, 100 μm, **d**, 500 μm.

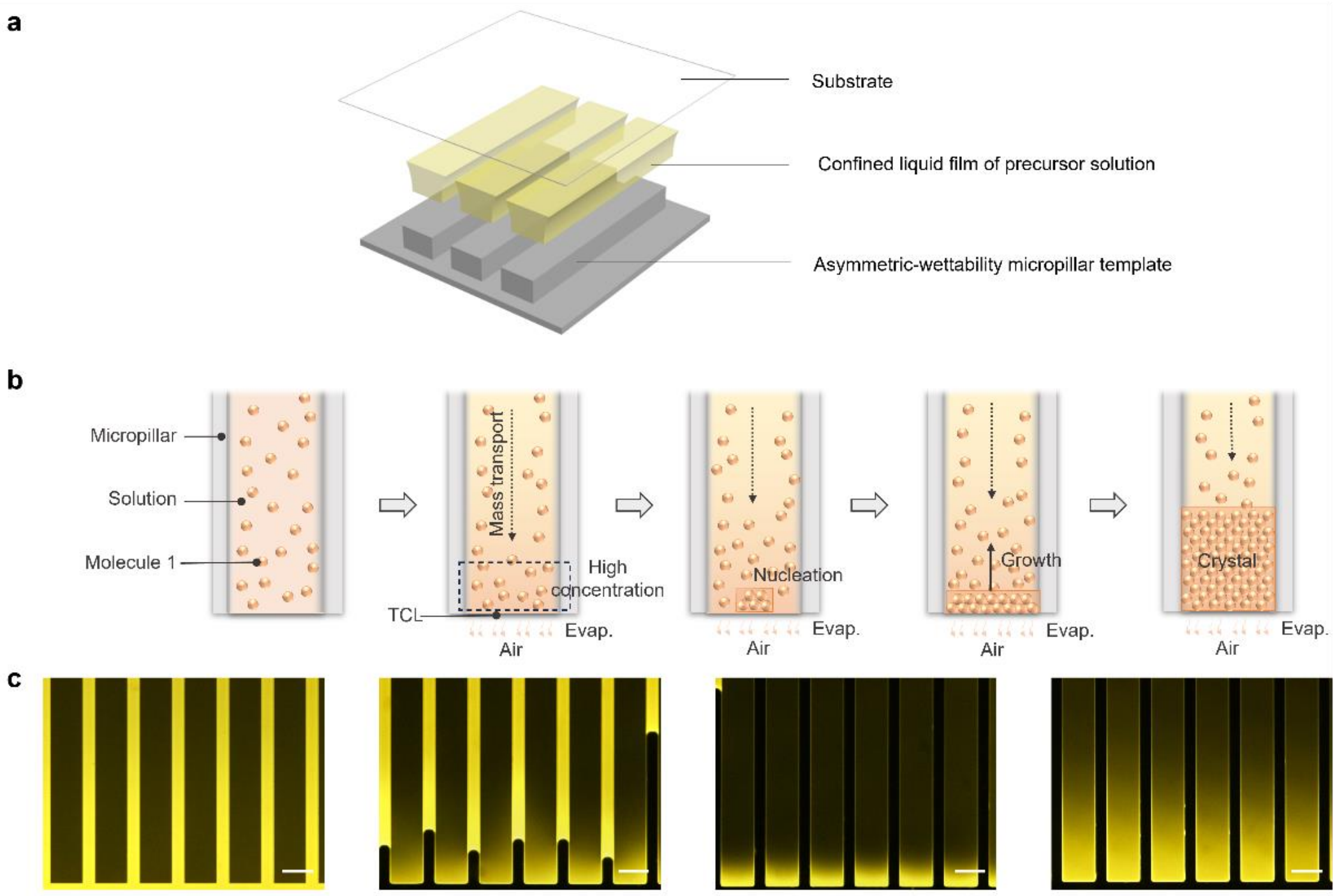

Supplementary Figure 5. The dewetting progress in a Sandwich-type structure using the confined capillary-bridge assembly method. a, The large-area highly oriented crystalline films were constructed through a "Sandwich-type" confined capillary-bridge assembly technique, which involves bridging a solution over asymmetric-wettability micropillar templates on various pre-cleaned substrates such as silicon, glass, and quartz. **b**, Schematic diagram illustrating concentration distribution changes during dual solid-liquid interface-confined assembly process. **c**, In-situ fluorescence microscope images of the dewetting process. Scale bar = 100 μm.

Reviewer 2 (R2):

Comments made by R2 and our responses are outlined below:

Comment 1: *A key concern is the mechanism underlying the solubility switch of Compound 1 films. Because no solubility change is observed in the absence of oxygen molecules—often implicated in radical processes—pathways other than radical cross-linking between neighbouring imidazole nitrogens may be operative. Could radical reactions in the solubilising hexyl chains (e.g., H-abstraction and subsequent cross-coupling) contribute, given that aliphatic sp3(C)–H bonds have lower bond-dissociation energies (BDEs) than aromatic N–H? In addition, the proposed oxidation of the benzimidazole ring to a bis-carboxylic acid (Fig. 2b) is difficult to accept without stronger literature support.*

Response 1: We thank the reviewer for their insightful suggestion. We acknowledge that hydrogen abstraction could potentially occur at the α-carbon position of the alkyl chains of the fluorene section. However, according to the crystal packing structure of the single-crystalline film photoresist, the distance between the benzimidazole radical sites that would form after hydrogen abstraction is approximately 13.69 Å (see figure below), which is too large to undergo cross-linking between neighbouring stacking molecules. Hydrogen abstraction at this position is more prone to lead to alkyl chain elimination and the formation of carboxyl-substituted species (as supported by literature). Both of these inferences are consistent with the changes observed in the infrared spectra of the polymerized products, the peaks rise at 1715 and 1215 cm^{-1} and diminishing at 2924 and 2854 cm^{-1}.

In contrast, we consider cross-linking at the benzimidazole sites to be more plausible based on several lines of evidence. Steady-state absorption spectroscopy indicates the presence of benzimidazole radical intermediates (Supplementary Figure 25), while IR and mass spectrometry analyses of the polymerized aggregates confirm the formation of abundant carboxylic acid (Figure 2k) and imidazole dicarboxylic fragments (Supplementary Figure 29). These observations align with the products expected from single- and double-oxidation processes of benzimidazole, as reported in literature (*Russian Journal of General Chemistry, 2018, 88, 5, 874-878*).

Based on the reviewer's valuable suggestion, we have re-examined the proposed mechanism. The intermolecular benzimidazole moieties between neighbouring stacking molecules distance of 4.135 Å, which suggests that direct C-C or N-N coupling remains spatially unfavourable. Therefore, we propose that benzimidazole radicals may first react with reactive oxygen species, particularly singlet oxygen, to form peroxyl intermediates, which subsequently couple with adjacent benzimidazole radicals to generate N-O-O-N bridges. This revised mechanism provides a spatially and chemically consistent explanation for the observed intermediates and products. Accordingly, we have modified the mechanistic illustration in Figures 2h, 3f, and Supplementary Figure 32, incorporated a description of possible oxidation at the alkyl positions, and added new relevant references as ref. 92 to the discussion section.

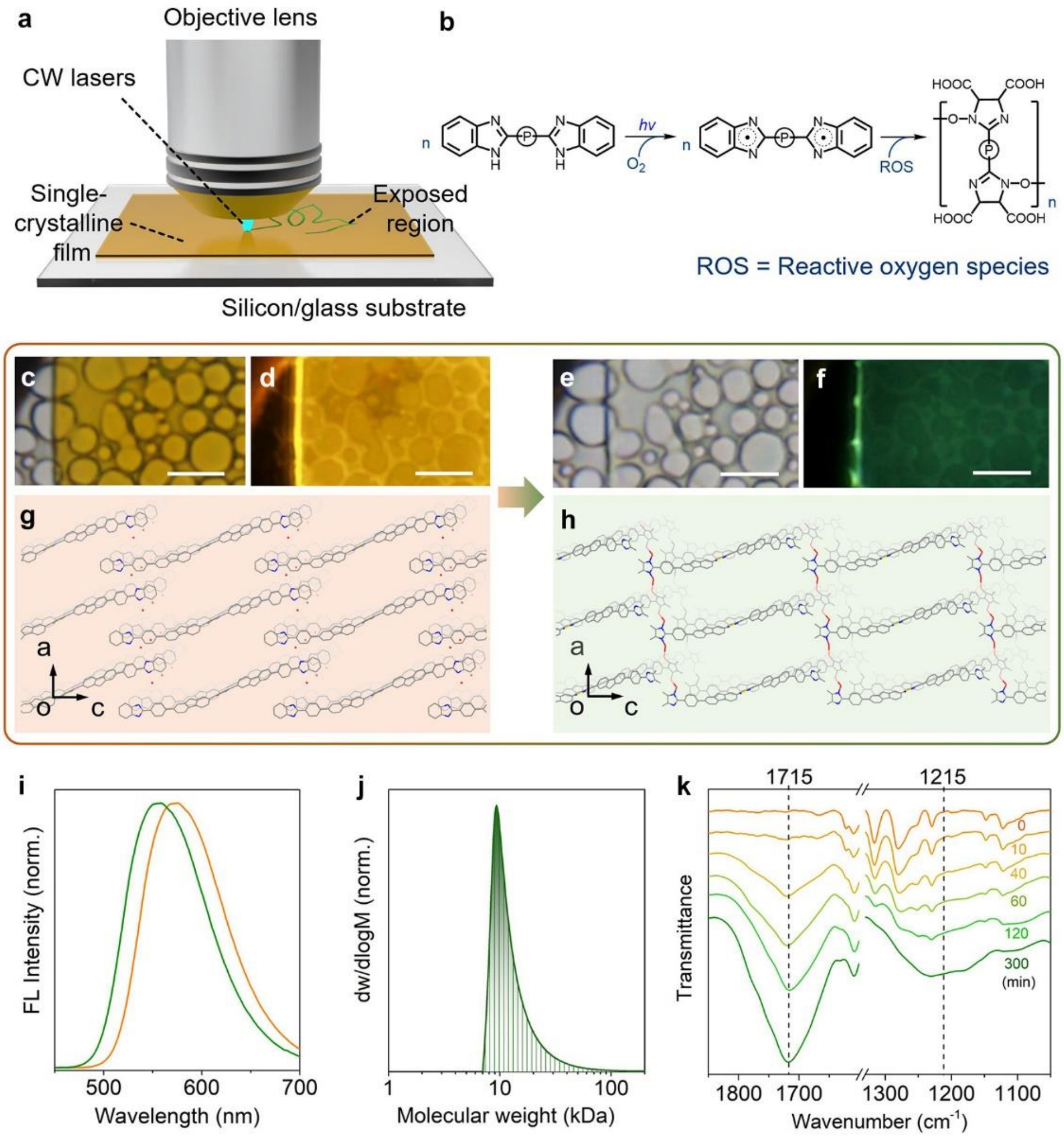

Figure 2. Photoinduced topochemical polymerization on large-area single crystalline film from 1. a. Cartoon illustration of spatial selective photoinduced topochemical polymerization on the surface of large-area single-crystalline film using low power CW visible lasers. **b**. Photoinduced topochemical polymerization occurred in the single-crystalline film from **1**. Optical (**c**) and fluorescent microscopic images (**d**) of single-crystalline film from 1 before light exposure. The circular patterns beneath the single-crystalline film arise from the carbon support film on the copper grid. Optical (**e**) and fluorescent microscopic images (**f**) of single-crystalline film from **1** after light exposure. simulated molecular packing of the cross-linked polymeric film. **g**, **h**. Molecular packing method of the single-crystalline film from **1** before (g) and (**h**) after light exposure. **i**. Fluorescent spectrum of the exposed (green) and unexposed (orange) single-crystalline film from **1**. **j**. Gel permeation chromatography (GPC) trance of the polymerized products in the light exposed region. **k**. *In-situ* infrared spectrum of the single-crystalline film from 1 under varied irradiation durations (470 nm, 50 μW).

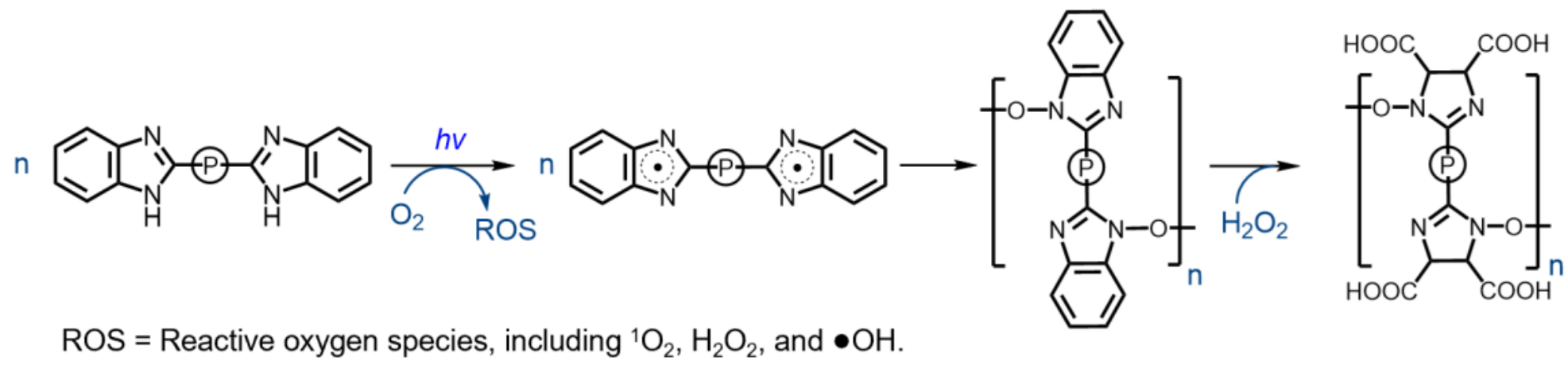

Supplementary Figure 32. Detailed photoinduced topochemical polymerization mechanism.

Comment 2: *On page 7, the text suggests that propanol may participate in intermolecular cross-linking. Has propanol been detected in the bulk by single-crystal X-ray analysis, or otherwise verified spectroscopically?*

Response 2: We leveraged single-crystal X-ray analysis confirms that propanol molecules participate in the crystal packing through continuous hydrogen-bond linkages between adjacent structural units, with a distance of 1.9 Å from the benzimidazole group, as shown in Figure 1g (figure below).

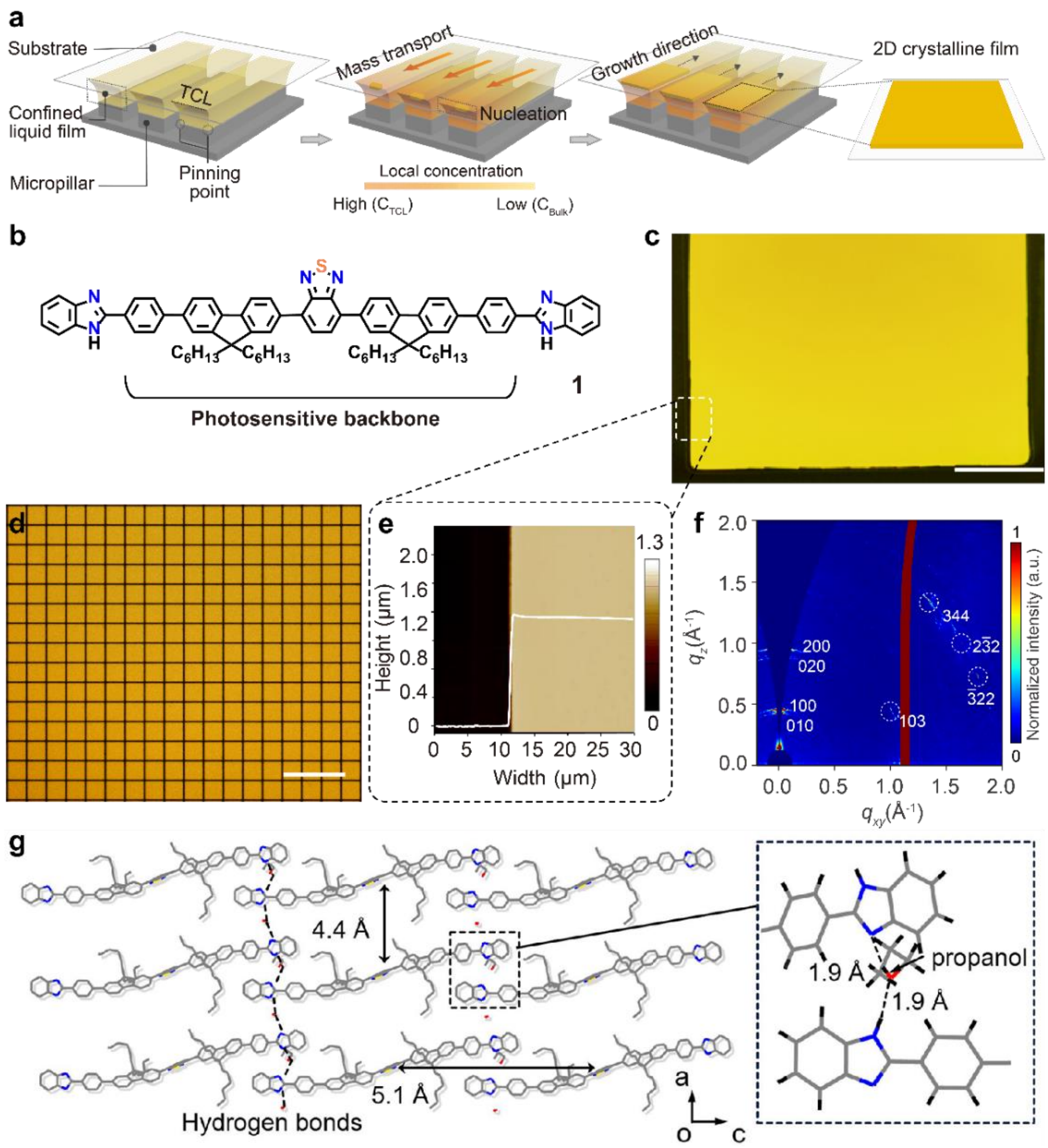

Fig. 1 | Preparation of large-area single-crystalline film photoresists. a, Cartoon illustration of the preparation of large area single crystalline film through dual solid-liquid interface-confined assembly method. **b**, Molecular structure of monomer **1**. **c**, Fluorescence microscopic image of the large-area mono-patchy single-crystalline film from **1**. **d**, Fluorescence microscopic images of the large-area single-crystalline film arrays with gap distance of 15 µm. **e**, AFM height image of the corresponding marked region in **c**. **f**, GIWAXS image of the mono-patchy single-crystalline film from **1**. **g**, Detailed packing methods in single-crystalline film photoresist from **1**. Scale bars = **c**, 100 µm, **d**, 500 µm.

Comment 3: *On page 4, the statement "Photosensitive monomers comprising imidazole moieties were designed for photoinduced topochemical polymerization and preferential pre-arrangement in the form of crystalline film" lacks citations. Please substantiate this design rationale with appropriate literature precedents.*

Response 3: We thank the reviewer for the comment. We have added literature references as refs 61-62 supporting the design rationale that photosensitive monomers comprising imidazole moieties can undergo photoinduced topochemical polymerization and preferentially pre-arrange as single-crystalline films (*2022, J. Am. Chem. Soc.; 2023, J. Am. Chem. Soc*.) in the revised manuscript.

Comment 4: *No image for LER extraction is provided, and the analysis conditions and software are not described.*

Response 4: We thank the reviewer for pointing out this issue. We have now included the SEM image used for LER extraction and clarified that the LER quantitative data for the photocarving structures were obtained using the ProSEM software. We have also added the corresponding description in the analysis section of the Supporting Information.

"The line edge roughness (LER) of the fabricated micro/nanostructure edges was analyzed using ProSEM V3.0. After inputting the scale bar information, a region of interest (ROI) was selected for edge detection to obtain the roughness data. The feature type was set to *Line and Spaces*, the polarity to *Line/Pillar*, the position to *mid*, and the analysis method to *sigmoidal signal*."

Comment 5: *For relevance to integrated-circuit photolithography, demonstration of dense patterns (1:1 line/space) is needed rather than isolated lines. Cross-section images of densely patterned lines are strongly recommended.*

Response 5: We thank the reviewer for the suggestion. As the reviewer suggested, we have incorporated the densely patterned photocarved lines with width resolution of 63 nm and 96 nm, with corresponding LER of 3.5 nm and 2.4 nm. We also incorporated the cross-section image of the photocarved lines pattern. The new data have been included as Supplementary Figs. 19-20 in the revised Supporting Information.

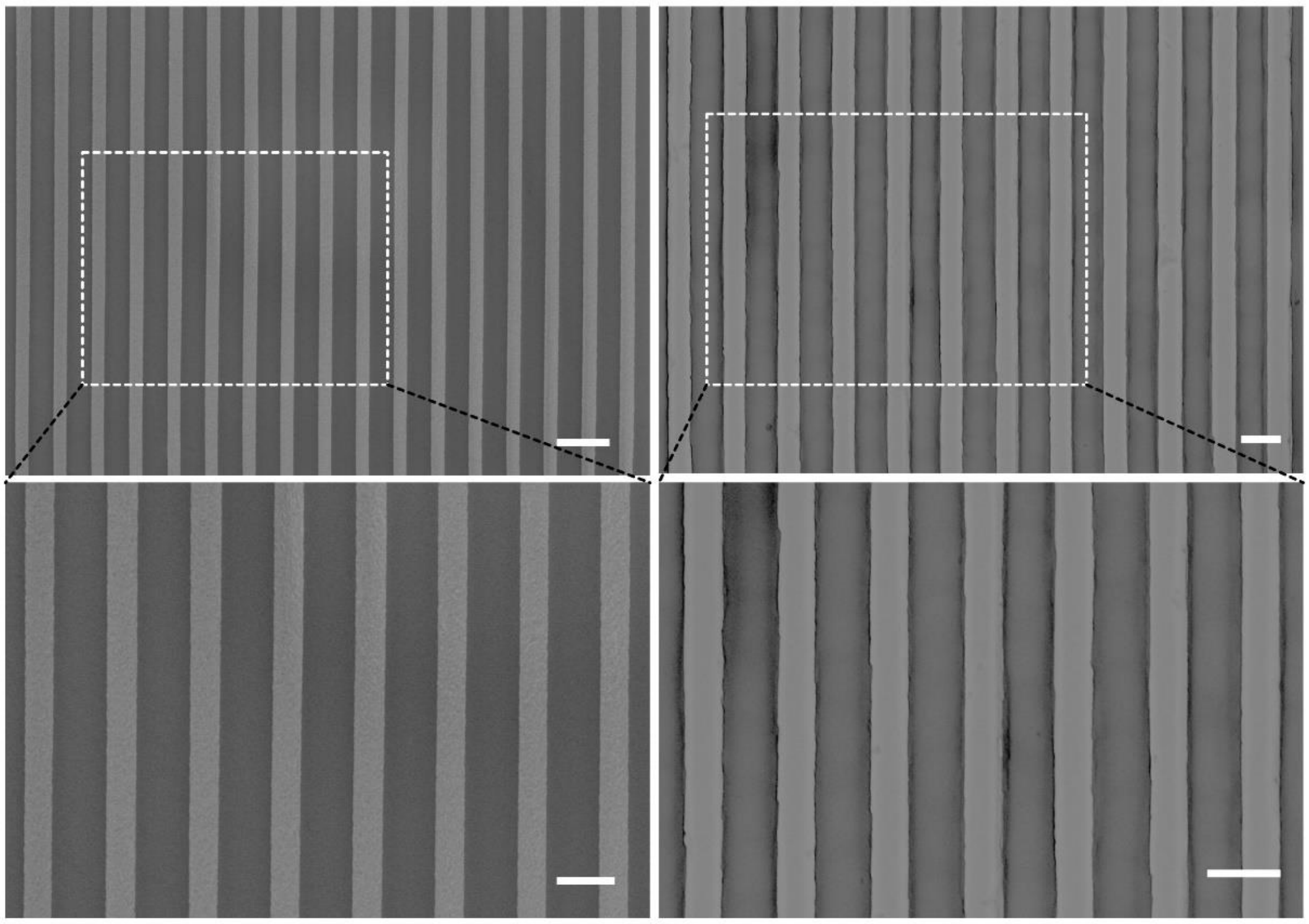

Supplementary Figure 19. **Preparation of photocarved dense line patterns.** Bright-field SEM images of photocarved 1:1 line-to-space dense line patterns on films from **1**, showing **a**, a resolution of 96 nm and a line-edge roughness (LER) of 2.4 nm, and **b**, a resolution of 63 nm and an LER of 3.5 nm. Scale bar = 400 nm (**a**), 200 nm (**a**, magnified), and 100 nm (**b**).

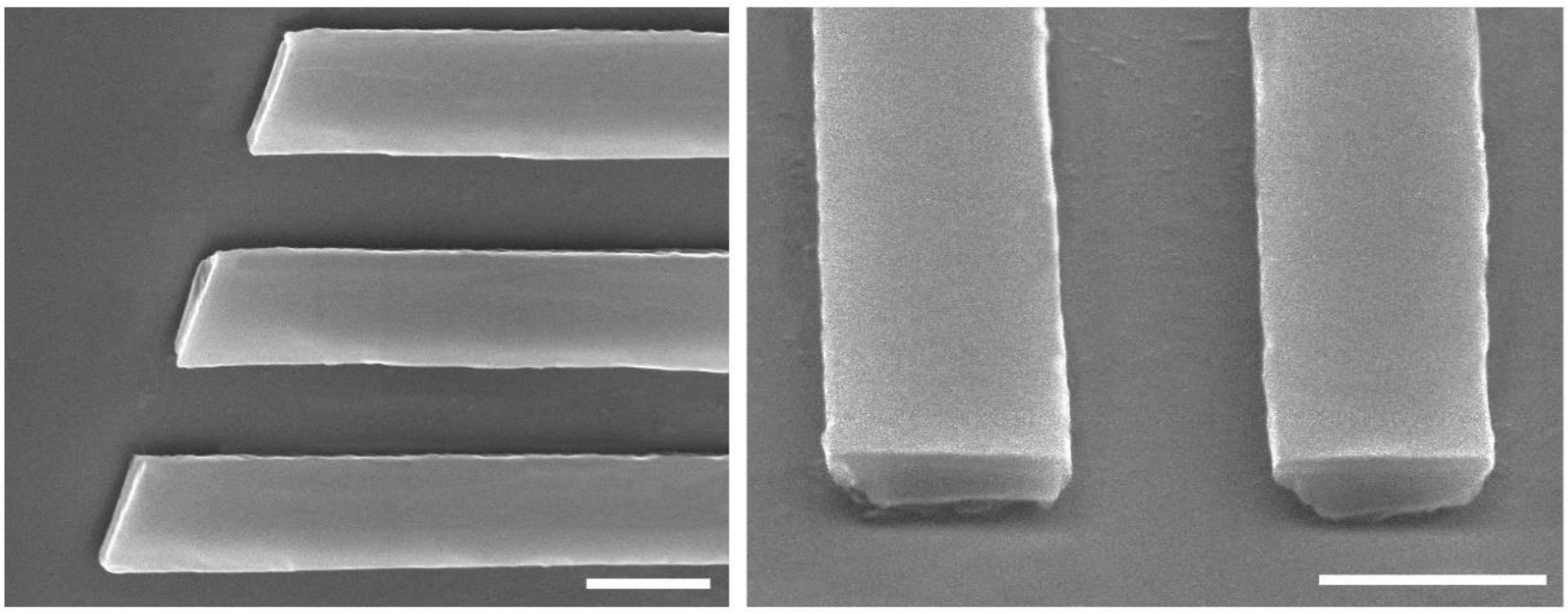

Supplementary Figure 20. **Cross-sectional morphology of photocarved line patterns. a-b**, Bright-field SEM images of the cross sections of photocarved line patterns on films of **1**. Scale bar = 200 nm.

Comment 6: *Can the LER of line patterns be quantitatively compared for patterns formed on different crystal growth faces?*

Response 6: We thank the reviewer for the insightful comment. To compare the LER of patterns formed on different crystal growth faces, we prepared grid-shaped patterns under identical laser beam conditions (480 nm, 7.6 µW, 50 ms). The horizontal lines correspond to

the (100) crystal face, while the vertical lines correspond to the (001) face. After development, we found that the LER values along both the (100) and (001) faces were approximately 3 nm, with the difference falling within the experimental error (as shown in the figure below). Based on these results, we conclude that the influence of crystal face orientation on LER is minimal. We have added the corresponding data and descriptions to the revised manuscript and Supporting Information.

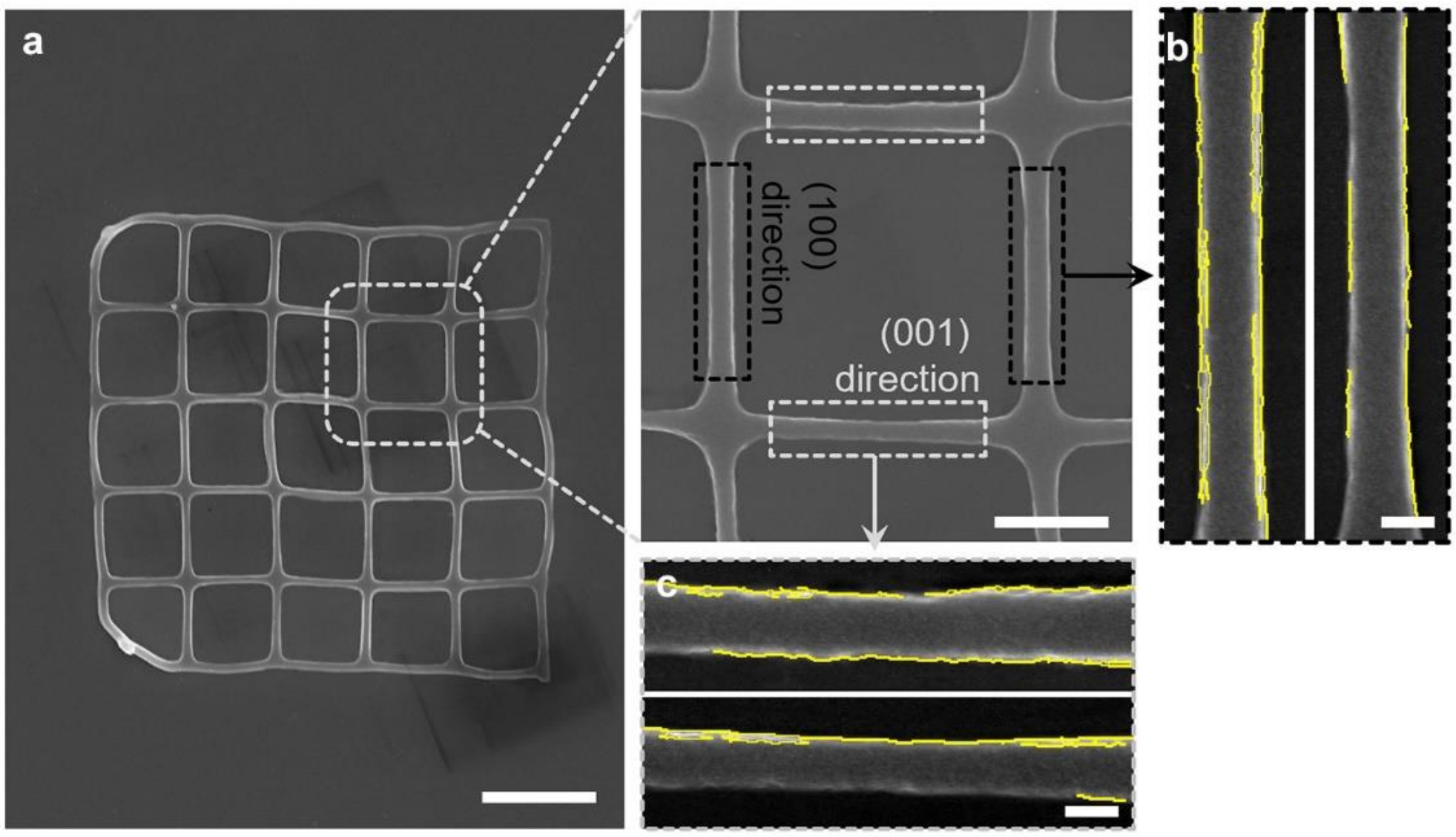

Supplementary Figure 21. Comparison of line-edge roughness (LER) along different facet directions. a, SEM images and enlarged view of photocarved square-pore grid patterns with orthogonal lines, align with the (001) and (100) facet directions. Scale bars = 1 μm and 500 nm (insets). **b**, Quantitative analysis of LER along the (100) direction, yielding values of 3.97 and 2.62 nm (black-dotted region). Scale bar = 100 nm. **c,** Quantitative analysis of LER along the (001) direction, yielding values of 3.46 and 2.94 nm (grey-dotted region). Scale bar = 100 nm.

Comment 7: *The developer solutions listed on page 7 appear to differ from those in Supporting Information. Please reconcile these discrepancies.*

Response 7: We thank the reviewer for pointing out this point. We have unified the related descriptions. The corrected formulations are as follows: for the positive-mode solvent mixture developer, acetonitrile to DMSO = 3:1 (v/v); and for the negative-mode solvent mixture developer, chloroform to methanol to n-hexane = 1:3:20 (v/v/v). We corrected the corresponding descriptions in the revised manuscript.

"Specifically, a developer solution of acetonitrile and DMSO (volume ratio = 3:1) selectively dissolves the polymeric products, whereas a 1:3:20 volume ratio of chloroform, methanol, and n-hexane preserves them."

Reviewer 3 (R3):

Revisions suggested by R3 and our responses are outlined below:

Comment 1: *Some recent reviews on topochemical polymerization (eg. Chem. Sci. 2021, 12, 5361) is missing in the reference list*

Response 1: We thank the reviewer for the comment. We have incorporated the suggested recent review as ref. 20 (*Chem. Sci. 2021, 12, 5361*) to the reference list in the revised manuscript.

Comment 2: *Tacticity refers to the relative stereochemical arrangement of pendant groups. I don't see a group that can generate tacticity in the polymer mentioned here. Authors may clarify what they meant by isotactic?*

Response 2: We have revised the expressions to "uniform molecular orientation" in the revised manuscript.

Comment 3: *Authors cited 2D porous frame work in refs 7-10. Two recent studies on 2D polymers synthesis by topochemical polymerization (Nat. Commun. 2024, 15, 6638 and JACS 2025, 147, 37798) is missing in this list.*

Response 3: We thank the reviewer for the helpful comment. We have added the suggested recent studies on 2D polymer synthesis by topochemical polymerization as refs. 11-12 (*Nat. Commun. 2024, 15, 6638; J. Am. Chem. Soc. 2025, 147, 37798*) to the reference list in the revised manuscript.

Comment 4: *GIWAXs suggest crystallinity. How the authors know the film is single-crystalline in nature? What proof authors have to rule out multiple platelets? For example, how do they rule out minute cracks A? Authors state that "Successful topochemical addition reactions have been reported in crystal lattices involving diverse reactive groups, including alkynes/azide4,18,19, alkene/azides20, olefins21-23, diynes17,24,25, triynes26-28, dienes29-31, trienes29, para-quinodimethanes32-36 and bis(indanone)s14,37,38". In this context, the recent examples of polymerisation via Topochemical Diels-Alder Cycloaddition -TDAC (JACS 2024, 146, 30495) and Topochemical alkyne Nitril-oxide cycloaddition -TANOC (Angew. Chem. Int. Ed., 2025, 64, e202520947) may be included.*

Response 4: We thank the reviewer for these insightful comments regarding the crystalline nature of the films and the broader context of topochemical reactions.

With respect to the assignment of single crystallinity, we agree that grazing-incidence wide-angle X-ray scattering (GIWAXS) primarily probes long-range order and, on its own, does not unambiguously establish single-crystal character. Accordingly, the revised manuscript clarifies that this assignment is based on a combination of complementary structural and morphological analyses.

Transmission electron microscopy (TEM) performed at randomly selected positions across the film, together with selected-area electron diffraction (SAED), reveals a highly consistent

crystallographic orientation within individual domains, with no discernible grain boundaries typically associated with polycrystalline assemblies. Consistent with these observations, the GIWAXS patterns (Figure 1f) exhibit a single set of sharp, well-defined diffraction features rather than Debye-Scherrer rings, indicating the absence of orientational averaging from multiple misoriented crystallites within the probed area. The lack of additional diffraction features indicates the unlikely coexistence of multiple crystalline platelets with differing orientations. In parallel, high-resolution atomic force microscopy (AFM) shows a continuous film morphology without observable cracks or inter-platelet boundaries over lateral length scales of approximately 20 μm. Surface roughness values measured across ten randomly selected regions (each covering ~25 μm^2) fall in the range of 0.05-0.27 nm, consistent with a crystalline-grade flat surface. The corresponding data and discussion have been added to Supplementary Figs. 11-12 in the revise supplementary information.

Taken together, the highly uniform GIWAXS diffraction features, supported by real-space imaging, indicate that the films exhibit single-domain, single-crystal-like behaviour within the experimental resolution and the probed regions. To avoid overstatement, the revised manuscript now explicitly frames this conclusion in terms of local single-crystal characteristics rather than asserting complete single crystallinity at the macroscopic scale. We note that, as with any structural characterisation, the presence of structural features below the instrumental resolution cannot be categorically excluded. However, within the spatial resolution and sampling statistics of the combined TEM/SAED, GIWAXS, and AFM measurements, no evidence of grain boundaries, orientational disorder, or subdomain formation is observed. The conclusions are therefore appropriately framed in terms of the probed regions and experimental resolution. We have revised the corresponding expression in the revise manuscript.

"Within the spatial resolution and sampling statistics of combined selected-area electron diffraction (SAED) and atomic force microscopy (AFM) measurements, no grain boundaries, orientational disorder, or subdomain formation were detected (Supplementary figs. 11-12), confirming the local single-crystal characteristics."

Regarding the literature context, we appreciate reviewer's suggestion. The manuscript has been revised to include recent examples of polymerization via topochemical Diels-Alder cycloaddition (TDAC) and topochemical alkyne nitrile-oxide cycloaddition (TANOC) as refs 40-41 in the revised manuscript.

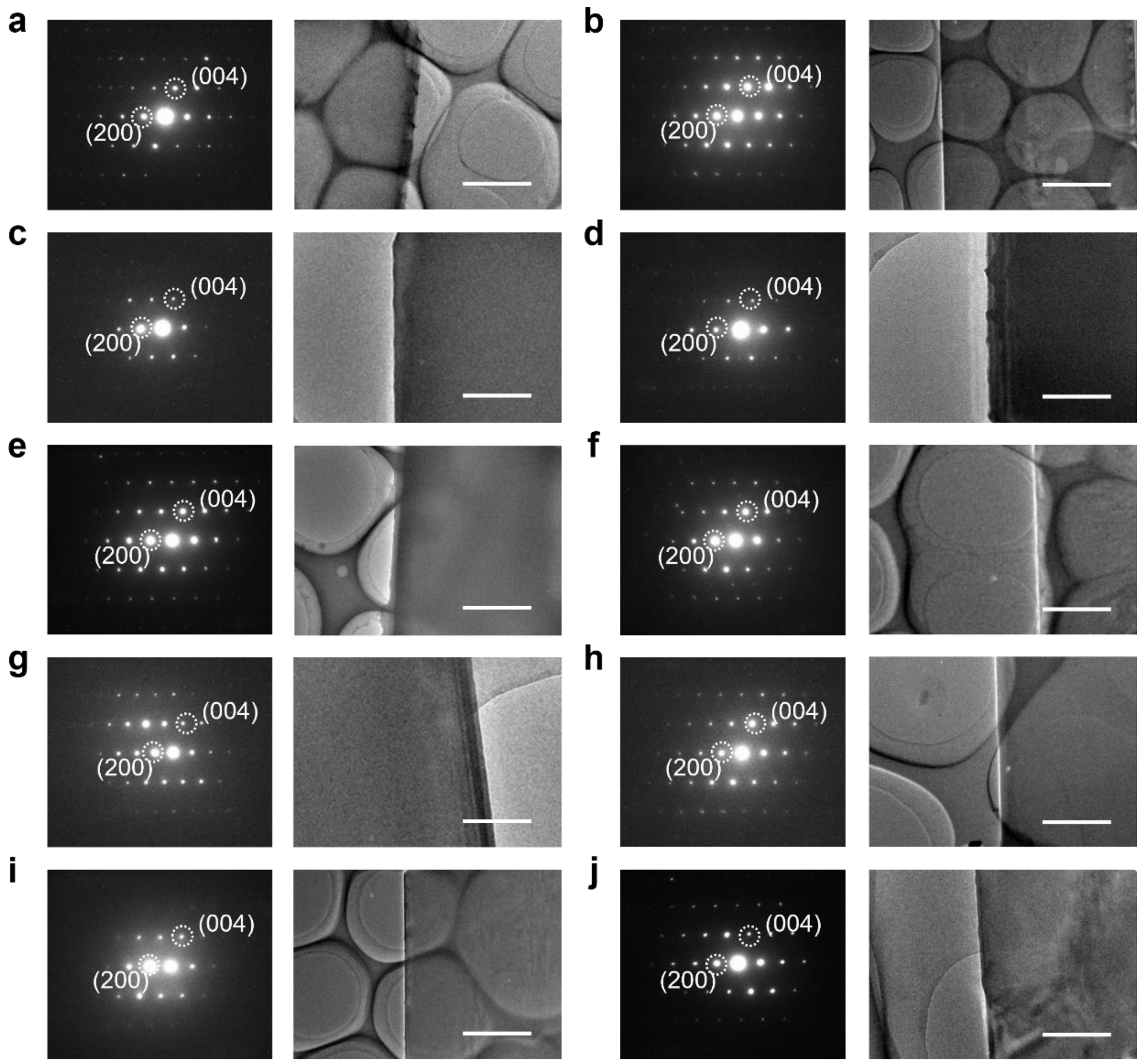

Supplementary Figure 11. Uniform crystallographic orientation revealed by TEM and SAED. TEM images obtained from randomly selected regions of the film, along with the corresponding SAED patterns, indicate a high degree of crystallographic orientation uniformity within individual domains, without observable grain boundaries associated with polycrystalline structures. Scale bar = 1 μm. The circular patches beneath the single-crystalline film of **1** in (**a-j**) originate from the supporting carbon film on the TEM copper grid.

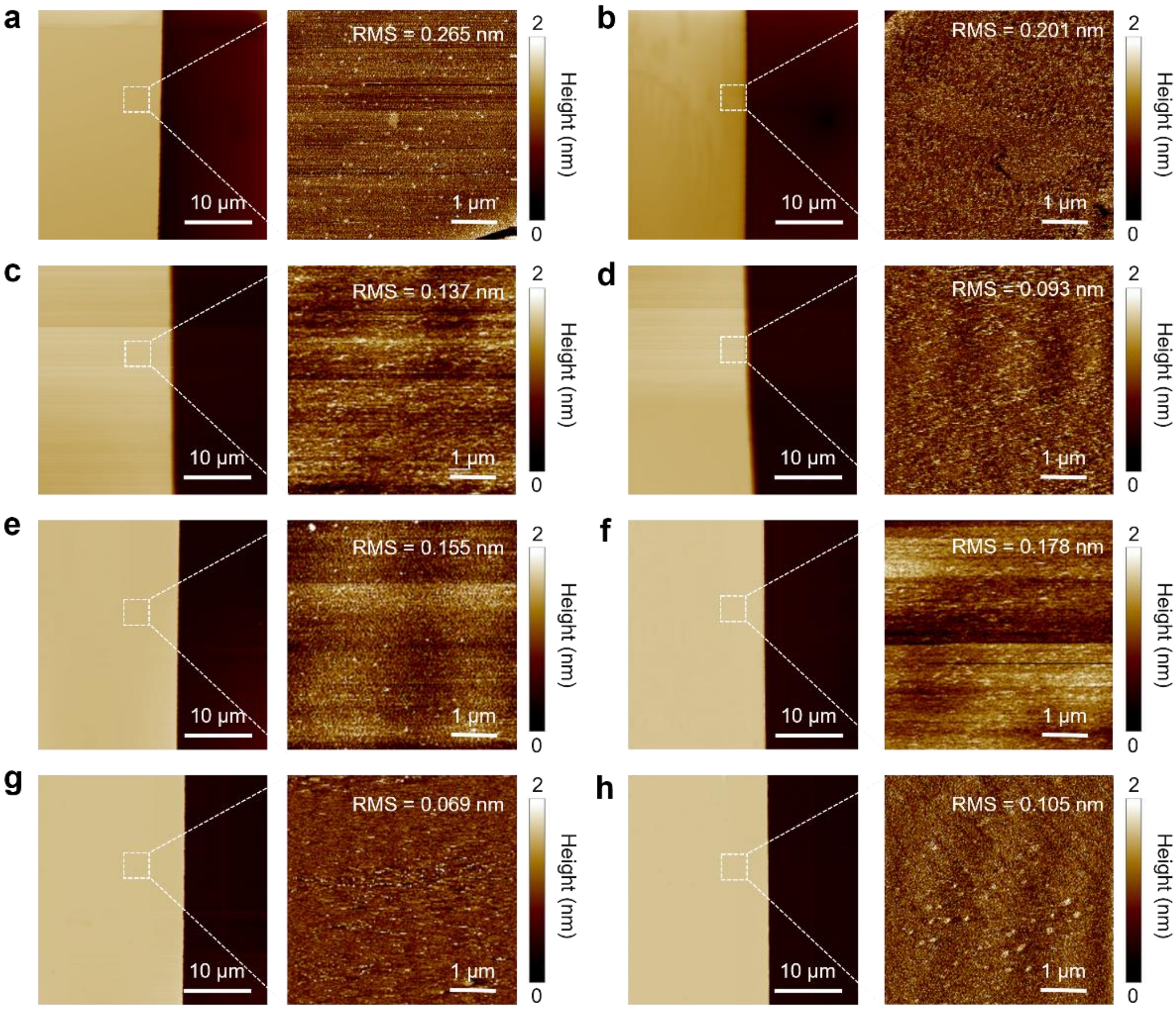

Supplementary Figure 12. Continuous morphology and surface flatness revealed by AFM. High-resolution AFM images obtained from multiple regions of the film reveal a continuous morphology without discernible cracks or inter-platelet boundaries over lateral length scales of ~20 μm. The surface roughness measured across ten randomly selected areas (~25 μm^2 each) falls within 0.05–0.27 nm, indicating a high degree of surface flatness.

Comment 5: *Authors stated that "The single-crystalline nature of the mono-patch film from 1 was confirmed through well-defined diffraction peaks, the absence of diffraction rings, and consistent lattice parameters obtained from grazing-incidence wide-angle X-ray scattering (GIWAXS) measurements". The absence of diffraction ring means that the sample is NOT crystalline! Authors may clarify this.*

Response 5: We thank the reviewer for pointing out this ambiguity. We agree that the original wording was imprecise and could lead to misunderstanding. The absence of diffraction rings does not imply a lack of crystallinity; rather, in grazing-incidence geometries, it indicates the absence of randomly oriented crystallites within the probed area.

Following additional measurements, the weak semicircular feature at $q_z \approx 0.35$ $Å^{-1}$ in the original Fig. 1f was observed only at selected incident angles and was not reproducible under all grazing-incidence conditions. Diffraction rings associated with randomly oriented crystallites are generally expected to persist across different incident angles. The strong incident-angle dependence of this feature therefore argues against a polycrystalline origin. We have revised GIWAXS data in Fig. 1f in the revised manuscript.

In the revised manuscript, we have clarified this point and corrected the wording accordingly. Specifically, the description has been revised to state that the presence of well-defined, discrete diffraction features-together with the absence of Debye-Scherrer rings-reflects a high degree of orientational order and the lack of polycrystalline averaging, consistent with a single-domain or highly oriented crystalline film. We have avoided attributing crystallinity solely to the absence of diffraction rings and instead emphasized the combined evidence from the discrete diffraction peaks and their consistent lattice parameters.

These clarifications have been incorporated into the main text and the associated discussion of the GIWAXS data.

"The crystalline film from **1** exhibits a high degree of crystallographic order, as indicated by discrete GIWAXS diffraction features (Fig. 1f) and consistent lattice parameters. The absence of Debye-Scherrer rings and unidirectional polarized fluorescence emission (Supplementary Fig. 10) suggests the lack of randomly oriented crystallites within the probed area, consistent with a single-domain or highly oriented crystalline film."

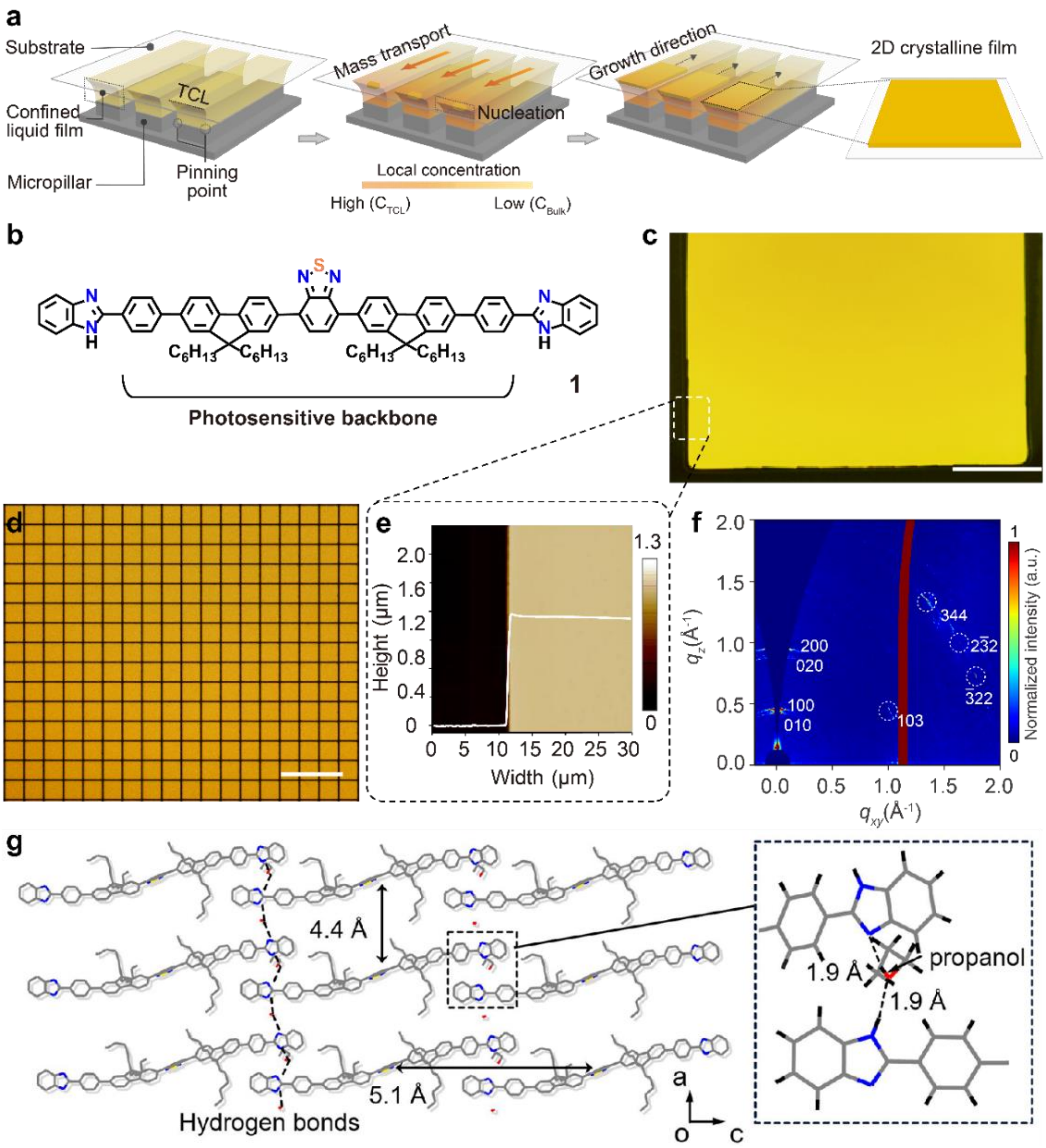

Fig. 1 | Preparation of large-area single-crystalline film photoresists. a, Cartoon illustration of the preparation of large area single crystalline film through dual solid-liquid interface-

confined assembly method. **b**, Molecular structure of monomer **1**. **c**, Fluorescence microscopic image of the large-area mono-patchy single-crystalline film from **1**. **d**, Fluorescence microscopic images of the large-area single-crystalline film arrays with gap distance of 15 µm. **e**, AFM height image of the corresponding marked region in **c**. **f**, GIWAXS image of the mono-patchy single-crystalline film from **1**. **g**, Detailed packing methods in single-crystalline film photoresist from **1**. Scale bars = **c**, 100 µm, **d**, 500 µm.

Comment 6: *Authors state that "Common crystal forms-such as plate-like, rod-like, or sheet-like structures-are prone to stress accumulation during conformational changes in topochemical reactions, often resulting in fracture or cracking." A reference for this statement is essential (JACS 2024, 146, 27100).*

Response 6: We thank the reviewer for pointing this out. We have added the suggested reference as ref. 44 (*J. Am. Chem. Soc. 2024, 146, 27100*) in the revised manuscript to support the statement regarding stress accumulation and fracture in common crystal forms during topochemical reactions.

Comment 7: *What happens to the solvent (propanol) which was hydrogen bonded to the imidazole unit, upon polymerization?*

Response 7: We thank the reviewer for the comment. During the polymerization process, hydrogen abstraction disrupts the hydrogen bonds, leading to the escape of propanol. Such solvent loss after hydrogen-bond disruption has also been observed in elevated-temperature experiments (*J. Am. Chem. Soc., 2023, 145, 17, 9771*). In response to the reviewer's suggestion, we have added the relevant description to the revised manuscript.

"The photoinduced polymerization induces structural changes of molecule **1** during the reaction, leading to the disruption of the bridging hydrogen bonds between propanol and **1**. As a result, the hydrogen-bonded bridging propanol molecules are released into the environment as free, individual propanol molecules."

Comment 8: *What are the circular patches in Fig 2 fluorescent microscopic images, apparently with grain boundaries? Does it say that the film is not a continuous crystal?*

Response 8: We thank the reviewer for the comment. The circular patches in the background of Figure 2 originate from the supportive carbon film on the TEM copper grid. To avoid any misunderstanding, we have incorporated the related descriptions to both the revised main text and the Supplementary Information.

"The circular patches beneath the single-crystalline film of **1** originate from the supporting carbon film on the TEM copper grid."

Comment 9: *Same issue is with supplementary Fig 11.*

Response 9: We have added the corresponding explanation to the supplementary figure in the revised supplementary information.

Comment 10: *In figure 2H, the crystal packing of the polymer, the N-N single-bond distance between two imidazole rings seems to be unusually too long.*

Response 10: We thank the reviewer for pointing out this important issue. Before the reaction, the distance between benzimidazole units bridged by propanol is 4.135 Å. After polymerization, it decreases by ca. 20% to 3.3 Å, which is still too long for direct bond formation. We have modified the mechanism discussion part. We proposed that benzimidazole radicals first react with reactive oxygen species, especially singlet oxygen, to form peroxyl intermediates. These intermediates then couple with neighbouring benzimidazole radicals to form N–O–O–N bridges. This mechanism explains the observed intermediates and products in a consistent way. We have updated the mechanistic illustrations in Figures 2b, 2h, 3f, and Supplementary Figure 32, added a description of possible oxidation at the alkyl positions, and included new relevant references in the discussion section.

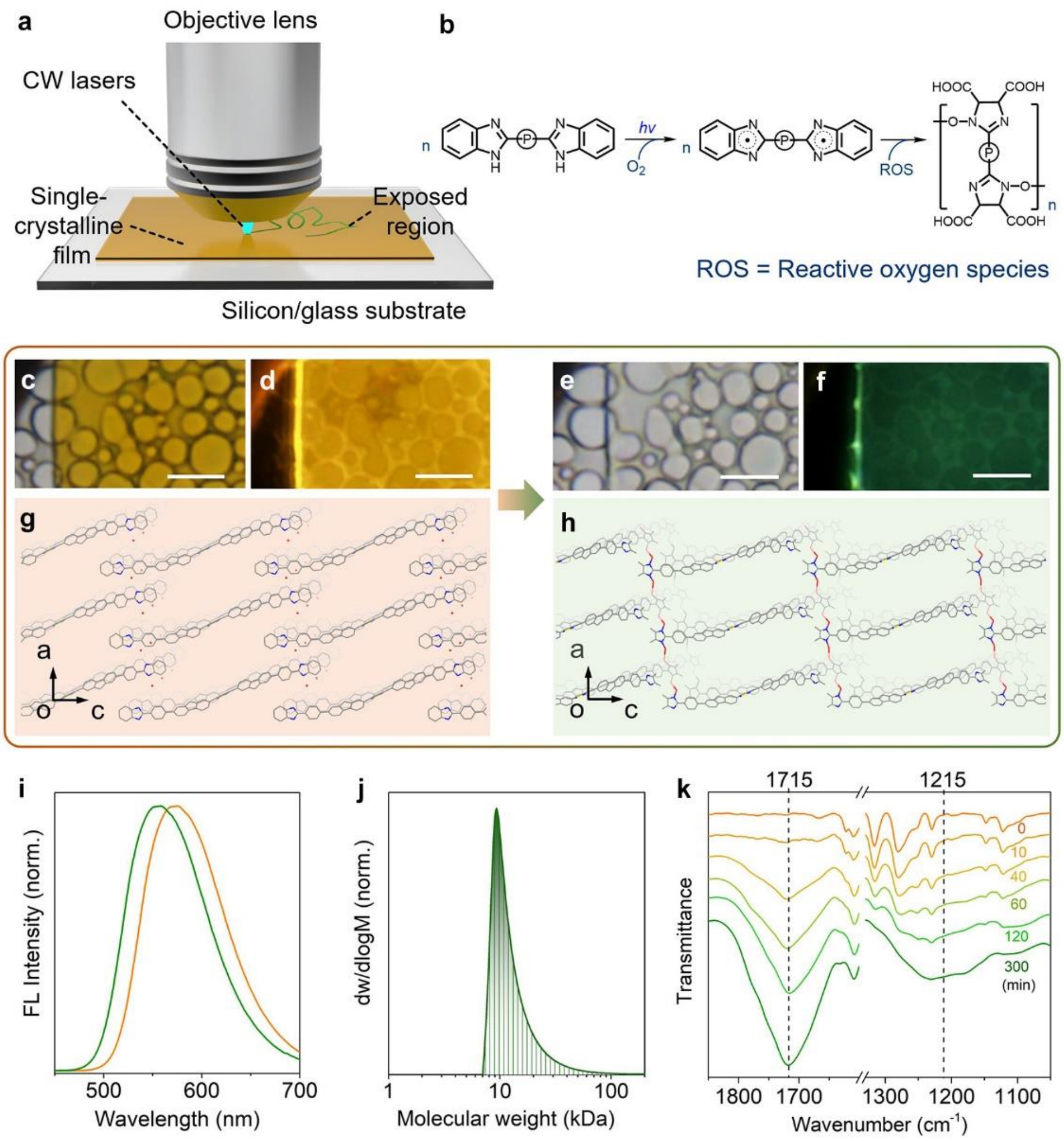

Fig. 2 | Photoinduced topochemical polymerization on large-area single crystalline film from 1. a. Cartoon illustration of spatial selective photoinduced topochemical polymerization on the surface of large-area single-crystalline film using low power CW visible lasers. **b**. Photoinduced topochemical polymerization occurred in the single-crystalline film from 1.

Optical (**c**) and fluorescent microscopic images (**d**) of single-crystalline film from 1 before light exposure. The circular patches beneath the single-crystalline film of 1 originate from the supporting carbon film on the TEM copper grid. Optical (**e**) and fluorescent microscopic images (**f**) of single-crystalline film from 1 after light exposure. simulated molecular packing of the cross-linked polymeric film. g, h. Molecular packing method of the single-crystalline film from **1** (**g**) before and (**h**) after light exposure. **i**. Fluorescent spectrum of the exposed (green) and unexposed (orange) single-crystalline film from **1**. **j**. Gel permeation chromatography (GPC) trance of the polymerized products in the light exposed region. **k**. *In-situ* infrared spectrum of the single-crystalline film from **1** under varied irradiation durations (470 nm, 50 μW).

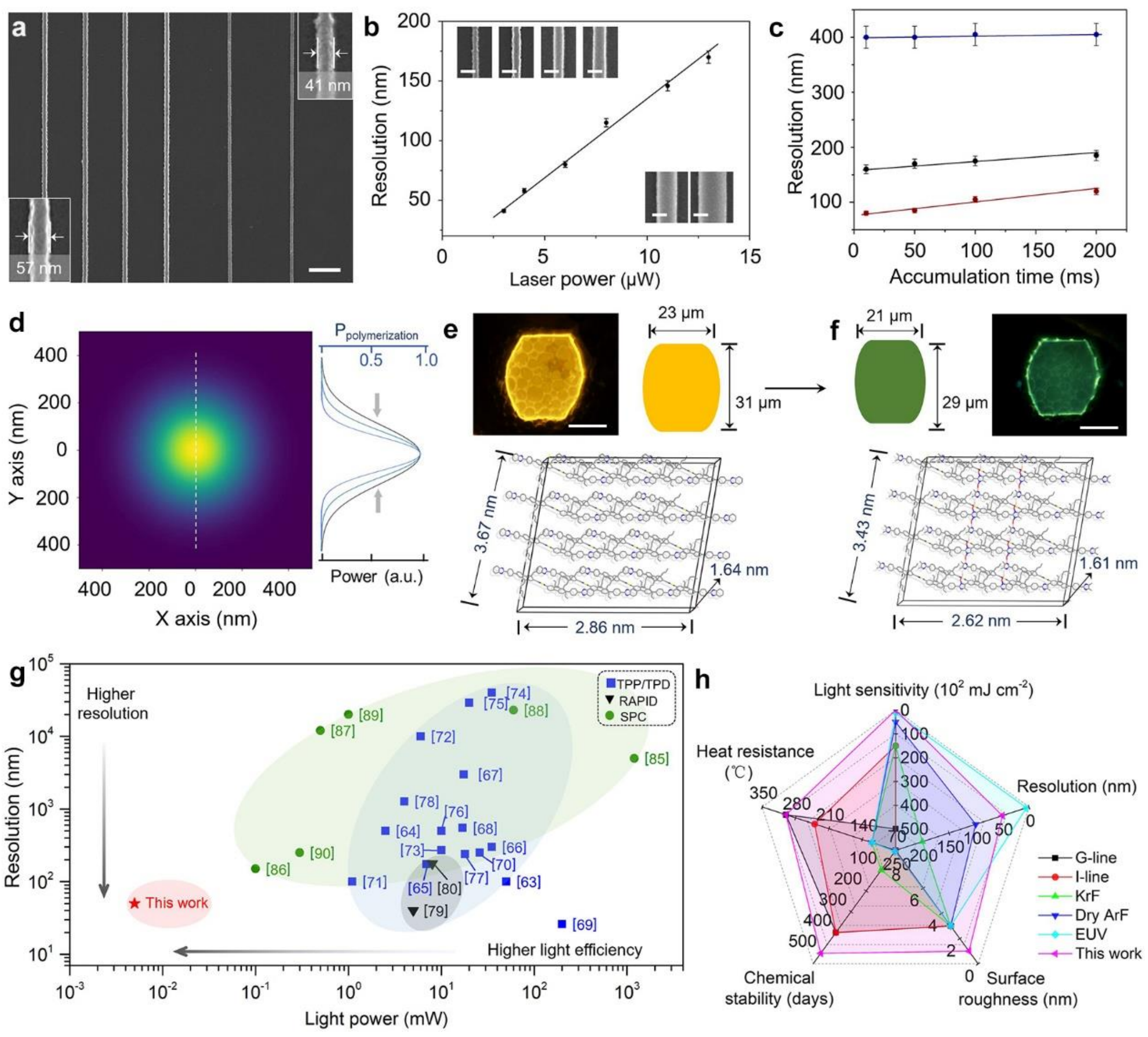

Fig. 3 | Topochemical polymerization-based photocarving performances. a. SEM image of the negative mode photocarving linear array on silicon substrate after development. The inset is the representative nanowires with the feature line width of 57 and 41 nm. **b**. The correlation between the photocarving feature width as a function of applied CW laser power. **c**. The correlation between the photocarving feature width as a function of irradiation durations. **d**. The theoretical calculated effective topochemical polymerization domain to achieve polymers surpass the threshold D_p (k = 5, 10, and 20) within a laser spot possessing a Gaussian power distribution. **e-f**. The microscopic and macroscopic dimensional variations of single-crystalline film from **1** before and after light exposure. **g**. Comparison of the state-of-the-art photocarving

performance among organic photoresists. **h**. Comparison of the photocarving performances among the existing photolithographic modalities. Scale bars, **a**, 400 nm, **e**, **f**, 20 μm.

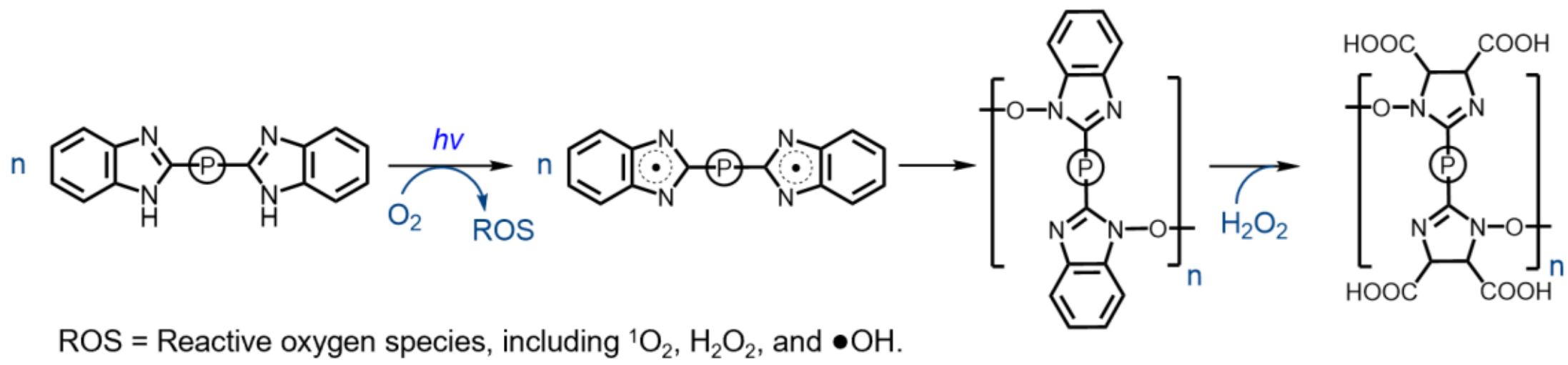

Supplementary Figure 32. Detailed photoinduced topochemical polymerization mechanism.

"The formation of an acidic polymeric products and cross-linked bridging -O-O- bonds introduce sufficient polarity differences compared to the unpolymerized monomers, facilitating selective removal of the polymeric products upon irradiation."

Comment 11: *The absorption spectra shows that there is no absorption after 450 nm. Then how the irradiation using 488 nm effect the reaction?*

Response 11: We thank the reviewer for the comment. The absence of a specific absorption spectrum in the original text may have been misleading. As shown in the UV–vis spectra (figure below), the single-crystalline thin-film photoresist exhibits significant absorption at 488 nm. We have added this absorption spectrum as Supplementary Fig. 13 in the revised Supplementary Information.

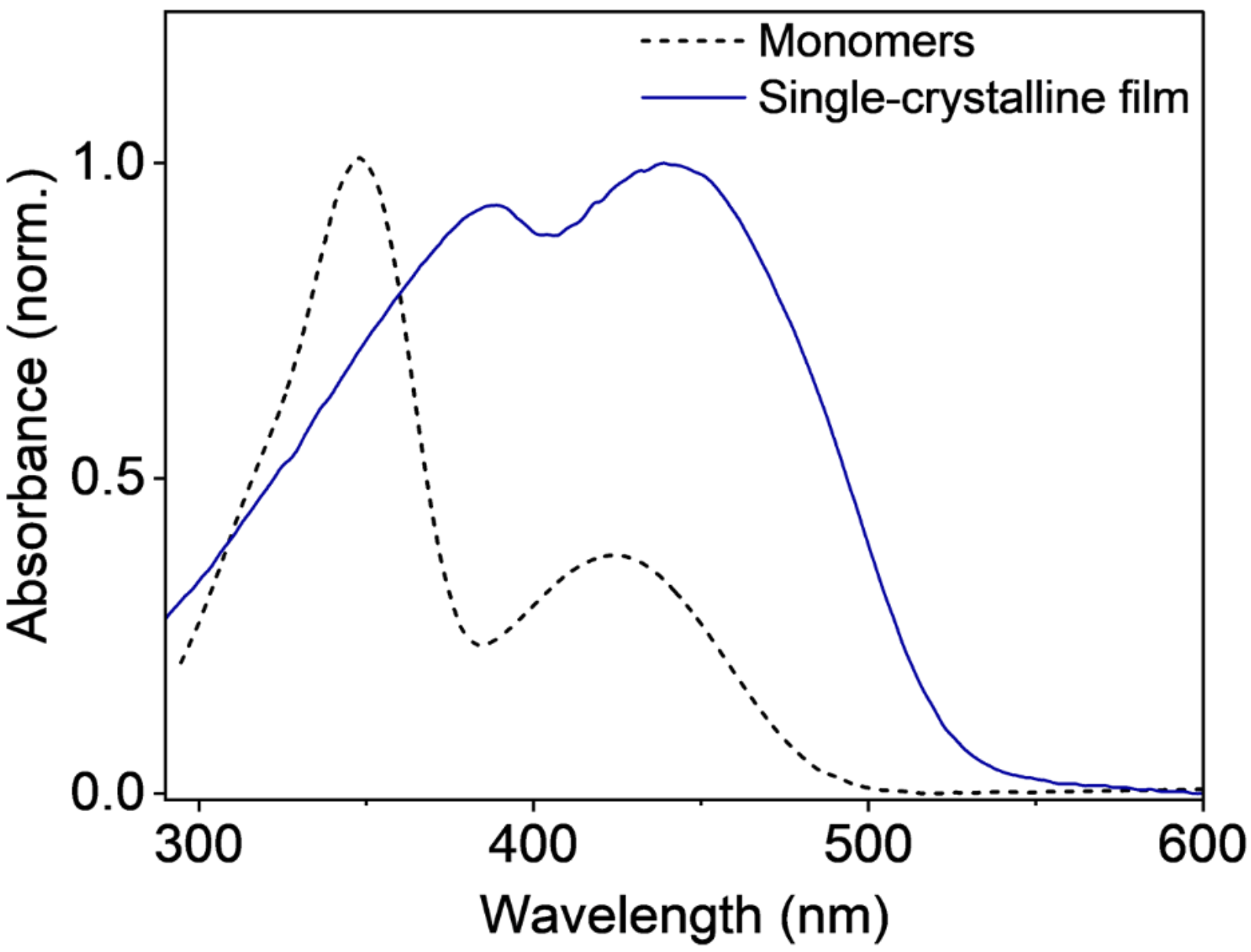

Supplementary Figure 13. UV-vis absorption of monomer solution in THF (black dashed line) and single-crystalline film (blue solid line) from **1**.

Comment 12: *The heading "Programmed 2D and 3D topochemical polymerization-based photocarving" and the description is mis-leading. topochemical 2D and 3D topochemical polymerization refer to 2D polymer /3D polymer synthesis via topochemical polymerization. What authors have achieved is just photocarving in a plane or 3D. The heading and the text should be rephrased.*

Response 12: We thank the reviewer for the comment. We have revised the heading to "Programmed 2D and 3D nanostructures prepared by topochemical polymerization-based photocarving" to more accurately reflect the content.

Comment 13: *Page 10: large-are should be large-area*

Response 13: We have revised the expression as "large-area" in the revised manuscript.

"By steering the position of laser spot, we applied a programmed arbitrary 2D irradiation pattern (Fig. 4a) on the large-area single-crystalline film of **1** using a 488 nm laser (8 μW, spot diameter ≈ 400 nm, exposure time = 50 ms/point)."

Comment 14: *Authors stated that "In contrast, no topochemical polymerization occurred on single-crystalline film photoresists in the absence of oxygen". What is the role of oxygen in topochemical polymerization? If the benzimidazole radical is formed under photoirradiation, why can't it just dimerize? Does it mean that the benzimidazole units are connected via peroxy linkage than N-N single bond? The higher oxygen content after photoirradiation is also supportive of this argument. Additionally, the distance between heterocyclic rings in Fig 2H also suggested a larger separation.*

Response 14: We thank the reviewer for the insightful comment. Oxygen plays a key role by capturing the excited-state electrons of the stacking molecules **1**, generating singlet oxygen and benzimidazole radicals, which then initiate subsequent oxidation reactions. In the absence of oxygen, the excited-state molecules relax back to the ground state and thus no reaction occurs. Based on the intermolecular distance between benzimidazole moieties in neighboring molecules, as the reviewer suggested, we have re-examined the photoinduced coupling pathway. As suggested by the reviewers, the formation of N-O-O-N coupled polymeric products is more plausible. The 3.3 Å distance between nitrogen atoms is more consistent with the formation of such N-O-O-N bridges rather than merely N-N. In the revised mechanism, benzimidazole radicals first react with reactive oxygen species, particularly singlet oxygen, to form peroxyl intermediates, which then couple with adjacent benzimidazole radicals. This revised pathway can be supported by transient absorption spectroscopy (formation of benzimidazole radicals), and mass spectrometry data (oxidation of benzimidazole moieties).
Accordingly, we have updated the mechanistic illustrations in Figures 2h, 3f, and Supplementary Figure 32, included a description of possible oxidation at the alkyl positions, and added new relevant references to the discussion section.

“The formation of an acidic polymeric products and cross-linked bridging -O-O- bonds introduce sufficient polarity differences compared to the unpolymerized monomers, facilitating selective removal of the polymeric products upon irradiation.”

Responses to the Reviewers' Comments:

We have addressed the Reviewer's comments in the order R2.

Reviewer 2 (R2):

Revisions suggested by R2 and our responses are outlined below:

Comment 1: *I found that the authors made efforts to address the questions and comments. My only remaining concern is the structural feasibility of the proposed covalent linkage between monomer 1 molecules. The authors suggest the formation of an "N–O–O–N" bond; however, in light of Ref. 81 (S. Hatano et al., J. Am. Chem. Soc. 2013, 135, 3164–3172), it seems plausible that alternative bond structures could form between the benzimidazole moieties of neighboring monomers, for example a "C-O-O-C" or "C-O-C" bond. Therefore, it is strongly recommended that the authors experimentally confirm the nature of the covalent bond formed between two monomer 1 molecules before publication.*

Response 1: We thank the reviewer for this insightful and constructive comment. To clarify the nature of the covalent bond formed after coupling mediated by reactive oxygen species, we investigated the chemical bonding composition using *in-situ* infrared (IR) spectroscopy during the photoreaction process. As shown in Figure **a** (below), the *in-situ* IR spectra reveal the emergence of a new peak at approximately 1570 cm^{-1}, which can be reasonably assigned to the vibration of an N-O single bond, according to established IR spectral assignments for N-O functionalities (Miller, L. M.; Coates, J. P. Interpretation of Infrared Spectra: A Practical and Systematic Approach, 2026). As the photoreaction proceeds, the growth of the peak at 1570 cm^{-1} gradually levels off (Figure **b**), suggesting the completion of the coupling process and the formation of a polymerized network structure.
We agree with the reviewer that, in principle, the benzimidazole radical of monomers **1**, in the presence of reactive oxygen species, could also lead to alternative bond motifs, such as C-O-O-C-type peroxide linkages, analogous to superoxide-derived organic peroxides. If such C-O-O-C linkages were formed, characteristic vibrational modes associated with peroxide bonds would be expected in the range of 1100 to 1250 cm^{-1} region. This assignment is well documented in prior IR studies of organic peroxides (Appl. Spectrosc. 62, 906–915,2008; and Analytical Chemistry 23, 282-285,1951). However, our *in-situ* IR measurements do not show discernible changes or characteristic absorption features in this spectral region during the reaction (Figure c), suggesting that the formation of C-O-O-C or C-O-C linkages is less likely under the present conditions.

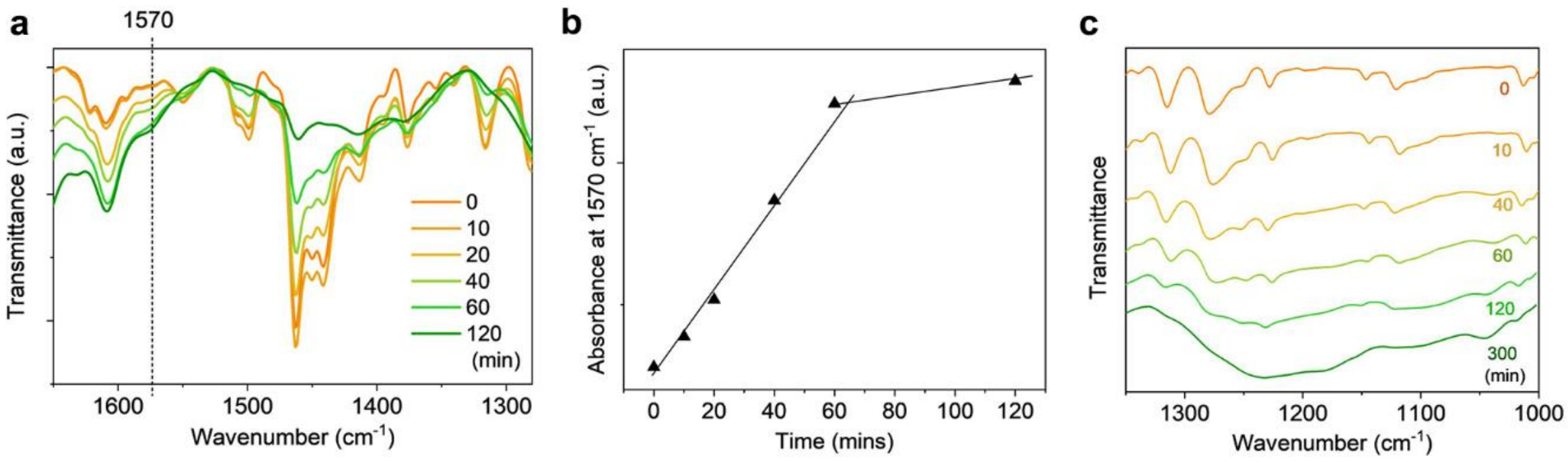

Figure. *In-situ* infrared analysis of the photoinduced polymerization process. a-b, *In-situ* infrared spectra of a large-area single-crystalline film photoresist collected under different laser irradiation durations (480 nm, 50 μW). A new peak emerges at approximately 1570 cm^{-1} and evolves with irradiation time, which can be assigned to vibrations associated with N-O bonding[7]. This observation indicates the formation of oxygen-bridged linkages involving nitrogen atoms during the photoinduced polymerization process. **c**, *In-situ* infrared spectra of

a large-area single-crystalline film photoresist collected in the wavenumber range from 1000 to 1350 cm^{-1}.

Taken together, these spectroscopic observations are consistent with a coupling pathway that preferentially involves the nitrogen sites of the benzimidazole moieties, supporting the proposed N-O-O-N-type connectivity. To address the reviewer's concern more explicitly, we have included the corresponding infrared absorption spectrum data as Supplementary Figure 32 in the revised supplementary information.

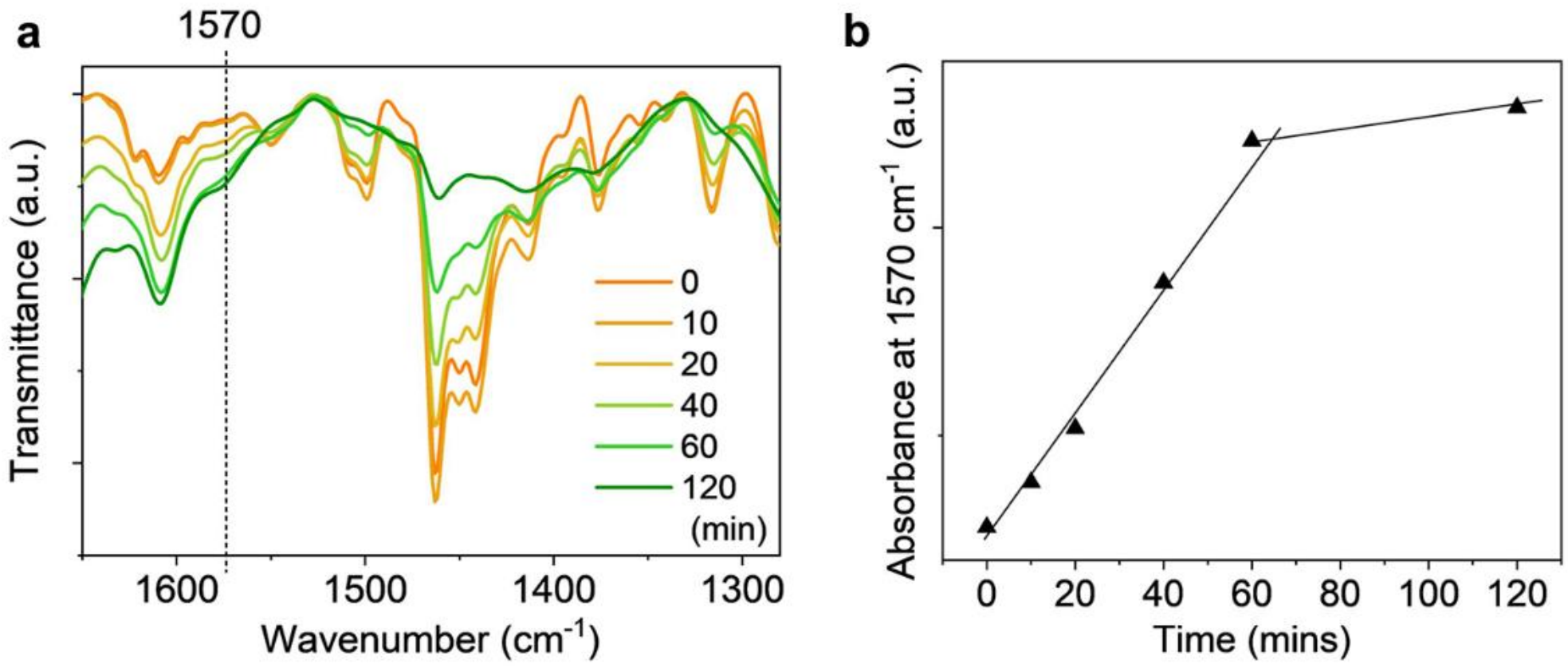

Supplementary Figure 32. ***In-situ*** **infrared analysis of the photoinduced polymerization process. a-b,** *In-situ* infrared spectra of a large-area single-crystalline film photoresist collected under different laser irradiation durations (480 nm, 50 μW). A new peak emerges at approximately 1570 cm^{-1} and evolves with irradiation time, which can be assigned to vibrations associated with N-O bonding. This observation indicates the formation of oxygen-bridged linkages involving nitrogen atoms during the photoinduced polymerization process.

Reviewer 3 (R3):

Comment 1: *Authors have addressed all the comments raised by me in a satisfactory manner. The explanations and additions in the manuscript are convincing. Now the paper has become much better. I recommend acceptance.*

Response 1: We sincerely thank the reviewer for the positive evaluation and recommendation for acceptance. We appreciate the helpful and constructive comments provided during the review process, which have improved the quality of the manuscript.
